# Smart Electronic Textile‐Based Wearable Supercapacitors

**DOI:** 10.1002/advs.202203856

**Published:** 2022-10-03

**Authors:** Md Rashedul Islam, Shaila Afroj, Kostya S. Novoselov, Nazmul Karim

**Affiliations:** ^1^ Centre for Print Research (CFPR) The University of the West of England Frenchay Campus Bristol BS16 1QY UK; ^2^ Institute for Functional Intelligent Materials, Department of Materials Science and Engineering National University of Singapore Singapore 117575 Singapore; ^3^ Chongqing 2D Materials Institute Liangjiang New Area Chongqing 400714 China

**Keywords:** electronic textiles, energy storage devices, smart textiles, supercapacitors, wearable electronics

## Abstract

Electronic textiles (e‐textiles) have drawn significant attention from the scientific and engineering community as lightweight and comfortable next‐generation wearable devices due to their ability to interface with the human body, and continuously monitor, collect, and communicate various physiological parameters. However, one of the major challenges for the commercialization and further growth of e‐textiles is the lack of compatible power supply units. Thin and flexible supercapacitors (SCs), among various energy storage systems, are gaining consideration due to their salient features including excellent lifetime, lightweight, and high‐power density. Textile‐based SCs are thus an exciting energy storage solution to power smart gadgets integrated into clothing. Here, materials, fabrications, and characterization strategies for textile‐based SCs are reviewed. The recent progress of textile‐based SCs is then summarized in terms of their electrochemical performances, followed by the discussion on key parameters for their wearable electronics applications, including washability, flexibility, and scalability. Finally, the perspectives on their research and technological prospects to facilitate an essential step towards moving from laboratory‐based flexible and wearable SCs to industrial‐scale mass production are presented.

## Graphical abstract

This review provides an overview of materials, fabrications, and characterization strategies for smart electronic textile‐based wearable supercapacitors. Recent progress in terms of their electrochemical performances is then summarized, followed by a discussion on key parameters for wearable applications. Finally, the perspectives on their technological prospects to facilitate an essential step towards moving from R&D‐based supercapacitors to real‐world applications are presented.

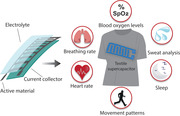

## 1 Introduction

**Figure 1:**
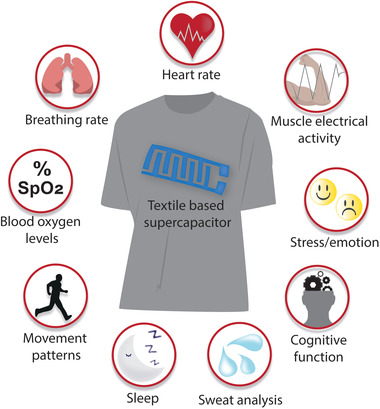
Textile‐based flexible supercapacitors for powering up wearable devices to monitor physiological parameters.

Wearable electronic textiles (e‐textiles) have been going through significant evolutions in recent years, due to the continuous progress of material science and nanotechnology, miniaturization, and wireless revolution.^[^ ^1^, ^2^ ^]^ E‐textiles possess functionalities such as sensing, computation, display, and communication,^[^ ^3^, ^4^, ^5^, ^6^ ^]^ which facilitate the manufacturing of highly innovative and intelligent garments, able to perform as sensors, actuators, power generators, and energy storage devices all at the same time.^[^ ^7^, ^8^ ^]^ Combining these electronic fibers/textiles with human skin can potentially build an intelligent system that could be integrated with biological nerves, muscles, and ligaments in the future to endow us with more functions.^[^ ^9^ ^]^ E‐textiles inherit the advantages of being lightweight, flexible, and air permeable with a certain degree of ductility of traditional fibers/textiles while possessing electronic functions.^[^ ^10^, ^11^ ^]^ As a lightweight portable device to monitor vital health parameters (**Figure** **1**), e‐textiles have become a focus of research interest due to their prospects in sportswear, military uniforms, safety instruments, environmental monitoring, and health care applications.^[^ ^12^, ^13^, ^14^ ^]^ However, one of the key challenges to integrate such electronic devices into textiles is the requirement of a lightweight, flexible, and high‐performance power supply unit.^[^ ^15^, ^16^ ^]^

**Figure 2:**
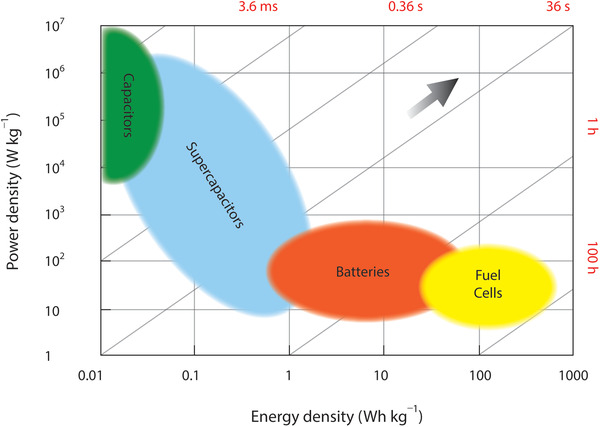
Ragone plot showing comparison of different electrochemical energy storage systems.

Conventional energy storage devices (e.g., batteries) can store a large amount of energy that cannot be delivered quickly owing to their higher internal resistance. Capacitors are another type of energy storage device, which can be charged and discharged quickly. However, capacitors have limited storage capacity. Therefore, the development of capacitors with high energy densities (i.e., supercapacitors) has become an exciting area of research for electrochemical energy storage/conversion systems. Supercapacitors (SC), also referred to as ultracapacitors, are promising electrochemical energy storage devices that can be charged and discharged within seconds, and possess high power density, long cycle life, and outstanding cyclic stability.^[^ ^17^ ^]^ As a relatively new type of capacitors, they are distinguished by the phenomenon of electrochemical double‐layer, diffusion, and large effective area which lead to extremely large capacitance per unit of geometrical area, taking their place in‐between batteries and conventional capacitors. Considering energy and power densities, they also possess a wide area between batteries and conventional capacitors (**Figure** **2**). The incorporation of flexible electrodes and/or substrate materials in SCs provides structural flexibility with their inherent high‐power density, which are highly attractive for a large number of emerging portable and lightweight consumer devices.^[^ ^18^ ^]^ Flexible plastic, elastomeric and textile substrates possess better biocompatibility, stretchability, transparency, and wearability.^[^ ^19^ ^]^ In addition to intrinsic wearability and flexibility, a textile‐based SC ensures better comfort when worn, better integration with the garment, and better wearability of the electronic components in comparison to the conventional rigid and bulky power supply units. It also ensures the enhanced mass loading of active materials, resulting in higher capacitance, energy, and power density. Therefore, textile‐based flexible SCs show great potential for wearable electronic applications, due to miniaturized, portable, and flexible consumer electronics in comparison with the current energy storage devices.^[^ ^20^ ^]^

While several flexible substrates can be exploited for SC fabrication, this review focuses on textiles‐based flexible and wearable SCs due to their potential for next‐generation wearable e‐textiles applications. First, we present an overview of electrochemical energy storage technologies and their working principles. We then discuss the basic parameters to evaluate SC performances. Additionally, we summarize suitable textile substrates as well as electroactive materials required for the preparation of conductive textile electrodes and electrolytes for SC fabrications. We then review manufacturing techniques for conductive textile‐based electrodes, followed by various forms of textile‐based SCs and their integration techniques. We also summarize the energy storage performances of recently developed textile‐based SCs in terms of capacitance, energy density, and power density. Furthermore, key properties of textiles‐based SCs for wearable e‐textiles applications such as flexibility, safety, and washability are discussed. Finally, we conclude our review with recommendations on future research directions for textile‐based SCs.

## 2 Overview of Electrochemical Energy Storage System

Energy storage is defined as the conversion of electrical energy from a power network into a form, that can be stored until converted back to its original electrical form.^[^ ^21^ ^]^ The purpose of such a system is to capture produced energy for later use,^[^ ^22^ ^]^ offering a number of significant benefits including achieving demand‐side energy management, improved stability of power quality, and the reliability of power supply on a long‐term basis.^[^ ^23^ ^]^ With the intensified energy crisis in recent years, energy storage has become a major research focus in both industry and academia^[^ ^24^ ^]^ and is viewed as a promising solution for future highly renewable power systems.^[^ ^25^ ^]^ Among the various forms of energy storage, electrochemical energy storage (EES) systems are vital, due to their versatility from assisting very large‐scale electrical grids down to tiny portable devices to be used for various purposes.^[^ ^26^, ^27^ ^]^ They offer the electrical energy accumulation for longer durability (even over 10^6^ cycles) and higher specific power (more than 10 kW kg^−1^), making them very useful for short‐term pulses in hybrid electrical vehicles, digital telecommunications systems, uninterruptible power supply (UPS) for computers, pulse laser techniques, etc.^[^ ^28^ ^]^ The electric energy is stored in the chemical bonds of electrode materials of the device, which involves the conversion reaction between chemical and electric energy. Nowadays, EES devices are an integral part of telecommunication systems (cell phones, remote communication, walkie‐talkies, etc.), standby power systems, and electric hybrid vehicles in the form of storage components such as batteries, SCs, and fuel cells.^[^ ^29^ ^]^

Two main parameters are important for energy storage: energy density and power density. The energy density is defined as the amount of energy to be stored per unit volume or weight, and the power density is described as the speed at which energy is stored or discharged from the device. An ideal storage device should simultaneously possess both high energy and power densities. Batteries and fuel cells are typical EES devices of small specific power, while conventional capacitors can have higher specific power but exhibit a very low specific energy. The performance parameters of EES such as energy density, power density, and safety mostly depend on the electrode materials, which should have high electro activity, high electron/ion conductivity, and high structure/ electrochemical stability.^[^ ^30^ ^]^ Many efforts have been made to develop advanced electrode materials in the last few decades, however, it still requires further development regarding energy density, power density, and lifespan. Additionally, it is desirable to fabricate EES with high electrochemical performance, ultra‐flexibility, and lightweight for wearable electronics applications.^[^ ^31^ ^]^

### 2.1 Structure of EES Devices

**Figure 3:**
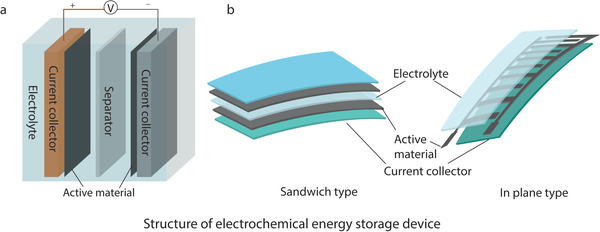
Schematic diagram of the structure of electrochemical energy storage devices. a) Conventional rigid form and b) flexible form.

**Figure 4:**
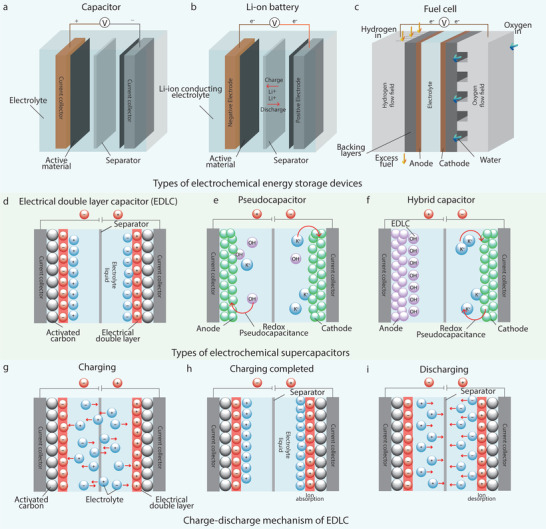
Basic schematic of electrochemical energy storage devices: a) a capacitor, b) a Li‐ion battery, and c) a fuel cell. Types of electrochemical supercapacitors: d) EDLC, e) Pseudocapacitor, f) Hybrid capacitor, and g–i) Charge‐discharge mechanism of an EDLC.

EES devices usually consist of electrode material, current collector, separator, and electrolyte.^[^ ^32^ ^]^ A thin layer of separator, sandwiched by a pair of electrodes and current collectors, filled with electrolyte make the device, **Figure** **3**. Electrodes are composed of electrochemically active materials which store charges. Current collector, made of electrically conductive substrates, connects the electrodes with the external circuitry for the charge transfer. Separators physically and electrically separate electrodes to avoid short circuits. Electrolytes, either in liquid or in gel form, are used to carry and transport charged ions between electrodes. Finally, an encapsulation layer is applied to protect the full integration from any leakage of electrolyte and oxidation of any material, ensuring the stability and safety of the device.^[^ ^33^ ^]^ **Figure** **4**(a–c) represents the schematic of the basic structural components of different EES devices.

### 2.2 Types and Working Principles of EES

EES devices are primarily classified as electrochemical capacitors (Figure 4a), batteries (Figure 4b), and fuel cells (Figure 4c). Due to a comparatively bulk structure, fuel cell is not considered suitable for wearable applications. Therefore, EES devices that are used in wearable systems, may either be electrochemical capacitors or batteries. Electrochemical capacitors, also known as supercapacitors (SCs) or ultracapacitors, can be charged and discharged quickly with nearly 100% efficiency. They possess outstanding power performance, good reversibility, and a very long cycling life (>100 000 cycles). **Table** **1** compares the characteristics of various EES devices. Depending on the use of electrode materials, SCs are further divided into electrostatic double‐layer capacitors (EDLCs), pseudocapacitors, and hybrid capacitors. There are two charge storage mechanisms involved in the operation of SCs: storing the charges electrostatically (at the interface of capacitor electrode as electric double layer capacitance) and storing the charges faradaically (at the electrode surface as pseudocapacitance).^[^ ^34^ ^]^ **Table** **2** compares the properties among various SC types.

**Table 1:** Comparison among various EESDs.^[^ ^35^, ^36^ ^]^

Characteristics	Li‐ion battery	Capacitor	Supercapacitor	Fuel cell
Storage mechanism	Chemical	Physical	Physical and chemical	N/A
Energy storage	High	Limited	Limited	High
Energy density [Wh kg^−1^]	8–600	0.01–0.05	1–10	300–3000
Power density [kW kg^−1^]	0.005 to 0.4	0.25–10 000	10–120	0.001–0.1
Charge/discharge time	1–10 h	ps‐ms	ms‐seconds	10–300 h
Operating temperature	−20 to +65 °C	−20 to +100 °C	−40 to +85 °C	+25 to +90 °C
Operating voltage	1.25 to 4.2V	6 to 800V	2.3 to 2.75V	0.6V
Cycle‐life	150–1500	>100 000	>50 000+ h, unlimited	1500–10 000 h
Charge‐discharge efficiency [%]	70–85	100	85–98	60
Charge stored determinants	Active mass and thermodynamics	Electrode area and dielectric	Electrode microstructure and electrolyte	N/A

**Table 2:** Comparison among various types of supercapacitors.^[^ ^45^, ^46^, ^47^ ^]^

Parameters	Electric double layer capacitor (EDLC)	Pseudo‐capacitor (PC)	Hybrid capacitor (HC)
Charge storage mechanism	Physical – Non‐faradic/electrostatic, electrical charge store at electrode/electrolyte interface	Chemical‐ faradic, reversible redox reaction	Physical and chemical (both faradic and non‐faradic)
Electrode materials	Carbonaceous compounds	Conductive polymers and metal oxides	Combination of EDLC and PC‐type materials
Specific capacitance [Fg^−1^]	Lower (200–300)	Higher (200–1340)	Higher (50–1893)
Energy density [Wh kg^−1^]	Low (6.8–12)	High (167–223)	High (132–231)
Cyclability [cycles]	High (100 000)	Low (5000)	Medium (12 000)
Capacitance retention [%]	60–100	52–96	80–95

#### 2.2.1 Electrical Double‐Layer Capacitors (EDLCs)

An electric double layer (EDL) or Helmholtz double‐layer (attributed to Helmholtz) involves the formation of two charged layers at the electrode‐electrolyte interface. Thus, the ability to store potential‐dependent charge is termed as electric double layer capacitance, and the SCs based on this principle are termed as electric double layer capacitors (EDLCs). From a structural view, they consist of three parts: two active material‐loaded electrodes, an electrolyte, and a separator sheet,^[^ ^37^ ^]^ Figure 4d. Energy is stored through charge separation and can keep considerably more energy than a classic capacitor. A simple movement of ions migrating to and releasing from electrode surfaces is involved (Figure 4g–i), therefore can respond rapidly.^[^ ^38^ ^]^ EDLCs are usually evaluated in terms of Farads (F), instead of picofarads (pF) and microfarads (µF) for the conventional dielectric and electrolytic capacitors due to their ability to store much more electricity. EDLCs have high power density, good reversibility, and long cycle life, achieved by the use of high‐surface‐area activated carbon (AC) as the working medium in the capacitor system.^[^ ^34^ ^]^

#### 2.2.2 Pseudo Capacitors

Pseudo capacitors defeat EDLCs in energy density for the reversible redox reactions between their electrode materials and electrolytes,^[^ ^39^ ^]^ Figure 4e. They are also referred to as redox supercapacitors, since they store charges, faradaically, through battery‐like redox reactions but at a faster rate than the EDLCs, offering a pathway for achieving both high energy and high‐power densities. Materials that combine these properties are in demand for the realization of fast‐charging EES devices capable of delivering high power for a long period of time.^[^ ^40^ ^]^ Transition metal oxides such as MnO_2_, conductive polymers like Polyaniline (PANI), Polypyrrole (PPy), or derivatives of Polythiophene (PTh) such as Poly (3,4‐(ethylenedioxy) thiophene) (PEDOT) are being studied as prominent pseudocapacitive materials nowadays. This faradaic charge transfer process is highly reversible. During charging, the surface region of redox‐active electrode materials gets reduced to lower oxidation states coupled with adsorption/insertion of cations from the electrolyte at/near the electrode surfaces. Upon discharge, the process can be almost fully reversed,^[^ ^34^ ^]^ similar to the charging and discharging processes that occur in batteries, resulting in faradaic current passing through the SC cell.^[^ ^41^, ^42^ ^]^ Pseudo capacitors offer a higher energy density but a lower cycle life than EDLCs.

#### 2.2.3 Hybrid Capacitors

Hybrid SCs offer improved performance in energy density without altering the power density and have been in recent trends. They deliver higher specific capacitance in comparison to the existing EDLC and pseudocapacitors.^[^ ^43^ ^]^ They are made by the hybridization of two types of electrodes to form a new capacitor, Figure 4f. This is a unique approach, which is used to enhance the electrochemical properties of a single cell. The exhibition of electrochemical behavior over a wide voltage range will enhance the overall operating voltage window and increase specific energy density, which is larger than the cells containing a single type of electrode. Among two types of electrodes in hybrid capacitors, one is an energy source electrode (i.e., battery‐like electrodes), and the other terminal contains a power source electrode (i.e., either an EDLC or a pseudo capacitor electrode). The selection of the energy source electrode is important to enhance the cell voltage without sacrificing much energy and power densities. Such configuration offers the advantages of both SCs and advanced batteries, resulting in a significant increase in the overall energy density of the system.^[^ ^44^ ^]^

#### 2.2.4 Lithium‐Ion Batteries

Batteries store charge through the conversion of electrical energy into chemical energy. In a lithium‐ion battery (LIB), lithium ions move from the negative to the positive electrode during discharge, and travel back to the negative electrode when charging, Figure 4b. Unlike lithium primary batteries (which are disposable), LIBs use an intercalated lithium compound as the electrode material, instead of metallic lithium. LIBs are common in consumer electronics as rechargeable batteries for portable electronics, which provide one of the best energy‐to‐weight ratios, high open circuit voltage, low self‐discharge rate, no memory effect, and a slow loss of charge when not in use. Beyond consumer electronics, LIBs are growing in popularity for military, electric vehicle, and aerospace applications due to their high energy density.^[^ ^48^, ^49^ ^]^ The diffusion‐controlled electrochemical process of lithium‐ion insertion/de‐insertion in LIBs results in a much lower power density compared to SCs. However, they typically appear in a rigid form which makes them unfavorable for many applications, especially in the field of portable and highly integrated equipment. Several research groups also investigated flexible textile‐based batteries for wearable electronics applications.^[^ ^50^, ^51^, ^52^, ^53^, ^54^ ^]^ Although this review does not focus on batteries, it is worth noting that in many case, batteries are used in combination with supercapacitors for achieving high performance.

### 2.3 Electrochemical Performances of Supercapacitor

A series of key parameters, and various techniques are used for the evaluation of the electrochemical performance of a SC. Among them, widely used techniques are: Cyclic voltammetry (CV), galvanostatic charge/ discharge (GCD), and electrochemical impedance spectroscopy (EIS). Three fundamental parameters (voltage, current, and time) for SC can be measured by all these techniques. Additionally, other SC performance metrics including capacitance, equivalent series resistance (ESR), operating voltage, time constant, energy, and power performance can be derived from those parameters.

#### 2.3.1 Cyclic Voltammetry (CV)

**Figure 5:**
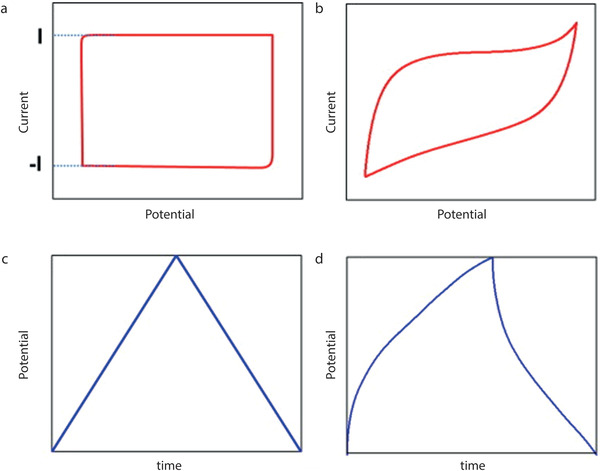
Typical a) CV curves and c) galvanostatic charge‐discharge (GCD) curves for ideal supercapacitor; b) CV curve and d) GCD curve distortion due to faradaic reactions.^[^ ^55^ ^]^

CV is a powerful and popular electrochemical technique commonly employed to investigate the reduction and oxidation processes of molecular species.^[^ ^56^ ^]^ Such technique is used to study the electrochemical properties related to electroactive surfaces, and characterize the electrode materials primarily.^[^ ^57^, ^58^ ^]^ In this technique, a linearly changed electric potential is applied against time to measure the current. The graphical analysis of a cyclic voltammogram provides redox peaks (reduction and oxidation peaks of the material) and predicts the capacitive behavior of the electrode. Therefore, the potential at which the material is oxidized and reduced can be found in this technique.^[^ ^34^, ^59^ ^]^ A typical cyclic voltammogram for an electrochemically reversible and diffusion‐controlled redox process is shown in **Figure** **5**a. The curves obtained through CV for both EDLCs and pseudocapacitors are evaluated to measure the capacitance (C) of the material deposited over the electrode by using Equation (1). The shape of the resulting CV curves for an ideal SC is rectangular. However, the variation in the shape and size of the plot can occur when the deposited materials over the electrode are dissolved into the electrolyte. It can also happen due to the detachment of the electrode contacts during cyclic repetition, Figure 5b.^[^ ^60^ ^]^ The gravimetric capacitance (Equation (4)), lengthwise capacitance (Equation (6)), areal capacitance (Equation (8)), volumetric capacitance (Equation (10)), energy density (Equation (12)) of the electrode or total SC cell can be obtained via integration of CV curves.^[^ ^34^ ^]^

#### 2.3.2 Galvanostatic Charge Discharge (GCD)

Galvanostatic charge‐discharge (GCD) test is considered as the most accurate and versatile approach, and the most widely used method for capacitance assessment (Equation (2)).^[^ ^60^ ^]^ The direct current (DC) at a constant level is imparted in this method for repetitive charging and discharging of the SC device or the working electrode. A potential versus time plot is obtained from this method, Figure 5c. Additionally, the cyclic stability of SC devices can be studied from GCD. The symmetric curves obtained from the charge‐discharge through GCD confirm the capacitive behavior of the device, enlightening capacitance as the function of applied voltage. Additionally, gravimetric capacitance (Equation (5)), lengthwise capacitance (Equation (7)), areal capacitance (Equation (9)), and volumetric capacitance (Equation (11)) for SC materials can also be obtained via GCD.^[^ ^34^ ^]^

#### 2.3.3 Electrochemical Impedance Spectroscopy (EIS)

Electrochemical Impedance Spectroscopy (EIS), an electroanalytical method, measures the impedance of a power cell as a function of frequency by applying the alternating current (AC) instead of the DC. The fundamental approach of EIS is the application of a spectrum of small‐amplitude sinusoidal AC voltage excitations to the system. The frequency of the AC signal is varied, and the overall impedance of the cell is recorded as a function of frequency. The resulting data are usually expressed graphically in two types of plots: a) the Nyquist plot, which shows imaginary versus real impedance at different frequencies, and b) the Bode plot, which shows absolute impedance versus frequency. For SC materials, EIS testing can be used to study the impedance, charge transfer, mass transport, and charge storage mechanisms as well as to estimate the capacitance (Equation (3)), energy, and power properties.^[^ ^34^, ^58^ ^]^ A summary of technical merits and demerits of several characterization techniques is presented in **Table** **3**.

**Table 3:** Technical merits and demerits of the CV, GCD, and EIS techniques.^[^ ^60^, ^61^ ^]^

Techniques	CV	GCD	EIS
Principle	CV is varying the potential against time and measuring the current	GCD is applying a positive or negative current against time and measuring the voltage	Measuring impedance of a power cell as a function of frequency by applying alternating current (AC)
Merits	Degradation process Specific capacitance Differentiate between EDLC and PC	Capacitance calculation Differentiate between EDL and PC	Resistance calculation Specific capacitance calculation Differentiate between resistive and inductive nature Nondestructive technique Relaxation time for recharging Exhibit Degradation behavior
Demerits	Show only kinetic aspects; thermodynamic aspect is neglected	Exhibit same triangular shape for all double‐layer capacitive materials	Evaluation at small voltage only Discrete behavior above 10^6^ Hz

### 2.4 Key Metrics for Supercapacitor Performances

The key parameters used to evaluate the electrochemical performances of a SC are capacitance, operating voltage, ESR, power density, energy density, and time constant. Capacitance is defined as the ratio of the charge stored (or separated) to the potential difference between the conductors.^[^ ^62^ ^]^ The total charge storage ability of a SC device is termed as the capacitance, which is calculated from the formula stated in **Table** **4** (Equations (1)–(3)). It is noteworthy that, while specifying the capacitance of SC, a more intrinsic specific capacitance is measured in terms of the mass of the electroactive materials or length, area, and/or volume of the SC device (Equations (4)–(11)). The other two important parameters for evaluating SC performances are: energy density and power density. Energy density, derived from Equation (12), denotes the amount of energy that can be delivered from a SC. The power density denotes how faster the energy can be delivered by a SC and can be calculated from Equation (13), Equation (14), or Equation (15).

**Table 4:** Key metrics used for the characterization of a supercapacitor

Parameters (Unit)	Information obtained	Measurement formula	Equation
Capacitance [F]	Ability to collect and store energy in the form of electrical charge	$C=\frac{\int IdV}{V}$ $C=\frac{i\;\Delta V}{\Delta t}$ $2\pi C=\frac{d\;(-Z^{\prime \prime })}{d\left(\frac{1}{f}\right)}$	(1) (2) (3)
Gravimetric capacitance [F g^−1^]	Charge storage ability per unit mass	$C_{m}=\frac{A}{2\;s\;m\;V}$ $C_{m}=\frac{i\;\Delta t}{m\;\Delta V}$	(4) (5)
Lengthwise capacitance [F cm^−1^]	Charge storage ability per unit length	$C_{l}=\frac{A}{2\;s\;l\;V}$ $C_{l}=\frac{i\;\Delta t}{l\;\Delta V}$	(6) (7)
Areal capacitance [F cm^−2^]	Charge storage ability per unit area	$C_{A}=\frac{A}{2\;s\;a\;V}\;$ $C_{A}=\frac{i\;\Delta t}{a\;\Delta V}$	(8) (9)
Volumetric capacitance [F cm^−3^]	Charge storage ability per unit volume	$C_{v}=\frac{A}{2\;s\;v\;V}$ $C_{v}=\frac{i\;\Delta t}{V\;\Delta V}$	(10) (11)
Energy density [Wh kg^−1^]	Amount of energy able to deliver	$E=\frac{1}{2}{\mathrm{CV}}^{2}=\frac{QV}{2}$	(12)
Power density [W kg^−1^]	How faster is the energy to deliver	P = VI $P=\frac{E}{t}$ $P=\frac{V^{2}}{4R}$	(13) (14) (15)
Coulombic efficiency	Reversible capacity	$\%E=\frac{C_{\mathrm{charging}}}{C_{\mathrm{discharging}}}\times 100$	(16)

[C = capacitance, I = current density, V = voltage window, i = discharging current, ∆v = discharge voltage, ∆t = discharge time, −Z′ = imaginary part of the impedance, A = integrated area of the CV curve, s = scan rate (mV s^−1^), m = mass of the electroactive material on both electrodes, l = length of the electrode, v = volume of the SC, R = resistance].

**Figure 6:**
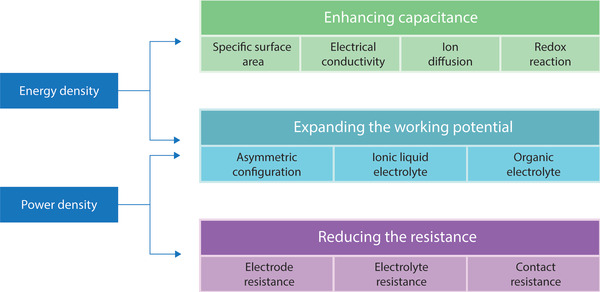
Approaches for enhancing energy and power densities of supercapacitor.^[^ ^64^ ^]^

Among the performance metrics for all kinds of energy storage and conversion systems, power density and energy density are the most often used parameters for their performance evaluation for all kinds of applications. Compared to batteries, SCs suffer from lower energy density.^[^ ^63^ ^]^ The energy density depends on the capacitance and working voltage window (V). Therefore, increasing the capacitance or extending operating voltage window will enhance the energy density of a SC. Power density depends on their working voltage window (V) and internal resistance (R). Therefore, in addition to extending the working voltage window, one of the ways to increase the power density is by the reduction of internal resistances of SC components. **Figure** **6** summarizes the approaches to improve the energy and power density of SCs.^[^ ^64^ ^]^ Additionally, the long cycle life of SC devices is one of the highly desirable characteristics for certain applications. However, the cycle life, when extremely long, is difficult to measure directly. Therefore, the capacitance retention rate is used as an indirect measurement to estimate the cycle life of a SC. By comparing the capacitance after being given thousands of cycles with that of the first cycle in GCD test, the capacitance retention value is obtained.^[^ ^34^ ^]^

## 3 Components of Textile‐Based Supercapacitors

The performance of SC largely depends on the nature of electrode materials, type of electrolyte used, and the range of voltage windows employed. In this section, we will discuss about the basic textile materials used for wearable SC fabrication, as well as the electroactive materials for electrode preparation and electrolyte materials commonly used for textile‐based SC fabrication.

### 3.1 Textiles as the Substrate for Supercapacitor Fabrication

Multifunctional wearable electronics require a conformal platform close to the human body. Textiles or fabrics that are usually embedded with normal clothes and worn on various body parts, have emerged as promising substrates and platforms for wearable electronics, due to their unique characteristics including lightweight, soft, flexible, stretchable, air‐permeable, low‐cost, chemically resistant, scalable production, and integrable with various forms of garments.^[^ ^65^ ^]^ In addition to natural fibers (e.g., cotton, silk, wool), other polymeric substrates are commonly used to fabricate e‐textiles including poly (ethylene terephthalate) or polyesters (PET), polyamide or nylons (PA), polyimide (PI), viscose, polyethylene naphthalate (PEN) and thermoplastic polyurethane (TPU). Furthermore, some research groups studied papers (specifically for fabricating disposable devices) and polydimethylsiloxane (PDMS) for fabricating such wearable devices.^[^ ^66^, ^67^ ^]^ However, these substrates vary in their physical, chemical, thermal and tensile properties,^[^ ^68^, ^69^ ^]^ **Table** **5**. Therefore, the choice of any specific textile substrate depends on the properties required for the end‐products. In addition to being reusable, cheap, and hydrophilic in nature, textile‐based substrates have many advantages over plastic‐ or paper‐based substrates, when flexibility and stretchability are concerned. For example, the porous structure of textiles provides abundant support for the loading of active materials and facilitates the rapid absorption of electroactive materials due to their hydrophilic nature, resulting in much higher areal mass loading of active materials and higher areal power and energy density. Therefore, low‐cost and highly efficient textile‐based SCs have already been integrated into prototype wearable electronics with a great potential to be used for future high‐tech sportswear, work wear, portable energy systems, military camouflages and health monitoring systems.^[^ ^70^ ^]^

**Table 5:** Typical tensile properties of selected fibers.^[^ ^71^, ^72^ ^]^

Fiber	Moisture regain [%]	Density [g cm^−3^]	Durability	Glass transition temperature [°C]	Tenacity [cN tex^−1^]	Yield stress [cN tex^−1^]	Yield strain [%]	Strain at break [%]
Cotton	8.0	1.54	Fair	220	40	–	–	7
Wool	16.0	1.32	Fair	160	11	6	5	42
Silk	11.0	1.34	Fair	170	38	16	3	23
Nylon	4.5	1.14	Good	70	47	40	16	26
Polyester	0.4	1.38	Excellent	75	47	30	10	15
Viscose	12.5–13.5	1.46–1.54	Good	120	21	7	2	16
Polypropylene	0.05	0.91	Excellent	−25	–	–	–	–

### 3.2 Electroactive Materials for Electrode Preparation

As previously discussed in Section 2.2, SCs are classified into two types, according to the charge‐storage mechanism, which includes EDLCs based on carbon materials, and pseudocapacitors based on certain transition metal oxides or conductive polymers. The EDLCs usually display perfect cycling stability, but lower specific capacitance. In contrast, pseudocapacitors present high specific capacitance but poor cyclability. These undoubtedly limit their practical application as individual electrode materials for SCs.^[^ ^17^ ^]^ Therefore, to enhance the capacitive performance, developing composite materials combining both EDLC materials and pseudocapacitor materials becomes an inevitable trend.^[^ ^73^ ^]^

#### 3.2.1 Carbonaceous Materials

**Figure 7:**
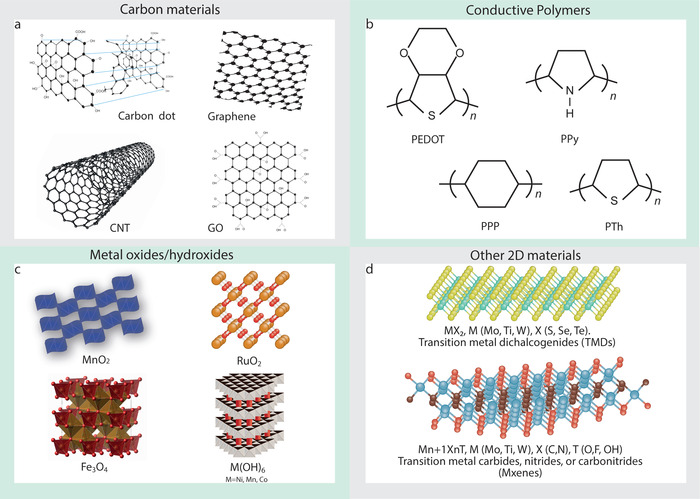
Electrode materials for textile‐based supercapacitors: a) carbonaceous materials, b) conductive polymers, c) metal oxides/hydroxides, and d) other 2D materials.

Carbonaceous compounds and their allotropes (**Figure** **7**a) are currently of particular interest as key materials for multiple applications including nano‐ and optoelectronics, photonics, molecular separation and storage, nanomechanics, catalysis, and energy storage.^[^ ^74^ ^]^ A unique combination of chemical and physical properties, including exceptionally high Young's modulus and mechanical strength, higher light transmittance, higher conductivity, higher surface‐area range (≈1 to >2000 m^2^ g^−1^), good corrosion resistance, higher temperature stability, controlled pore structure, processability and compatibility with composite materials, and relatively lower cost make carbon‐based materials attractive material for SC electrodes.^[^ ^35^, ^75^ ^]^ Among five forms of carbon allotropes: 3D diamond (Csp^3^), 2D graphite (Csp^2^), 1D carbene (Csp^1^), 0D fullerene (Csp^0^), and transitional carbons (admixtures of Csp^3^, Csp^2^, and Csp^1^), the first four are crystalline and first two are found naturally. Graphite and fullerene have attracted much attention as electrode materials due to their structures and functionalities.^[^ ^76^ ^]^ Additionally, they have faster electron transfer kinetics with lower fabrication costs. However, their specific capacitances were found to be too low for commercial applications.

Carbon nanotubes (CNTs), a 1D allotrope of carbon, are cylindrical large molecules consisting of a hexagonal arrangement of hybridized carbon atoms. Being nano‐meter in diameter and several millimeters in length, they are available in the form of single‐walled CNTs, SWCNTs (formed by rolling up a single sheet of graphene) or multiwalled CNTs, MWCNTs (by rolling up multiple sheets of graphene).^[^ ^77^ ^]^ Chemical vapor deposition (CVD), laser‐ablation, and carbon arc‐discharge are three common techniques for producing CNTs. Structure, surface area, surface charge, size distribution, surface chemistry, agglomeration state, and purity are the main parameters that affect the reactivity of CNTs.^[^ ^78^ ^]^ Their exceptional physical, chemical, and electronic properties offer exciting possibilities for even nano‐meter scale electronic applications.^[^ ^29^ ^]^ Since its discovery in 1990s, CNTs have been utilized in a variety of applications including actuators, artificial muscles, and lightweight electromagnetic shields.^[^ ^79^ ^]^ Additionally, CNTs have been investigated as SC electrodes by several research groups.^[^ ^80^, ^81^, ^82^, ^83^ ^]^

Carbon black (CB), a common denomination for particles with a carbonaceous core, is manufactured by thermal decomposition, including detonation, or by incomplete combustion of carbon‐hydrogen compounds having a well‐defined morphology with a minimum content of tars or other extraneous materials.^[^ ^84^ ^]^ In recent years, it has become an interesting modifier of sensors, due to its excellent conductive and electrocatalytic properties, as well as its cost‐effectiveness.^[^ ^85^ ^]^ Conductive CBs which usually possess electrical conductivity in a range of 10^−1^ to 10^2^ (Ωcm)^−1^ are usually well‐structured (i.e., aggregates with a highly branched open structure). They have higher porosity, smaller particle size, and chemically clean (oxygen‐free) surfaces.^[^ ^35^ ^]^ In addition to using it as electrode material itself,^[^ ^86^ ^]^ CB is also used in combination with other materials to enhance the performance of SC.^[^ ^87^, ^88^ ^]^

Activated carbon (AC), in comparison with the other forms of carbonaceous materials, is preferred as electrode materials due to its low cost and environmentally friendly nature.^[^ ^89^ ^]^ Carbonization and activation are the main steps for the synthesis of activated carbon. Due to the tunable pore size and higher specific surface area as compared to other carbonaceous material, activated carbon has been widely used as electrode material for SC applications. The high surface area, hierarchical pore structure, and different morphology enable the formation of a bilayer of ions at electrode‐electrolyte interfaces.^[^ ^90^ ^]^ Activated carbon powder (ACP) is known as an inexpensive yet good electrode material with 1000–2000 m^2^g^−1^ of specific surface area,^[^ ^91^ ^]^ and has widely been studied for SC application.^[^ ^92^, ^93^, ^94^ ^]^

Graphene, since its isolation in 2004, has unveiled a wide range of other similar 2D materials and received much attention from the research community due to their outstanding mechanical, thermal, electrical, and other properties.^[^ ^95^, ^96^, ^97^, ^98^ ^]^ It is a 2D allotrope of carbon, which is the basic structural element of carbon allotropes including graphite, CNTs, and fullerenes.^[^ ^99^ ^]^ It has an isolated single layer of carbon hexagons consisting of sp^2^ hybridized C—C bonding with *π*‐electron clouds.^[^ ^100^ ^]^ It can be considered the “mother” of all graphitic‐based nanostructures, owing to the variety of sizes and morphologies onto which a single graphitic layer can be transformed. It can be wrapped up into the 0D “buckyball” structure, and folded into 1D CNTs. It can also be stacked into multi‐layer graphene sheets.^[^ ^101^ ^]^ Mechanical, thermal, and liquid phase exfoliation, and chemical vapor deposition (CVD) are the most common techniques to manufacture graphene.^[^ ^102^, ^103^ ^]^ Due to its unique physicochemical properties including theoretical specific surface area (2600 m^2^g^−1^), good biocompatibility, strong mechanical strength (130 GPa), excellent thermal conductivity (3000 Wm^−1^K^−1^), high electrical charges mobility (230 000 cm^2^ V^−1^s^−1^) and fast electron transportation makes it not only a unique but also a promising material for next‐generation energy storage applications, particularly SC devices.^[^ ^104^, ^105^, ^106^, ^107^, ^108^ ^]^ Graphene and its derivatives have the capability to form chemical bonds with textiles and therefore, show great potential to be used in smart energy storage textiles SC.^[^ ^109^, ^110^, ^111^ ^]^

Graphene oxide (GO), a derivative of graphene^[^ ^112^ ^]^ can be obtained by treating graphite materials with strong oxidizing agents (potassium chlorate and fuming nitric acid) where tightly stacked graphite layers are loosened by the introduction of oxygen atoms to the carbon,^[^ ^113^ ^]^ forming a single‐layer sheet of graphite oxide^[^ ^114^ ^]^ with strong mechanical, electronic and optical properties, chemical functionalization capability and excellent features such as large surface area, high stability, and layered structure.^[^ ^115^, ^116^, ^117^ ^]^ Based on the degree of oxidation, GO can be a semiconductor or insulator, enabling it to be used in many fields.^[^ ^118^ ^]^ Reduced graphene oxide (rGO), another important derivative of graphene,^[^ ^119^ ^]^ consists of few‐atom‐thick 2D sp^2^ hybridized carbon layers with fewer oxygeneous functionalities and exhibits properties between graphene and GO.^[^ ^120^ ^]^ Though it resembles graphene, containing residual oxygen and other heteroatoms with some structural defects degrade its electric properties.^[^ ^121^ ^]^ While graphene derivatives (GO and rGO) can be produced in a huge quantity in their stable dispersions,^[^ ^122^ ^]^ the major challenge for such materials is the ability to produce high‐quality graphene at a larger scale.^[^ ^123^ ^]^ Hybridization of various carbonaceous compounds is also attractive due to their combined electrochemical properties, which provide enhanced capacitive performances of SC devices.^[^ ^124^, ^125^, ^126^, ^127^, ^128^ ^]^

#### 3.2.2 Conductive Polymers

Conductive polymers (CPs) are organic polymers, that are able to conduct electricity through a conjugated bond system along the polymer chain. In the past two decades, they are extensively explored for energy storage applications due to their reversible faradaic redox nature, high charge density, and lower cost as compared to expensive metal oxides. They are considered as promising electrode materials for flexible SCs.^[^ ^129^ ^]^ Among CPs, polyaniline, polypyrrole, and derivatives of polythiophene have widely been studied as active electrode materials for energy storage devices, Figure 7b.

PANI, a conductive polymer, has been playing a great role in energy storage and conversion devices due to its high specific capacitance, high flexibility, and low cost. It is said that the era of intrinsically conductive polymers (ICPs) started with the invention of polyacetylene. However, PANI attracted much more attention from researchers due to its cheaper monomer compared to polyacetylene and ease of synthesis.^[^ ^130^ ^]^ PANI‐based electrodes for SCs provide multi‐redox reactions, high conductivity, and excellent flexibility.^[^ ^131^ ^]^ However, the inferior stability of PANI limits its application to be used alone in the fabrication of electrodes.^[^ ^132^ ^]^ Therefore, the combination of PANI with other active materials (such as carbon materials, metal compounds, or other polymers) is recommended to overcome such intrinsic disadvantages.^[^ ^133^, ^134^, ^135^ ^]^

Polypyrrole (PPy) is a *π*‐electron conjugated CP, which has been researched widely for energy storage applications due to its good electrical conductivity and environmental stability in ambient conditions. It has shown promise as SC electrodes because of its large theoretical capacitance, good redox properties, superior conductivity, ease of synthesis, nontoxicity, biocompatibility, and high thermal and environmental stability.^[^ ^136^ ^]^ However, the brittleness of PPy limits its practical uses. Nevertheless, their processability and mechanical properties can be improved by either blending PPy with some other fiber polymers or forming copolymers of PPy.^[^ ^137^ ^]^ Thus, PPy‐based composites may provide fibers or fabrics with electrical properties similar to metals or semiconductors.^[^ ^138^ ^]^ The water solubility of pyrrole monomers and much less carcinogenic risks associated with its biproducts compared to PANI, makes PPy a proper material as SC electrodes. However, the poor cyclic stability and poor rate behavior of pristine PPy‐based SCs drastically restrict their practical applications.^[^ ^139^ ^]^ Nevertheless, higher electrochemical performance can be achieved by the introduction of a novel design of nanostructured PPy and its nanocomposites, which is currently being explored widely for SC electrode fabrication.^[^ ^140^, ^141^, ^142^ ^]^

PEDOT is one of the most promising *π*‐conjugated polymers exhibiting some very interesting properties such as excellent conductivity (≥300 S cm^−1^), electrooptic properties, and processability.^[^ ^143^, ^144^ ^]^ PEDOT is highly conductive in its oxidized (doped) state, while in its undoped form is usually nonconductive or shows very little conductivity. Its conductivity can be increased by oxidizing or reducing with a doping agent which introduces positive charges along the backbone structure of PEDOT. These positive charges are later balanced by the anions provided by the doping agent.^[^ ^144^ ^]^ The oxidized or doped form of PEDOT shows very high conductivity, flexibility, low‐cost, and pseudocapacitance. However, the low stability and limited capacitance have limited its industrial applications. Several approaches have been undertaken to tackle these issues including the addition of conducting nanofillers to increase conductivity, and mixing or depositing metal oxide to enhance capacitance.^[^ ^145^ ^]^ Though several studies have reported the electrochemical performance of PEDOT‐based SCs^[^ ^146^, ^147^ ^]^ the polymer mixture with polystyrene sulfonate (PEDOT:PSS) possess a high conductivity (up to 4600 S cm^−1^),^[^ ^148^ ^]^ and can be used as an electrode material for SCs.^[^ ^149^, ^150^ ^]^ The hybridization of PEDOT:PSS with other active materials has also been studied for SC electrode fabrication.^[^ ^150^, ^151^, ^152^, ^153^ ^]^

#### 3.2.3 Metal Oxides

Electrodes composed of metal oxides possess exceptional properties, qualifying them as a suitable engineering material with a wide range of applications including sensors, semiconductors, energy storage, lithium‐ion batteries, and solar cells.^[^ ^154^ ^]^ Metal oxides, due to its wide variety of oxidation states for redox charge transfer, are generally considered as one of the prime candidates for use as electrode materials in SCs,^[^ ^155^ ^]^ Figure 7c.

##### Ruthenium Dioxide (RuO_2_)

Due to its high theoretical specific capacitance value (1400–2000 F g^−1^), RuO_2_ has been extensively recognized as a promising material for SC devices.^[^ ^156^ ^]^ Additionally, it demonstrates highly reversible redox reactions, good thermal stability, high electronic conductivity (300 S cm^−1^), superior cycle lifespan, and high rate capability.^[^ ^155^ ^]^ Despite having such outstanding properties, their higher production cost and agglomeration effects limit its practical applications. Therefore, RuO_2_‐based nanocomposites have widely been studied to optimize the material cost, with simultaneous improvement in the electrochemical performances.^[^ ^156^ ^]^ Several researchers have studied RuO_2_‐based nanocomposites for SC fabrication^[^ ^157^, ^158^ ^]^ as well as for the improvement of the electrochemical performances for next‐generation SCs.^[^ ^159^, ^160^, ^161^ ^]^

##### Manganese Dioxide (MnO_2_)

MnO_2_ is considered as one of the most promising electrode materials for electrochemical capacitors, due to its low cost, high theoretical specific capacitance (≈1370 Fg^−1^), natural abundance, environmental friendliness, and nontoxicity.^[^ ^162^ ^]^ MnO_2_ is a very common material in the battery field, which has long been used as active material for the positive electrode.^[^ ^163^ ^]^ The charge storage mechanism is based on the surface adsorption of electrolyte cations M^+^ (e.g., K^+^, Na^+^, Li^+^) as well as proton incorporation as follows:^[^ ^164^ ^]^

(17) $$\mathrm{Mn}O_{2}+xM^{+}+yH^{+}+\left(x+y\right)e^{-}⇌\mathrm{MnOO}M_{x}H_{y}$$

However, the poor conductivity, much lower actual specific capacitance than the theoretical specific capacitance, poor structural stability, and easy dissolving nature in the electrolyte results in poor cycling ability.^[^ ^165^ ^]^ Therefore, the combination of MnO_2_ with other active components is much preferred by researchers for SC electrode application.^[^ ^166^, ^167^, ^168^ ^]^

##### Nickel Oxide (NiO)

NiO is another attractive conversion reaction‐based anode material in the field of SCs due to its low cost, ease of preparation, nontoxicity, environment friendliness, and high theoretical capacity (≈3750 F g^−1^).^[^ ^169^ ^]^ The pseudocapacitance of NiO is obtained from the following redox reaction:

(18) $$\mathrm{NiO}+{\mathrm{OH}}^{-}⇌\mathrm{NiOOH}+e^{-}$$

Though theoretically advantageous enormously, the relatively poor electrical conductivity and lower specific surface area hinder their practical applications. One possible solution to these problems is to synthesize nanostructures of NiO with large surface areas, which are associated with more faradaic active sites and higher pseudo capacitance. Therefore, various nanostructured forms of nickel oxides such as nanowires, nanoflakes, nanocolumns, nanosheets, porous nanoflowers, and hollow nanospheres were successfully fabricated in the past few years by various methods^[^ ^155^ ^]^ and investigated for SC fabrication.^[^ ^170^, ^171^, ^172^ ^]^

##### Nickel Hydroxide [Ni(OH)_2_]

Ni(OH)_2_ is also an attractive electrode material because of its high theoretical capacity, superior redox behavior, and potential applications in alkaline batteries and SCs. Its main reaction mechanism as positive electrode material for SCs is shown as follows:

(19) $$\mathrm{Ni}{\left(\mathrm{OH}\right)}_{2}+{\mathrm{OH}}^{-}⇌\mathrm{NiOOH}+H_{2}O+e^{-}$$

However, Ni(OH)_2_ usually suffers from poor stability, lower conductivity, and large volume changes during the charge/discharge processes. Thus, composites with high surface‐area conductive materials such as CNTs, activated carbon, graphene, show remarkably enhanced electrochemical performance due to improved electrical conductivity of the composites, and the shortening of the electron and ion diffusion pathways.^[^ ^173^, ^174^, ^175^, ^176^ ^]^

Cobalt oxide (Co_3_O_4_) is generally considered one of the best candidates for electrode material in the field of SCs owing to its superior reversible redox behavior, excellent cycle stability, large surface area, and outstanding corrosion stability.^[^ ^177^, ^178^, ^179^ ^]^ The redox reactions in alkaline electrolyte solution can be expressed as follows:

(20) $${\mathrm{Co}}_{3}O_{4}+OH^{-}+H_{2}O⇌3\mathrm{CoOOH}+e^{-}$$

(21) $$\mathrm{CoOOH}+{\mathrm{OH}}^{-}⇌{\mathrm{CoO}}_{2}+H_{2}O+e^{-}$$

Due to its layered structure with a large interlayer spacing, Cobalt hydroxide [Co(OH)_2_] provides a large surface area and a high ion insertion/extraction rate and offers a great potential to become a high‐performance electrode material^[^ ^155^ ^]^ and explored for SC studies.^[^ ^110^, ^111^ ^]^ The pseudofaradaic reaction at a low potential of Co(OH)_2_ and the faradaic reaction at a higher potential can be expressed as follows:

(22) $$\mathrm{Co}{\left(\mathrm{OH}\right)}_{2}+{\mathrm{OH}}^{-}⇌\mathrm{CoOOH}+H_{2}O+e^{-}$$

(23) $$\mathrm{CoOOH}+{\mathrm{OH}}^{-}⇌{\mathrm{CoO}}_{2}+H_{2}O+e^{-}$$

Among iron oxides, Fe_3_O_4_ is one of the main and naturally abundant pseudocapacitive materials with a reasonable metallic electrical conductivity (≈10^2^–10^3^ S cm^−1^). However, the low specific capacitance limits its practical applications. TiO_2_ is also considered a very important material for energy storage systems because of its good intercalation/ deintercalation behavior of metal ions (such as Li+ and Na+) without the formation of solid electrolyte interface by‐products and electrode collapse caused by volume changes. These characteristics contribute to its high‐power capacity and long lifespan. Non‐toxicity, chemical stability, photocatalytic activity, and low cost make it a promising semiconductor.^[^ ^180^ ^]^ In terms of properties, TiO_2_ is suitable for use as negative electrode material in organic electrolytes for hybrid SCs. SnO_2_ is another alternative electrode material to be used in SCs. But compared to other metal oxides, it has a much lower specific capacitance. The several oxidation states of vanadium in V_2_O_5_ result in both surface and bulk redox reactions. Therefore, it has been studied for its potential application in energy storage devices. V_2_O_5_ has a higher capacitance in KCl electrolyte than in any other electrolyte solutions. **Table** **6** compares the basic types of electrode materials for the fabrication of SCs.

**Table 6:** Comparison of various supercapacitor materials.^[^ ^129^ ^]^

Properties	Carbonaceous material	Metal oxides	Conductive polymers
Non‐faradic capacitance	Very high	Medium	Medium
Faradic capacitance	Very low	Very high	Very high
Conductivity	Very high	Low	Very high
Energy density	Low	High	Medium
Power density	High	Low	Medium
Cost	Medium	High	Medium
Chemical stability	Very high	Low	High
Cycle life	Very high	Medium	Medium
Ease of fabrication	Medium	Low	High
Flexibility	Medium	Very low	High

#### 3.2.4 2D Materials

Since the discovery of graphene, 2D materials (Figure 7d) such as hexagonal boron nitride (h‐BN), transition metal chalcogenides (TMDs)‐ Molybdenum disulfide (MoS_2_), Tungsten selenide (WSe_2_), transition metal carbides/nitrides (i.e., MXenes‐ Ti_2_C) and 2D metal‐organic frameworks (MOFs) also attracted tremendous research attention due to their extraordinary properties including large surface area, good electronic conductivity, excellent electrochemical properties, and good chemical, electrochemical, and thermal stability, since these properties are promising for batteries and SCs.^[^ ^181^, ^182^, ^183^ ^]^ 2D materials are generally defined as materials with infinite crystalline extensions along two dimensions and one crystalline dimension with few or single atomic layers thickness. Such materials are derived from most classes of known layered materials and possess strong in‐plane bonds within the layers and only weak interactions between neighboring layers.^[^ ^184^ ^]^ However poor cyclic stability, large structural changes during metal‐ion insertion/extraction, as well as higher manufacturing cost are the major challenges for 2D materials which require further improvements to find their applications in commercial batteries and SCs.^[^ ^185^ ^]^

2D hexagonal boron nitride (2D‐hBN), an isomorph of graphene with a very similar layered structure,^[^ ^186^ ^]^ is uniquely featured by its exotic opto‐electrical properties together with mechanical robustness, thermal stability, and chemical inertness. 2D‐hBN is an insulator itself but can well be tuned by several strategies in terms of properties and functionalities, such as doping, substitution, functionalization, and hybridization, making 2D‐hBN a truly versatile type of functional material for a wide range of applications. More importantly, both theoretical and experimental results show that the BN–noble metal interface can also improve electrocatalytic activity. Recent studies have also shown that it has the ability to adsorb polysulfides and Li ions, which is a greatly desired property for improving the performance of Li–S and solid‐state batteries. Thus, BN‐based nanomaterials have huge potential in the field of electrochemical energy storage and conversion.^[^ ^187^ ^]^ Additionally, it is considered as one of the most promising materials, which is able to integrate with other 2D materials, including graphene and TMDCs for the next generation microelectronic and other technologies,^[^ ^188^ ^]^ as well as SC electrodes.^[^ ^189^, ^190^, ^191^, ^192^ ^]^

MoS_2_, another exciting 2D material, has been investigated to a lesser extent but is gaining increased interest recently for integration into electronic devices due to their grapheme‐like properties. Exfoliated MoS_2_ possesses high catalytic activity which makes it an efficient hydrogen evolution catalyst as well as a useful energy storage material for the use in lithium and sodium‐ion batteries. In addition to conventional synthesizing processes such as micromechanical peeling or chemical vapor deposition, currently, MoS_2_ is being synthesized by ultrasonic treatment similar to graphene. It creates large volumes of monolayer and few‐layer flakes that can then be deposited onto a substrate or formed into films.^[^ ^193^ ^]^ The favorable electrochemical properties are mainly a result of the hydrophilicity and high electrical conductivity, as well as the ability of the exfoliated layers to dynamically expand and intercalate various ions.^[^ ^194^ ^]^ Similar to h‐BN, MoS_2_ has been explored alone^[^ ^195^, ^196^ ^]^ or with other functional materials^[^ ^197^, ^198^, ^199^, ^200^ ^]^ for SC electrode fabrication.

MXene, a new family of 2D metal carbides, nitrides, and carbonitrides have gained much attention due to their attractive electrical and electrochemical properties such as hydrophilicity, conductivity, surface area, topological structure, rich surface chemistry, tunable terminations, excellent processability, etc.^[^ ^201^, ^202^, ^203^ ^]^ The term MXenes with a formula of M*_n_*_+1_X*_n_*, are named after other 2D analog materials silicene, graphene, phosphorene, and so on, synthesized by extracting an atomic layer from ternary MAX (M*_n+_*_1_AX*_n_)* ceramics, where M = early transition metal elements (Ti, Zr, Mo, Nb, V, Mn, Sc, Hf, W, and so on), A = group 13 or 14 (Si, Al, Ga, and so on), X = C or/and N. Due to their unique intrinsic physical/chemical properties, 2D MXenes materials have thoroughly been investigated and can be used in various research fields, including ceramics, conductive polymer, energy storage, sensors, water purification, catalysis, thermoelectric conversion, photothermal conversion, solar cell, biomedicine, and microwave absorption and shielding.^[^ ^204^, ^205^ ^]^ Moreover, the improved coupling and hybridization of MXene with other materials at the nano‐scale make it one of the most intriguing materials for wearable applications.^[^ ^206^, ^207^ ^]^

### 3.3 Electrolytes for Supercapacitors

Electrolytes are vital constituents of SCs, as their physical and chemical properties play an important role to obtain desired performances in terms of capacitance, power density, rate performance, cyclability, and safety.^[^ ^208^ ^]^ For the SC performance, the type, composition, and concentration of the electrolyte are as important as the electrode materials.^[^ ^209^ ^]^ An optimized electrolyte concentration is always desired, as the ion transport within the electrode layers becomes easier at high electrolyte concentration, inducing an effective build‐up for the double layer. If the concentration becomes too high, the ion activity is reduced due to less water hydration, resulting in decreased ion mobility. A good electrolyte offers a wide voltage window, high electrochemical stability, high ionic concentration and conductivity, low viscosity, and low toxicity. However, a proper cell design should also consider the key electrolyte parameters, such as: i) sufficiently high ion conductivity, ii) electrochemical stability on the anode and cathode surfaces, iii) good wetting in contact with electrode materials, iv) suitable thermal properties, v) adequate cost, and vi) adequate mechanical properties.^[^ ^210^ ^]^ Common electrolytes can be classified into three types: aqueous, organic liquid, and ionic liquid (IL).^[^ ^211^ ^]^ Due to offering safer and more packageable construction, providing more design freedom, larger operable temperature range, and electrochemical stability, polymer‐based electrolytes have also garnered significant attention for SC fabrication.

#### 3.3.1 Aqueous Electrolytes

The aqueous electrolytes can be categorized into three types: acidic solution (such as H_2_SO_4_ solution), alkaline solution (such as KOH solution), and neutral solution (such as Li_2_SO_4_, Na_2_SO_4_, or KCl solution). Due to the high ionic conductivity of acidic aqueous electrolytes, SC electrode materials show better performance in comparison with neutral aqueous electrolytes.^[^ ^212^ ^]^ They get dissolved in water, providing high ionic conductivity, and low internal resistance compared with organic electrolytes. Therefore, SCs with an aqueous electrolyte solution may possess a higher capacitance and power than capacitors containing organic electrolytes. The maximum working voltage of aqueous electrolyte is limited to 1.23 V owing to the thermodynamic decomposition of water. In addition, they can be prepared and employed without much tight control of the production process, whereas organic electrolytes require strict preparation procedures to obtain pure electrolytes. Commonly used aqueous electrolytes are inorganic salts (e.g., LiCl, NaCl) (for Li‐ion & SCs), alkali (e.g., KOH) and inorganic acid (e.g., H_2_SO_4_) in water (for SCs).

#### 3.3.2 Organic Electrolytes

Organic electrolytes allow a much wider voltage window of about 3.5 V resulting in a large advantage with respect to higher energy density. Among the organic electrolytes, propylene carbonate (PC) is the most commonly used solvent, because of their environmentally friendly nature and wide voltage window with good conductivity. The complex purification and preparation procedures may cause safety problems due to the flammability and toxicity of some of the organic solvents. Furthermore, their low conductivity could lead to lower power and smaller capacitance. Several combinations of organic solvents and lithium salts have been examined as electrolytes for ambient‐temperature, rechargeable lithium batteries.^[^ ^213^ ^]^ Inorganic or organic salts, for example, Lithium hexafluorophosphate (LiPF_6_); Tetraethylammonium tetrafluoroborate (TEABF_4_) in organic solvents (carbonates, ethers, sulfones, etc., some of which may be fluorinated) are the common examples of organic electrolytes for SCs.

#### 3.3.3 Ionic Liquids

ILs are salts having uncommonly low melting points, below 100 °C, which are usually liquid at room temperature.^[^ ^214^ ^]^ They are inherent and competitive electrolytes due to their ability to overcome many disadvantages of the conventional aqueous and organic electrolytes,^[^ ^215^ ^]^ for instance volatility, high thermal and chemical stability, extensive electrochemical stability window between 2 and 6 V, low flammability, nontoxicity and the wide variety of cation and anion combinations. They are composed entirely of ions, solvent‐free, and liquids at room temperature, making them attractive “green electrolytes”. They are usually highly viscous liquids with low ionic conductivity at ambient temperatures, seriously influencing their electrochemical performance. Salts in ILs (for Li‐ion batteries) or pure ILs (for SCs); organic cations (e.g., imidazolium, pyridinium, pyrrolidinium, etc.) with inorganic or organic anions (e.g., H_2_SO_4_) in water (for SCs) are commonly used ILs.

#### 3.3.4 Polymer Electrolytes

In order to meet the safety (for instance the leakage, flammability, and toxicity of organic electrolytes), flexibility, and multi‐functionality requirements for advanced energy‐storage devices (ESDs), polymer electrolytes are considered to be the best candidate to replace liquid electrolytes due to their wide electrochemical window, good thermal stability and less risk with electrolyte solution leakage.^[^ ^216^ ^]^

##### Dry Solid Polymer Electrolytes (Polymer‐Salt Complex Electrolytes)

A solid polymer electrolyte is prepared by dissolving inorganic salts into a polar functional polymer, which forms a solid electrolyte with ion‐conducting after drying. With interactions between metal ions and polar groups inside the polymers, electrostatic forces are generated due to the formation of coordinating bonds. Since the coordination of cations to align along the polymer chain is weak, after applying an electric field the cations in the electrolyte may migrate from one coordinated site to another. Various polymers are used to form dry and solid polymer electrolytes including polycarbonate (PC), poly (methyl methacrylate) (PMMA), poly (ethylene oxide) (PEO), and poly(vinyl alcohol) (PVA). For metal ions, various soluble compounds, such as salts containing lithium (Li), sodium (Na), and potassium (K) can be used. Nevertheless, the room ionic conductivities of SPEs (≈10^−8^ –10^−5^ S cm^−1^) are lower than the required conductivity of 10^−3^ S cm^−1^ in actual application, limiting their wide practical applications.^[^ ^216^ ^]^

##### Gel Polymer Electrolytes

The gel polymer electrolytes possess a higher ionic conductivity at room temperature. It has attracted research attention because of the combination of the advantages of high ionic conductivity of liquid‐based electrolytes, and the high stability of solid‐based electrolytes. This combination superiority is embodied in high ionic conductivity and good interfacial properties from the liquid phase as well as good mechanical properties from the solid component. They are safer to use compared to liquid‐based electrolytes. The majority of GPEs exhibit outstanding ionic conductivity in the order of 10^−3^ S cm^−1^ at ambient temperature, which can boost the electrochemical performance of the cells involving GPEs. Consequently, GPEs have become one of the most desirable alternatives for the fabrication of advanced ESDs with enhanced safety and flexibility.^[^ ^38^, ^217^ ^]^

##### Plasticized Polymer Electrolytes

A host polymer with lower molecular weight like poly(ethylene glycol) (PEG), PC, and ethylene carbonate is used to produce plasticized polymer electrolytes. The rigidity of the polymer structure is decreased with a change in their mechanical and thermomechanical properties. The glass transition temperature of the particular polymer electrolyte system is also decreased. The increase of salt dissociation capability and the reduction of crystallinity results in the enhancement of charge carrier transportation.^[^ ^38^ ^]^ Polymer electrolytes are found to exhibit higher ionic conductivity at higher plasticizer concentration at the cost of their mechanical stability.^[^ ^218^ ^]^

##### Composite Polymer Electrolytes

**Figure 8:**
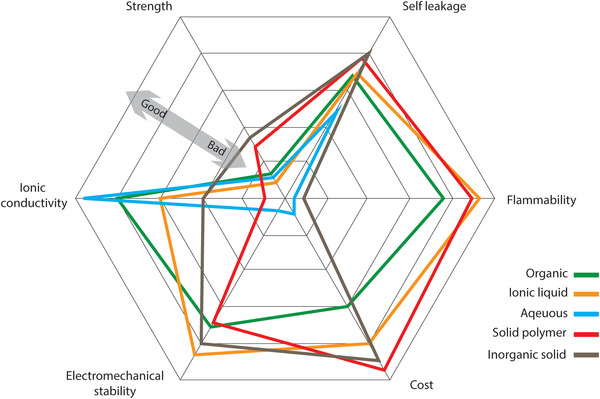
Comparison of the performance of several electrolyte types.

The addition of inorganic fillers in polymer electrolytes increases the mechanical strength and interfacial stability of the resulting electrolytes, providing a new branch of polymer electrolytes (Pes) which are known as composite polymer electrolytes (CPEs).^[^ ^219^ ^]^ By doping different types and amounts of high dielectric constant fillers, especially inorganic inert fillers into the polymer matrix, the electrical properties of polymer electrolytes can be improved. Ceramic materials are one of the most used inorganic dopants. They are fragile and have low dielectric strength. By combining such inorganic dopants with polymers, the new composite electrolytic material can be produced for higher relative permittivity. Since these composite electrolytes consist of ceramic particles, they can be regarded as heterogeneously disordered systems, with electrical properties highly dependent on the relative permittivity and conductivity of the dopants. Moreover, electrical performances of these composite electrolytic materials are affected by the size, shape, and volume fraction of the dopants.^[^ ^38^ ^]^ **Figure** **8** summarizes some of the key features of major electrolyte families to compare their advantages and disadvantages.

## 4 Manufacturing of Conductive Electrodes

Several technologies can be utilized for the manufacturing of conductive electrodes. Spinning, coating, and printing of active materials with/on textiles are the key manufacturing techniques for such electrodes. In situ growth of active materials on/ in the substrate is another way of such manufacturing.

### 4.1 Coating of Active Materials on Substrate

The conventional textile materials such as cotton, polyester, and nylon are electrically nonconductive. Therefore, electrical conductivity must be introduced to such textiles to prepare a textile‐based energy storage device. The coating of a layer of electrically conducting material onto non‐conductive textiles can impart electronic capabilities in a facile manner. Materials such as ICPs, conductive polymer composites, metals, and carbon based materials (e.g., CNTs, carbon nano powders, graphene, etc.) have been used to achieve this.^[^ ^220^ ^]^ The commonly used coating techniques that have been used to deposit such materials on textiles are dip coating, doctor‐blade coating, wrapping, physical vapor deposition (VPD), and chemical vapor deposition (CVD).^[^ ^32^, ^38^ ^]^

**Figure 9:**
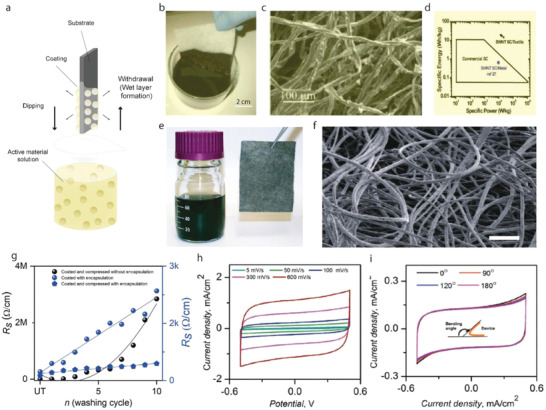
a) Schematic diagram of dip coating technique. b) Conductive textiles fabricated by dipping textile into an aqueous SWNT ink followed by drying in oven at 120 °C for 10 min. c) SEM image of coated cotton reveals the macroporous structure of the cotton sheet coated with SWNTs on the cotton fiber surface. d) Ragone plot of commercial SCs, SWNT SC on metal substrates, and SWNT SC on porous conductors including all the weight. Reproduced with permission.^[^ ^223^ ^]^ Copyright 2010, American Chemical Society. e) Photograph of a stable, solution‐exfoliated graphene ink suspension prepared by ultrasonication of the graphite powder in a water sodium cholate solution, and a 6 cm X 8 cm graphene‐coated conductive textile sheet (polyester fabrics). f) SEM image of a sheet of graphene‐coated textile after 60 min MnO_2_ electrodeposition showing large‐scale, uniform deposition of MnO_2_ nanomaterials achieved on almost entire fabric fiber surfaces, Scale bar: 200 µm. Reproduced with permission.^[^ ^224^ ^]^ Copyright 2011, American Chemical Society. g) The change in resistance with the number of washing cycles of G‐coated compressed (with encapsulation) poly‐cotton fabric, G‐coated only (with encapsulation) poly‐cotton fabric, and G‐coated compressed (without encapsulation) poly‐cotton fabric. h) Cyclic voltammograms (CV) recorded for the supercapacitor device at different scan rates i) CV curves for the ASC device at different bending angles. Reproduced with permission.^[^ ^225^ ^]^ Copyright 2020, Wiley‐VCH.

Dip Coating, also termed as impregnation or saturation coating, is the simplest process of creating a uniform thin layer of conductive materials on a substrate. In such a technique, textile substrates are dipped into a bath containing coating materials in liquid form, **Figure** **9**a. The viscosity of the coating liquid is usually very low to enable it to run off while the substrate leaves the coating liquid. A pair of nip rollers are often placed to remove the excessive liquor from coated surface, providing a homogeneous liquid film on the substrate.^[^ ^221^ ^]^ After drying, the volatile solvents are eliminated, followed by possible chemical reactions, resulting in a thin coated film.^[^ ^222^ ^]^ Dip coating offers a simple, low‐cost, reliable, and reproducible method, which is extensively effective for research purposes. However, the inconsistent quality of such coatings makes them unsuitable for industrial‐scale application.^[^ ^139^ ^]^ Hu et al. reported a simple dipping and drying of SWNTs ink on textiles to produce highly conductive textiles with electrical conductivity of 125 S cm^−1^ and sheet resistance < 1 Ω sq^−1^. SCs made from such conductive textiles showed high areal capacitance, up to 0.48F cm^−2^, and specific energy as high as 20 Wh kg^−1^ at a specific power of 10 kW kg^−1^, Figure 9(b–d).^[^ ^223^ ^]^ The same research group later demonstrated the coating of polyester fabric with solution‐exfoliated graphene nanosheets and further electrodeposition of MnO_2_ nanomaterials, yielding high specific capacitance up to 315 F g^−1^. They also successfully fabricated asymmetric electrochemical capacitors with graphene/MnO_2_‐textile as the positive electrode and SWNTs‐textile as the negative electrode with aqueous Na_2_SO_4_ electrolyte, exhibiting promising characteristics with a maximum power density of 110 kW kg^−1^, an energy density of 12.5 Wh kg^−1^, and excellent cycling performance with ≈95% capacitance retention over 5000 cycles, Figure 9(e,f).^[^ ^224^ ^]^ Dip coating is a simple and scalable process, however, the loading of active materials depends on the surface properties as well as the deposition position of the textile substrate. The repeated dipping‐drying cycles are usually employed to achieve sufficient material loading which lowers the efficiency of fabrication process. Padding is a modified version of dip coating, used for continuous treatment of textiles for various chemical treatments and finishes. Textile substrate after being impregnated with the solution is squeezed through nip rollers.^[^ ^8^ ^]^ In a previous study,^[^ ^225^ ^]^ we obtained the lowest sheet resistances (≈11.9 Ω sq^−1^) reported on graphene e‐textiles, through a simple and scalable pad−dry−cure method with subsequent roller compression and a fine encapsulation of graphene flakes. The grapheme‐coated textiles were highly conductive even after 10 home laundry washing cycles with extremely high flexibility, bendability, and compressibility as it shows the repeatable response in both forward and backward directions before and after home laundry washing cycles. The potential applications of such conductive textiles were demonstrated as ultra‐flexible SC and skin‐mounted strain sensors, Figure 9(g,i).

Doctor blade coating, also called knife coating or blade coating or tape casting, is another widely used technique for producing thin films on surfaces with large areas. The process involves a constant relative movement between a blade over the substrate or a substrate underneath the blade, resulting in a spread of the coating material on the substrate to form a thin film on the substrate upon drying. The operating speed of such a technique can reach up to several meters per minute, and coat substrate with a very wide range of wet film thicknesses ranging from 20 to several hundred microns.^[^ ^226^ ^]^ This process can create thin uniform films over large surface areas quickly and efficiently, though cannot offer nanoscale uniformity or extreme thin film. Nevertheless, the scalability, versatility, and simplicity of this technique make it perfect for industrial applications. In comparison to dip coating, doctor blade technique allows much more precise and uniform control over the coating amount of active materials in a continuous process.^[^ ^33^ ^]^ Though few literatures are available on fabric or thin‐film‐based lithium‐ion batteries fabricated using doctor blade coating,^[^ ^227^ ^]^ the fabrication of SCs using such technique is rare.

**Figure 10:**
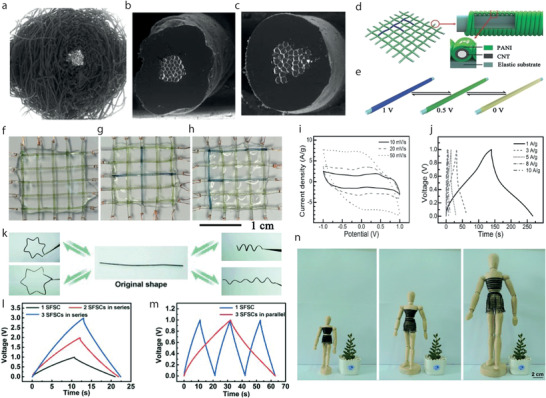
SEM pictures of a) shieldex conductive yarn wrapped with PP staple fiber, b) Melt coated single yarn, and c) Melt coated plied yarn. Reproduced with permission.^[^ ^228^ ^]^ Copyright 2012, Taylor & Francis. d) Schematic illustration of the structure of the electrochromic, wearable fiber‐shaped supercapacitor. e) Positive electrode demonstrates rapid and reversible chromatic transitions between blue, green, and light yellow under different working states. f) An energy storage textile woven from electrochromic fiber‐shaped supercapacitors during the charge–discharge process. g,h) Electrochromic fiber‐shaped supercapacitors that have been designed and woven to display the signs “+” and “F”, respectively. i) Cyclic voltammograms at various scan rates. j) Galvanostatic charge–discharge profiles at different current densities. Reproduced with permission.^[^ ^229^ ^]^ Copyright 2014, Wiley‐VCH. k) Photographs of an SFSC transformed into different shapes and sizes. l,m) Galvanostatic charging and discharging curves of SFSCs arranged in series and parallel, respectively. The galvanostatic charging and discharging tests were performed at a current density of 0.5 A g^−1^. n) Photographs of the same smart clothes woven from SFSCs that were “frozen” into different shapes and sizes. Reproduced with permission.^[^ ^230^ ^]^ Copyright 2015, Wiley‐VCH.

Conductive materials (fibers, yarns, or fabrics) are integrated with textile fabrics by various methods such as weaving, knitting, braiding, or embroidery process. To protect the conductive material from being rubbed away from the textiles during a washing cycle or to avoid fraying or short circuits between neighboring materials while used underwater, wrapping might be an effective solution.^[^ ^228^ ^]^ Alagirusamy et al.^[^ ^228^ ^]^ reported their attempt to protect single and plied silver‐coated polyamide yarns by wrapping polypropylene (PP) staple fibers around the silver‐coated polyamide yarns through friction spinning and melting of PP sheath fibers in an oven, **Figure** **10**(a–c).

Chen et al.^[^ ^231^ ^]^ fabricated a stretchable wire‐shaped SC by twisting two CNTs thin film wrapped elastic wires, pre‐coated with poly(vinyl alcohol)/H_3_PO_4_ hydrogel, as the electrolyte and separator. It exhibited an extremely high elasticity of up to 350% strain and a high device capacitance of up to 30.7 Fg^−1^. This wire‐shaped structure facilitated the integration of multiple SCs into a single wire device to meet specific energy and power needs for various potential applications.^[^ ^231^ ^]^ An electrochromic fiber‐shaped SC was developed by Chen et al.,^[^ ^229^ ^]^ by winding aligned CNT/polyaniline composite sheets on an elastic rubber fiber. It exhibited rapid and reversible chromatic transitions under different working states, which can be directly observed by the naked eye, Figure 10(d–j). At 70% wt.‐% of PANI, the specific capacitance was 255.5 F g^−1^ and the power density was 1494 W kg^−1^ at 10 A g^−1^.^[^ ^229^ ^]^ A shape‐memory fiber‐shaped SC was also developed by Deng et al.,^[^ ^230^ ^]^ via winding aligned CNT sheets on a shape‐memory polyurethane (PU) substrate. The length and volumetric specific capacitances were 0.269 mF cm^−1^ and 42.3 mF cm^−3^, respectively, which were well‐maintained during deformation, both at the deformed state and after the recovery, Figure 10(k–n). A solid‐state SC was prepared by Choi et al.^[^ ^232^ ^]^ via imparting twist to coil a nylon sewing thread helically wrapped with a CNT sheet and then electrochemically depositing pseudocapacitive MnO_2_ nanofibers. The maximum linear and areal capacitances, areal energy storage, and power densities were found as high as 5.4 mF cm^−1^, 40.9 mF cm^−2^, 2.6 µWh cm^−2^, and 66.9 µW cm^−2^ respectively.

### 4.2 Printing of Active Material on Substrate

The process described as printing involves the controlled deposition of a material, either for decorative or functional purposes, onto a substrate in such a manner that a pre‐defined pattern is produced. Other deposition processes, such as painting or spraying, have much in common, but printing is further defined because the process can rapidly produce identical multiples of the original. There are three basic methods of printing: positive contact, negative contact, and non‐contact printing. The first two methods are described as contact printing since the substrate is touched by the print master. The positive contact type resembles the principle of stamping, examples include printing presses and woodcuts. Gravure or screen printing are examples of negative contact type printing. In non‐contact printing, the printer does not contact the substrate. The most common example of non‐contact printing is inkjet printing (IJP), where ink droplets are ejected from a nozzle, and deposited on a substrate.^[^ ^233^ ^]^

Screen printing is a stencil process, in which the printing ink is transferred to the substrate through a stencil supported by a fine fabric mesh of either silk, synthetic fibers, or metal threads stretched tightly on a frame.^[^ ^234^ ^]^ The squeegee or the blade press the ink, which is most often a viscous paste, through the open parts of the mesh. When the print paste is moved over the mesh from one side to the other, it creates the final printed design on the substrate.^[^ ^235^ ^]^ Basically, this is a selective transfer process of ink through the open areas of the unmasked portions of a screen. The masking of the screen is accomplished by the transfer of a photographically produced image from its temporary film base support to the screen.^[^ ^236^ ^]^ The versatility of print substrates is one of the biggest advantages of screen printing, including paper, paperboard, polymer materials, textiles, wood, metal, ceramics, glass, and leather. The wide variety of polymer substrates requires different types of inks. Printing inks must be selected accordingly to the type and surface characteristics of printing substrates. A sharp edge of printed images requires inks with higher viscosity for screen printing than other printing techniques.^[^ ^237^ ^]^

**Figure 11:**
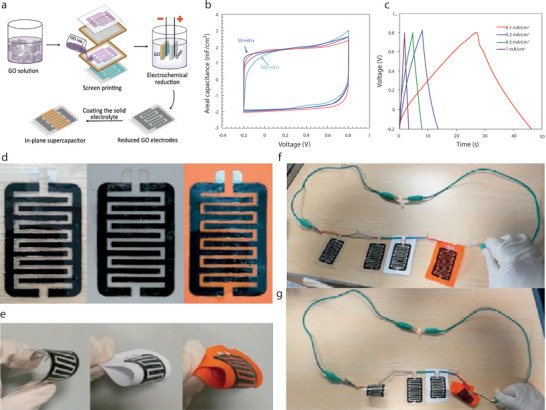
a) Schematic representation of the printed in‐plane supercapacitor fabrication process. Electrochemical characterization of printed graphene on textile. b) CV at different scan rates and c) charge/discharge curves at different current densities. Reproduced with Permission.^[^ ^239^ ^]^ Copyright 2017, IOP Publishing Ltd. d) Images of all printed solid‐state flexible SC devices on PET, paper, and textile substrates, e) images of these SCs after bending, f) image of these SCs in series lighting up a yellow LED, and g) images of the same after bending these SCs. Reproduced with permission.^[^ ^240^ ^]^ Copyright 2017, Elsevier B.V.

Jost et al.,^[^ ^238^ ^]^ investigated traditional screen printing of porous carbon materials on woven cotton and polyester fabric for fabricating flexible and lightweight SC electrodes as a possible energy source for smart garments. A high gravimetric capacitance (85 Fg^−1^) and areal capacitance (≈0.43 Fcm^−2^) on both cotton lawn and polyester microfiber were obtained. In another study, Abdelkader et al.^[^ ^239^ ^]^ reported a solid‐state flexible textiles SC device, produced via screen printing of GO ink on textiles. After the in situ reduction of GO, the printed electrodes exhibited excellent mechanical stability and flexibility, as well as outstanding cyclic stability over 10 000 cycles, which are necessary for wearable applications, **Figure** **11**(a–c). Lu et al.^[^ ^240^ ^]^ synthesized FeOOH/MnO_2_ composites which were screen‐printed as SC electrodes on different substrates, including PET, paper, and textiles. The all‐printed solid‐state flexible SC device exhibited high area‐specific capacitance of 5.7 mF cm^−2^ with 80% retention up to 2000 charge‐discharge cycles, and high mechanical flexibility. Additionally, they demonstrated printed SCs on different substrates, which are capable of lighting up a 1.9 V yellow light emitting diode (LED), even after bending and stretching, Figure 11(d–g).

**Figure 12:**
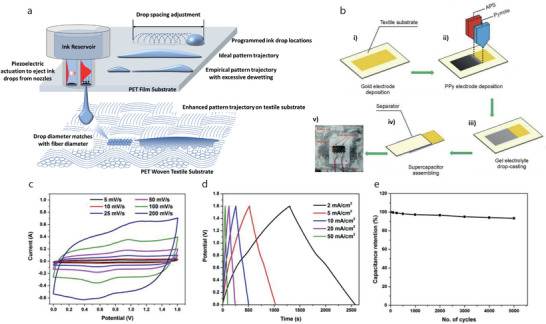
a) Schematic drawing of the inkjet process and ink spreading behavior on the film and textile substrates. Reproduced with permission.^[^ ^244^ ^]^ Copyright 2021, American Chemical Society. b) Detailed steps of fabrication of inkjet‐printed textile supercapacitor and printed samples. Reproduced with permission.^[^ ^254^ ^]^ Copyright 2021, The Authors. c) CV curves of MnO_2_–NiCo_2_O_4_//rGO asymmetric device at different scan rates. d) GCD profiles of the MnO_2_–NiCo_2_O_4_//rGO asymmetric device at various current densities and e) Capacitance retention of the device with the different number of charge–discharge cycles. Reproduced with permission.^[^ ^255^ ^]^ Copyright 2020, The Authors.

IJP is a digital technique, which can be used for printing functional materials with specific electrical, chemical, biological, optical, or structural functionalities. IJP has gained significant research interest due to its wide range of applications for different processes and purposes, from the batch coding of soft drink cans to smart e‐textiles.^[^ ^241^, ^242^ ^]^ The main advantages of IJP technology include digital and additive patterning, reduction in material waste, and compatibility with a variety of substrates with different degrees of mechanical flexibility and form factor.^[^ ^243^ ^]^ IJP forms nano to micron scale film through a one‐step printing process that benefits from downsizing the device thickness and increasing the uniformity of the coated area in an economical way. High‐performance devices can be produced by printing and stacking the functional inks in desired locations,^[^ ^244^ ^]^ **Figure** **12**a. IJP allows the deposition of tiny droplets onto the substrate without depending on the high‐speed operation of mechanical printing elements. The nozzle size for such printers is typically ≈20–30 µm and ink droplets can be as small as 1.0 pL to achieve high print resolution (dots per inch).^[^ ^245^, ^246^ ^]^ In addition to 2D prints, IJP can print layers of “structural” fluids that harden to form 3D structures. In spite of all these benefits, print speed, higher cost, printed film uniformity, and fluids' jet‐ability as ink, are still points of concern for inkjet printers.^[^ ^247^ ^]^

There are two distinct modes of IJP: Continuous inkjet (CIJ), suitable for industrial scale and mass‐production, and Drop‐on‐demand (DOD), used for small‐volume and prototype sample production.^[^ ^248^ ^]^ The high accuracy and small droplet size of DOD inkjet printers are the key advantages for the direct patterning of functional materials.^[^ ^249^, ^250^ ^]^ There are several parameters of ink such as viscosity, surface tension, density and size are important for successful IJP of a fluid. The spreading behavior of the ink is determined by the Ohnesorge number (Oh). The inverse of the Ohnesorge number is used to determine the printability of DOD inkjet inks. This is known as the Z number.^[^ ^251^ ^]^ The printability,

(24) $$Z=\frac{1}{\mathrm{Oh}}=\frac{\sqrt{\left(\gamma \rho a\right)}}{\eta }$$

Where, *η* represents dynamic viscosity, *γ* is surface tension, *ρ* is density, and *a* is the characteristic length (usually the diameter of the print head's nozzle). Several research groups studied the printability of inkjet inks.^[^ ^251^, ^252^ ^]^ Moon et al.^[^ ^253^ ^]^ summarized Z values for inkjet ink to be in between 4 and 14 to be ideally printable. Considering the drop generation, drop flight and drop impact, the optimal value of the physical conditions for a robust DOD IJP are typically with surface tension lying in the range of 20–50 mN m^−1^ and viscosity within the range of 2–20 mPa s. to achieve high‐resolution print on the desired trajectory, where narrower and specified range would be more applicable for specific print heads.^[^ ^110^ ^]^

For e‐textile fabrication, IJP offers a number of advantages over conventional manufacturing techniques including the ability to deposit controlled quantities of materials at precise locations of the fabric, combined with a reduction in both material waste and water utilization.^[^ ^256^ ^]^ However, the key challenge with IJP of e‐textiles is the ability to achieve a uniform and continuous highly conductive electrical tracks on a rough and porous textile substrate using low viscosity inkjet inks. To solve this, we developed a novel surface pre‐treatment that was inkjet‐printed onto rough and porous textiles before IJP of electrically conductive graphene‐based inks for wearable e‐textiles applications.^[^ ^14^ ^]^ Later, we formulated graphene‐Ag composite inks for IJP onto surface pre‐treated cotton fabrics, to enable all‐inkjet‐printed highly conductive e‐textiles with a sheet resistance in the range of ≈0.08–4.74 Ω sq^−1^.^[^ ^256^ ^]^ Stempien et al.^[^ ^254^ ^]^ propose an IJP method to prepare PPy layers on textile fabrics using direct freezing of inks under varying temperatures up to −16 °C. The as‐coated PPy layers on PP textile substrates were further assembled as the electrodes in a symmetric all‐solid‐state SC device, Figure 12b. The electrochemical results demonstrate that the symmetric SC device made with the PPy prepared at −12 °C, showed the highest specific capacitance of 72.3 F g^−1^ at a current density of 0.6 A g^−1^, and delivers an energy density of 6.12 Wh kg^−1^ with a corresponding power density of 139 W kg^−1^. Sundriyal et al.^[^ ^257^ ^]^ reported an inkjet‐printed, solid‐state, planar, and asymmetric micro‐supercapacitor (PAµSC) deskjet printed on cellulose paper substrate. They digitally designed interdigitated electrode patterns and printed them on paper with rGO ink to construct a conducting matrix. The negative electrode was printed using activated carbon–Bi_2_O_3_ ink and the positive electrode was printed with rGO‐MnO_2_ ink. After that, they demonstrated bamboo fabric as a sustainable substrate for developing SC devices with a replicable IJP process.^[^ ^255^ ^]^ Different metal oxide inks such as MnO_2_–NiCo_2_O_4_ were used as a positive and rGO as a negative electrode. With LiCl/PVA gel electrolyte, the textile‐based MnO_2_–NiCo_2_O_4_//rGO asymmetric SC displayed excellent electrochemical performance with an overall high areal capacitance of 2.12 F cm^−2^ (1766 F g^−1^) at a current density of 2 mA cm^−2^, excellent energy density of 37.8 mW cm^−3^, a maximum power density of 2678.4 mW cm^−3^ and good cycle life, Figure 12(c–e).

### 4.3 Spinning Technology

Spinning of polymer fibers, is an interdisciplinary field applying the principles of engineering and material science toward the development of textile substitutes. Electrically conducting textile materials can be spun in the form of staple fiber or filament yarn based on intrinsic conductive polymers (ICPs). An extruded liquid polymer filament is continuously drawn and simultaneously solidified to form a continuous synthetic fiber in this process.^[^ ^258^ ^]^ This is based on a special extrusion process that uses spinneret (a nozzle‐type device) to form multiple continuous filaments or monofilaments. Available spinning technologies such as dry, wet, melt, or electrospinning can be exploited for this purpose. The polymer, needed to form fiber, is first converted into a spinnable solution. The solidification of the ejected solution is carried out by the removal of heat and/or solvent by contacting the liquid with a suitable moving fluid, either with a gas or a liquid.^[^ ^259^, ^260^ ^]^

**Figure 13:**
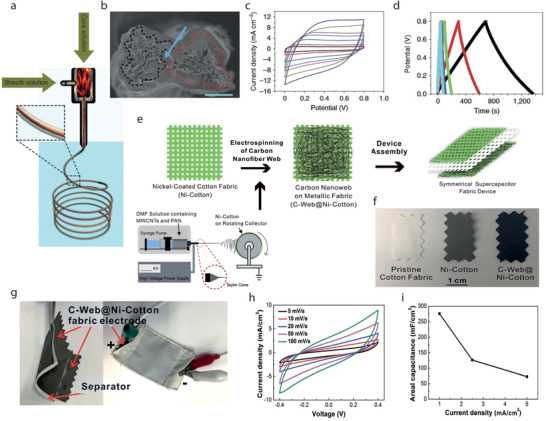
a) Schematic illustration showing the coaxial spinning process. Two‐ply YSCs and their electrochemical properties. b) SEM images of cross‐sectional view of a two‐ply YSC. The arrow area is PVA/H_3_PO_4_ electrolyte (scale bar: 50 µm) c) CV curves of RGO+CNT@CMC. d) GCD curves of RGO+CNT@CMC. Reproduced with permission.^[^ ^264^ ^]^ Copyright 2014, The Authors. e) Schematic illustration of the textile based SC fabrication process. The inset shows details of one‐step electrospinning setup. f) Photographs of the pristine cotton fabric, Ni‐coated cotton fabric (Ni–cotton), and CNF web‐coated Ni–cotton fabric (C‐web@Ni–cotton). g) Photographs of a large supercapacitor fabric (active area: 4 cm× 4 cm) enclosed with commercial nonconductive fabrics. h) CV curves of solid‐state C‐web@Ni–cotton supercapacitor fabric. i) Summary of the areal capacitance of the supercapacitor at different current densities. Reproduced with permission.^[^ ^267^ ^]^ Copyright 2016, The Royal Society of Chemistry.

The basic dry spinning process involves dissolving the polymer in organic solvents followed by blending with additives and filtering to produce a low viscosity polymer solution, called as “dope.” The dope is filtered, de‐aired and pre‐heated, and pumped through filters to achieve the right consistency. The dope is then extruded into a spinning tube where the solution is forced through the fine orifices of spinneret (or jet). The exiting jets of the polymer solution, when coming in contact with a stream of hot gas, vaporizes the solvent in the gas stream, increasing the polymer concentration in the filament and thus solidifying it without the need for further drying.^[^ ^261^ ^]^ This complex process makes dry spun fibers very expensive. Additionally, the poor solubility of most conductive polymers in organic solvents, makes them unsuitable for the production of conductive polymer filaments using such a technique. Wet spinning is another technique, which requires pumping of the polymer solution through the fine orifices of a spinneret into a coagulating bath and drawing off as continuous filaments by means of take‐up rollers. The bath removes the solvent from the as‐spun filaments so that they become solidified. They are collected together to form a continuous tow or rope.^[^ ^262^ ^]^ Wet spinning is slower than other spinning processes due to the mass transfer of the solvent and non‐solvent for fiber solidification. Zhang et al.,^[^ ^263^ ^]^ exploited the solubility properties of polyaniline (PANI) to blend with poly‐*ω*‐aminoundecanoyle (PA11) in concentrated sulfuric acid (c‐H_2_SO_4_) to form a spinning dope solution to spin conductive PANI / PA11 fibers by wet‐spinning technology. Kou et al.,^[^ ^264^ ^]^ proposed a coaxial wet‐spinning assembly to spin polyelectrolyte‐wrapped graphene/CNT core‐sheath fibers continuously, which were used directly as safe electrodes to assemble two‐ply yarn SCs, **Figure** **13**(a–d). The yarn SCs using liquid and solid electrolytes exhibited ultra‐high capacitances of 269 and 177 mF cm^−2^ and energy densities of 5.91 and 3.84mWh cm^−2^, respectively.

Melt spinning is one of the most popular methods for manufacturing synthetic fiber filaments. It requires no solvents, thus a simple and economical process. In melt‐spinning process, the polymer pellets or granules are fed into an extruder for melting by heating, and then the polymer melt is pumped through a spinneret under pressure. After extrusion, it is quenched with cold air which solidifies the molten mass to form filaments. Spinning is followed by a mechanical drawing to improve the degree of crystallinity, which contributes to improving physical, mechanical, and chemical properties.^[^ ^265^ ^]^ Kim et al.,^[^ ^266^ ^]^ melt‐mixed polyaniline emeraldine salt (PANI‐ES), PPy, and graphite with PP and low‐density polyethylene (LDPE) using a co‐rotating twin screw extruder. However, the electrical conductivity of conductive materials/PP monofilament obtained by melt spinning process was not found satisfactory, due to the problems of structural homogeneity and the aggregation of conductive materials.

Electrospinning is another fiber spinning process, which is used to produce ultrafine fibers by charging and ejecting a polymer melt or solution through a spinneret under a high‐voltage electric field and to solidify or coagulate to form filaments.^[^ ^268^ ^]^ This is a relatively simpler and cheaper spinning process, as well as a versatile method to produce continuous, long, and fine (in the range of nano to sub‐micron size range) fibers. These fibers possess a high surface‐to‐volume ratio, high aspect ratio, controlled pore size, and superior mechanical properties than conventional fibers. Electrospinning can be used to produce novel fibers with diameters between 100 nm and 10 µm. Electro‐spun fibers have been investigated as sensor, LEDs, rechargeable batteries, electroactive actuators, nano‐electronic devices, and electromagnetic shielding for wearable electronics applications.^[^ ^269^, ^270^ ^]^ Wei et al.,^[^ ^271^ ^]^ prepared core‐sheath nanofibers with conductive polyaniline as the core and an insulating polymer as the sheath by electrospinning of blends of polyaniline with polystyrene and polycarbonate. These unique core‐sheath structures offer potential in a number of applications including nanoelectronics. Huang et al., reported the development of high‐performance wearable SC fabrics based on flexible metallic fabrics (Ni–cotton), in which multi‐walled CNTs (MWCNTs)‐based nanofiber webs were directly electro‐spun, showing a high areal capacitance of 973.5 mF cm^−2^ (2.5 mA cm^−2^), Figure 13(e–i). The SC fabrics were also integrated into commercial textiles with desirable forms, demonstrating its potential for wearable electronics applications.^[^ ^267^ ^]^ **Table** **7** provides an overview of chronological research of several spinning techniques to produce conductive textiles.

**Table 7:** Comparison of several spinning techniques

Fiber /yarn	Spinning method	Polymer/doped with	Conductivity (*σ* in S cm^−1^)	Refs.
Polyaniline (PANI) (1999)	Air‐gap/ dry‐jet spinning	PANI/poly(p‐phenylene terephthalamide), PPD‐T, sulfuric acid solutions.	0.1	[272]
PANI/poly‐*ω*‐aminoundecanoyle (PA11) (2002)	Wet spinning	concentrated sulfuric acid (c‐H_2_SO_4_)	10^−5^ (5wt% PANI) to 10^−1^ (20% PANI)	[263]
PANI/polyamide‐11 (2001)	Wet Spinning	concentrated sulfuric acid (c‐H_2_SO_4_)	10^−6^ to 10^−1^ (3–16 wt.% PANI)	[273]
Microfibers of poly(3,4‐ethy‐lenedioxythiophene) (PEDOT) (2003)	Wet spinning	Poly (4‐styrene sulfonate) PSS	10^−1^	[274]
PANI/PP (2004)	Melt spinning	PANI/PP/DBSA	10^−9^	[266]
Core‐sheath nanofibers with a conductive polymer, PANI with PSS/PMMA/PC/PEO (2005)	Electrospinning	PANI/PS PANI/PMMA PANI/PC PANI/PEO	4.1 × 10^−14^ 4.3 × 10^−14^ 5.5 × 10^−14^ 2.4 × 10^−13^	[271]
PANI (2005)	Wet Spinning	dodecylbenzene sulfonic acid (PANI‐DBSA) and polyacrylonitrile containing methylacrylate (Co‐PAN)	10^−3^	[275]
PANI (2008)	Electrospinning	H_2_SO_4_ or HCl	52.9	[276]
PPy (2009)	Wet Spinning	di(2‐ethylhexyl) sulfosuccinate (DEHS)	30	[277]
PEDOT/PSS (2009)	Wet Spinning	dip‐treatment for 3 min EG (P grade) EG (PH grade)	195 467	[278]
PPy (2010)	Electrospinning	PPy‐MWCNTs composite	10^−1^	[279]
Poly(3,4‐ethylenedioxythiophene) poly(styrenesulfonate)–Polyvinyl Alcohol (PEDOT:PSS–PVA) nanofibers (2011)	Electrospinning	(0%, 3%, 5%, and 8%) of DMSO	4.8 × 10^−8^ to 1.7 × 10^−5^	[280]
PANI/PP (2011)	Melt Spinning	Polyaniline/PP/dodecylbenzene sulfonic acid (DBSA)	10^−9^	[281]
poly(3‐hexylthiophene) (P3HT) (2012)	Wet Spinning	FeCl_3_	160	[282]
poly(3‐hexylthiophene) (P3HT) (2012)	Melt Spinning	FeCl_3_ in nitromethane	350	[282]
PANI (2012)	Electrospinning	(+)‐camphor‐10‐sulfonic acid (HCSA) and blended with poly(methyl methacrylate) (PMMA) or poly‐(ethylene oxide)	50 ± 30 (100% doped PANI)	[283]
Ternary blend of PP/PA6/PANI (20% wt.)‐complex polypropylene / polyamide‐6 / polyaniline‐complex) (2012)	Melt Spinning		4 × 10^−3^	[284]
Binary blend fibers of PP / PANI (20% wt.)‐complex (2012)	Melt Spinning		5 × 10^−3^	[284]
poly(3‐hexylthiophene) (P3HT) nanofiber (2012)	Electrospinning	Iodine	122 ± 9	[285]
PEDOT‐PSS/PAN (2014)	Wet spinning	PAN	5	[286]
PEDOT:PSS/PEO fiber mats (2014)	Electrospinning	PEDOT:PSS	35.5	[287]
Graphene (1% wt.) / polyamide‐6 yarn (2017)	Melt Spinning		14 ± 2 × 10^−4^ (fiber draw ration 2.0)	[288]
Conductive filaments made of silk fibroin (SF) and PANI (2018)	Wet Spinning	SF–FA–shell solution	1.42 × 10^−4^ (0.28 wt. % PANI)	[289]
PANI/PEO side‐by‐side bi‐component fiber (2019)	Electrospinning	PAni doped with Camphoric acid CSA. Insulating PEO (3/4/5 w/v% to solvent) & conductive PANI‐CSA (1.5/2.5/3.5 w/v% to solvent)	10^−6^ to 10^−4^	[290]
PANI/PVP (polyvinylpyrrolidone) (2020)	Electrospinning	N,N‐dimethylformamide (DMF)	Membranes 1.7 × 10^−2^ and yarns 4.1 × 10^−4^	[291]
PANI−poly(ethylene oxide) (PEO) fibers (2020)	Electrospinning	(+)‐camphor‐10‐sulfonic acid (CSA), m‐cresol, and chloroform	1.73	[292]

### 4.4 In Situ Growth of Active Material on Substrate

Electrodeposition is a well‐known method of producing in situ metallic coatings or thin films of oxides and/or hydroxides by applying an electric current to a conductive material immersed in a solution containing a metal salt. By controlling several experimental parameters including potential, current density, deposition time, and plating solution composition, the morphology and texture of the film can be modified.^[^ ^293^ ^]^ The process may either be anodic or cathodic. In anodic process, a metal anode is electrochemically oxidized in the solution, and then deposited on anode. In a cathodic process, components (ions, clusters, or NPs) are deposited onto cathode from solution precursors. The increase in the reaction time causes more source materials deposition resulting in larger film thickness. The deposition rate can be maintained by the variation of current with time.^[^ ^294^ ^]^ E. Gasana et al.^[^ ^295^ ^]^ reported an electroconductive polyaramide woven textile structure produced via electroless deposition of PPy and copper at a deposition time of 240 s, providing a resistance of 5±1 Ω with a surface coverage of 100±1%. Zhao et al.,^[^ ^296^ ^]^ deposited copper galvanostatically in the copper citrate complex anions on poly (ethylene terephthalate) (PET) fabric treated with polyaniline (PANI) to produce flexible Cu–PANI/PET conducting textiles.

**Figure 14:**
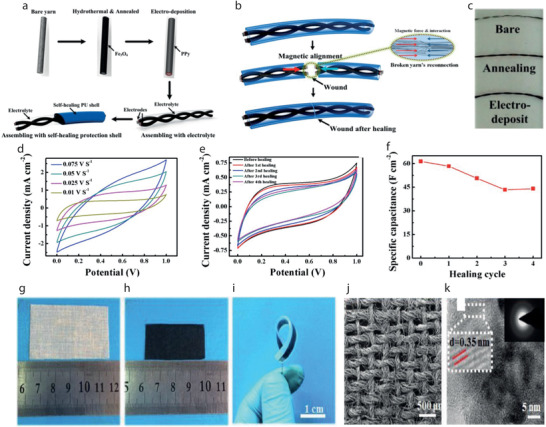
a) Design and manufacturing process flow of magnetic‐assisted self‐healable supercapacitor. Fe_3_O_4_ nanoparticles anchor on the surface of yarn by a microwave‐assisted hydrothermal method. The processed yarn is annealed to ensure the magnetic particles anchor on the yarn tightly. To achieve a better electrochemical performance, a layer of PPy is electrodeposited on the annealed yarn. Finally, two yarns as a set of electrodes are assembled with a solid electrolyte and a self‐healing shell to form a self‐healing supercapacitor. b) Schematic illustration of supercapacitor's self‐healing process. The magnetic alignment could assist the reconnection of fibers in broken yarn electrodes when they are brought together, as shown in inset image. c) From top to bottom, pristine yarn, hydrothermal and annealing‐treated yarn, and PPy‐electrodeposited yarn. Electrochemical measurements for as‐prepared capacitor. d) CVs obtained at various scan rates. e) CVs after healing for different cycles. f) Specific capacitance of the original device and after healing for different cycles. Reproduced with permission.^[^ ^299^ ^]^ Copyright 2015, American Chemical Society. g) Fabrication process and characterization of carbonized plain weave cotton fabric (CPCF)‐ Photograph of a pristine cotton fabric h) Photograph of the CPCF made from (g). i) A flexible CPCF‐based strain sensor. j,k) SEM image and TEM image of the CPCF. Reproduced with permission.^[^ ^306^ ^]^ Copyright 2016, Wiley‐VCH.

Hydrothermal reaction is a synthesis mechanism, which involves chemical reactions of substances in a sealed and heated aqueous solution or organic solvent at an appropriate temperature (100–1000 °C) and pressure (1–100 MPa). Many compounds or materials with special structures and properties, which cannot be prepared from solid‐state synthesis, can be obtained via hydrothermal reactions. In some cases, it offers an alternative and mild synthetic method for solid‐state reactions by lowering the reaction temperature. Hydrothermal synthesis has been successful in the preparation of important solids such as zeolites, open‐framework compounds, organic‐inorganic hybrid materials, MOF materials, superionic conductors, chemical sensors, electronically conducting solids, complex oxide ceramics and fluorides, magnetic materials, and luminescence phosphors. It is also a route to unique condensed materials including nanometer particles, gels, thin films, equilibrium defect solids, distinguished helical and chiral structures, and particularly stacking‐sequence materials.^[^ ^297^ ^]^ Hydrothermal synthesis relies on the forced hydrolysis of the reactants in order to produce the oxide ceramics. This is achieved at moderate temperatures (<200 °C) and high pressures by placing the reagents in a sealed container and heating the system to the reaction temperatures. The solvent is usually water; a metal hydroxide (e.g., NaOH) is added as a mineralizer while metal alkoxides or metal salts serve as the source of metal ions. As in precipitation systems, the nucleation is followed by particle growth to yield a powder with a certain particle‐size distribution.^[^ ^298^ ^]^ Huang et al.^[^ ^299^ ^]^ demonstrated the synthesis of Fe_3_O_4_ through hydrothermal reaction, wrapped on stainless steel fiber (SSF) which assisted the self‐healing of a yarn‐based SC to enhance the reliability and lifetime of a SC in practical usage, **Figure** **14**(a–f). The specific capacitance was restored up to 71.8% even after four breaking/healing cycles with great maintenance of the whole device's mechanical properties. Li et al.^[^ ^300^ ^]^ reported the fabrication of hierarchical graphene fiber fabrics (GFFs) with significantly enlarged specific surface area using a hydrothermal activation strategy. The achieved areal capacitance was 1060 mF cm^−2^ with a very thin thickness (150 µm) and further magnified up to 7398 mF cm^−2^ by overlaying several layers of HAGFFs.

In situ polymerization is another fabrication technique for conductive electrodes. This is typically a chemical encapsulation process similar to interfacial polymerization except there are no reactive monomers in the organic phase. All polymerization occurs in the continuous phase rather than in the interface as in interfacial polymerization. The most common example of this method is the condensation polymerization of urea or melamine with formaldehyde to form cross‐linked urea‐formaldehyde or melamine‐formaldehyde capsule shells. In this method, an oil‐phase is emulsified in water using water‐soluble polymers and high‐shear mixers, yielding a stable emulsion at the required droplet size. A water‐soluble melamine resin is added and dispersed. The pH of the system is then lowered, initiating the polycondensation which yields crosslinked resins that deposit at the interface between the oil droplets and the water phase. During the hardening of the wall material, the microcapsules are formed and the aqueous dispersion of polymer‐encapsulated oil droplets is produced.^[^ ^301^, ^302^ ^]^ Lee et al.^[^ ^303^ ^]^ reported 1D metal oxide nanostructure‐based SC of multiscale architecture. In their work, MnO_2_‐micronodules were deposited on carbon cloth, followed by coating with partially carbonized polypyrrole (CPPy) through vapor deposition polymerization (VDP). Then, the PPy‐coated MnO_2_‐based multiscale micronodules were assembled within a PVA–KOH polymer electrolyte as the positive‐electrodes of solid‐state asymmetric SCs (ASCs) which demonstrated ultrahigh performance (59.5 F cm^−3^ of capacitance, 27.0 mWhcm^−3^ of energy and 1.31 Wcm^−3^ power density).

Carbon fiber, though exhibit high conductivity, suffer from agglomeration problem which creates obstacle during application in several fields. Additionally, the small surface area and low specific capacitance limit the wide‐scale use as conductive material, therefore surface functionalization offers scopes to overcome the limitations and exploit the fiber properties fully. Oxidation (wet and dry), amidation, silanization, silylation, polymer grafting, polymer wrapping, surfactant adsorption, thermal annealing, and encapsulation are some of the functionalization techniques.^[^ ^304^ ^]^ Cotton, the most popular fiber ever, can be considered as an innovative platform for wearable, smart and interactive electronic devices, like batteries, SCs, and various sensors if their electrochemical performances are ensured. It is a natural polymer of cellulose, burns readily, but in low oxygen concentration, it chars leaving a carbon skeleton, which improves the conductivity.^[^ ^305^ ^]^ Carbonization/Pyrolysis of cotton fibers can be an alternative option to produce conductive electrodes. Zhang et al.,^[^ ^306^ ^]^ simply annealed pristine woven cotton fabric in an inert atmosphere, producing flexible and highly conductive fabric, which was used as a strain sensor to demonstrate its superior performance in the detection of both large and subtle human body motions, Figure 14(g–k). However, the release of toxic substances during carbonization process limits the viability of the process.

## 5 Fabrication of Textiles Supercapacitors

### 5.1 Device Configurations

**Figure 15:**
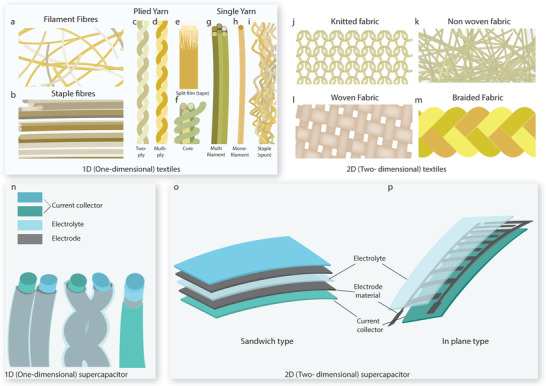
Textile‐based energy storage device configuration. 1D substrate forms a) filament fibers, b) staple fibers, c) two plied, d) multiplied, e) split film, and f) core‐sheath yarn, g) multifilament, h) monofilament, and i) staple yarn. 2D substrate forms j) knitted, k) non‐woven, l) woven, and m) braided fabric. Textile supercapacitor forms n) 1D fiber or yarn shaped o) Sandwich type and p) in plane type 2D supercapacitor.

Textile‐based flexible energy storage devices that are used for wearable applications can be categorized into: 1D fiber/yarn shaped device and 2D fabric shaped device. Additionally, there are very few 3D‐shaped energy storage devices that have been reported in the literatures.^[^ ^307^ ^]^ **Figure** **15** represents the schematic diagram of textile substrates that have been exploited as 1D and 2D‐shaped storage devices.

#### 5.1.1 1D Fiber/Yarn Shaped Supercapacitors

In this type, energy storage device or SC components (i.e., the current collector, electrode, separator, and electrolyte) are all integrated into 1D system, possibly in fiber or wire or cable‐shaped SCs. Several forms of 1D‐shaped textiles are: filament or staple fibers converted into two ‐or multi‐plied yarn, split film, core‐sheath yarn, multi‐ or mono‐ filament, and staple yarn, Figure 15(a–i). They are generally small in size, light in weight, and possess dimensions typically ranging from tens to hundreds of micrometers in diameter, and several millimeters to meters in length.^[^ ^64^, ^308^ ^]^ For instance, the electrode materials with separator are twisted to form ply yarn or all the electrodes and electrolyte are wrapped together to form a single yarn of core‐sheath configuration, Figure 15n. 1D electrochemical energy storage devices offer several potential advantages over other conventional SCs including mechanical flexibility and deformability under various bending and twisting conditions, enabling better wearability and integrability into flexible textiles. The ease of integration via weaving, knitting, stitching, or embroidery provides scopes to get assembled into various shapes at different desirable locations in several wearable devices. It enables a greater design versatility and scope of integration with 1D energy harvesting (EH) or other devices such as displays and sensors, to create multifunctional wearable systems.^[^ ^309^ ^]^ However, the increased electrical resistance along the device length is a major challenge that affects the electrochemical performance of such devices.^[^ ^210^ ^]^

#### 5.1.2 2D Fabric Shaped Supercapacitors

2D planar SC devices are particularly suitable for use in thin or layered products, such as smart cards, packaging and labels, magazines, books, skin patches and healthcare devices, jewelries, and a broad range of other products comprising flexible electronic components.^[^ ^210^ ^]^ Different substrates are usually used to fabricate 2D SCs including plastic films, sponge, metal sheets, papers, and textiles.^[^ ^310^ ^]^ Among them, textile fabrics offer excellent flexibility due to their intrinsic mechanical properties and ability to be integrated directly with any other textile fabrics or garments for wearable applications via any simple methods of joining (e.g., sewing technology).^[^ ^1^ ^]^ Any form of fabric including woven, knit, non‐woven, or braided can be used for 2D SCs, Figure 15(j–m). In such a 2D configuration, a pair of fabric electrodes are usually separated by an electrolyte and a separator in a sandwich or planar structure, Figure 15(o,p). However, it is challenging to maintain the appropriate dimension/thickness of such configurations in order to achieve high areal device performance, flexibility, and comfort.^[^ ^33^ ^]^

#### 5.1.3 3D Energy Storage Device

The existing fabrication techniques of electrochemical energy storage devices have limitations in controlling the geometry and architecture of electrodes and solid‐state electrolytes, which limits the charge storage performance for most electrodes.^[^ ^311^ ^]^ The potential solution to improve the areal capacitance and energy density of EES devices is to build thicker electrodes, ensuring the increase of active materials loading without sacrificing the fast ion diffusion. In the case of 2D structures, the ion transport distance and overall electrical resistance of the thicker electrodes increase inevitably, causing a decrease in rate capability and power density.^[^ ^312^ ^]^ In contrast, a 3D structure provides shorter diffusion pathways and smaller resistance during the ion transport process.^[^ ^313^ ^]^ It also effectively enhances the energy density by creating porous structure and efficiently utilizing the available limited space.^[^ ^314^ ^]^ 3D structured devices with high energy and power density, lightweight, and well‐controlled geometry within an architecture (all in a miniaturized package) are being experimented nowadays, to enhance electrochemical performance and safety.^[^ ^312^ ^]^ Additionally, 3D flexible conductors are mechanically durable and more promising in comparison to their 2D counterparts in maintaining their functionalities when subject to various mechanical deformation, such as bending, stretching, shearing, compressing, and twisting.^[^ ^315^ ^]^ 3D printing, also known as “Additive Manufacturing” (AM), has gained much attention as a powerful manufacturing technique for the fabrication of 3D‐structured EES devices. Such technique provides freedom for designing complex 3D prototypes and devices from the macroscale to nanoscale range in a programmable, facile, rapid, cheap, and flexible manner.^[^ ^316^ ^]^ Successive layers of selected materials are deposited together by following a digital 3D model guidance using computer‐aided design (CAD) and computer‐aided manufacturing (CAM). 3D printed electrochemical devices for energy storage, conversion, and/or sensor have the potential to be used in various sectors including healthcare, biomedicine, pharmaceutical, engineering, etc.^[^ ^317^, ^318^, ^319^ ^]^ It also provides great opportunities to accurately control device spatial geometries and architectures, offers greater control over electrode thickness with simplified and low‐cost process enhancing both the energy density and power densities.^[^ ^312^, ^320^ ^]^ Additionally, it allows the manufacturing of complex‐shaped SCs, as well as offers flexibility in packaging due to a wide range of 3D shapes.^[^ ^321^ ^]^

### 5.2 Integration of the EES Device

**Figure 16:**
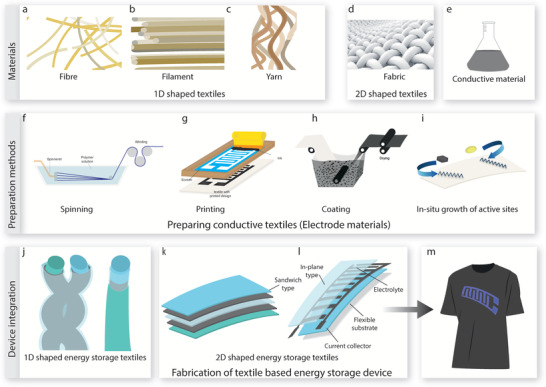
Fabrication of textile‐based supercapacitor devices. 1D shaped textiles, a) fiber, b) filament, c) yarn, and 2D shaped fabric and c) conductive materials. Preparation of conductive textiles by f) spinning, g) printing, h) coating, and i) in situ growth of active sites on textiles to produce. j) 1D shaped energy storage textiles, 2D shaped k) sandwich type and l) in‐plane type supercapacitor, and m) the final e‐textiles.

After the formation of the electrode materials, there comes the final but critical stage of the integration of the components to complete the full SC. LIB cells are typically parallel assembled in modules (with internal electrical circuits), which are then integrated within a battery management system. Cable‐shaped 1D devices (e.g., for smart textiles) can be embedded into textiles by weaving, knitting, or embroidery.^[^ ^210^ ^]^ Conductive electrodes and separators along with electrolytes are all integrated on a simple wire/cable shape, **Figure** **16**j. 2D planar or thicker 3D devices may be integrated on a chip or a soft substrate into planar flexible objects. Electrodes with separators and/or electrolytes can be sandwiched by compression and/or encapsulation to form the integrated device, Figure 16(k,l). However, there exist requirements for simpler and low‐cost assemblies for flexible and wearable device architectures. Additionally, the encapsulation of the integrated device is crucial to improve the washability and durability of wearable e‐textiles devices. Several methods have been used, either as surface pre‐treatments (e.g., Bovine Serum Albumin (BSA) treatment) or post‐treatments (e.g., PDMS, PU coatings), to seal conductive track and encapsulate integrated devices, protecting them from the exposure to harsh treatments during daily usages.^[^ ^225^ ^]^

### 5.3 Combined Energy Harvesting and Storage

**Figure 17:**
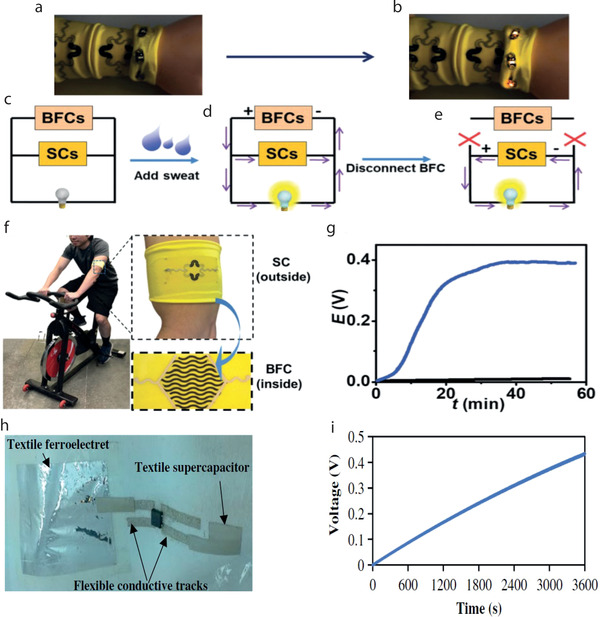
a,b) Demonstration of the hybrid SC–BFC device, Photographs showing the application of three SCs charged by five BFCs to light LEDs using the following procedure: c) without lactate, no power; d) with lactate, LEDs were turned on; e) upon disconnecting BFCs and SCs, LEDs could still be turned on. f) The integrated chemical self‐powered system on one piece of textile was applied to the arm of a volunteer. The SC and BFC were printed outside and inside the textile band, respectively. g) The real‐time voltage of the printed SC charged from the on‐body BFC during a constant cycling exercise for 56 min. The SC charged by lactate BFC immobilized with LOx (blue plot) and without LOx as a control (black plot). Reproduced with permission.^[^ ^331^ ^]^ Copyright 2018, The Royal Society of Chemistry. h) Assembled textile power module and i) FEP‐textile ferroelectret charging the 2 mF textile capacitor. Reproduced with permission.^[^ ^332^ ^]^ Copyright 2019, Wiley‐VCH.

The EH devices harvest energy that dissipates around us, in the form of electromagnetic waves, heat, and vibration, and then convert them into easy‐to‐use electric energy with relatively small levels of power in nW‐mW range.^[^ ^322^, ^323^ ^]^ The principle is similar to large‐scale renewable energy generations such as wind turbines, but with a smaller amount of energy produced from such devices. EH is a promising technique for solving the global energy challenge without depleting natural resources and as an everlasting source of power supply.^[^ ^324^ ^]^ It can reduce greenhouse gas emissions generated with traditional energy sources.^[^ ^325^ ^]^ Though cheap, conventional batteries limit the amount of energy, and therefore require periodic replacement or recharging. In addition to that, rigid bulky structure limits their usage in smart fabrics applications.^[^ ^326^ ^]^ Flexible EH devices thus have the potential to replace conventional power sources for wearable electronics. EH sources are classified into two groups according to the characteristics of their source, i) Natural sources are those available readily from the environment such as sunlight, wind, and geothermal heat and ii) artificial sources are those generated from human or system activities including human motion, pressure on floors/shoe inserts when walking or running, and system vibration when operating.^[^ ^327^ ^]^ Biomechanical EH from human motion has attracted attention in the past decade for potential applications in charging portable electronic device, batteries, and self‐powered sensor systems.^[^ ^328^, ^329^ ^]^ Satharasinghe et al.^[^ ^330^ ^]^ presented an innovative solar EH fabric and demonstrated its suitability for powering wearable and mobile devices. A large solar EH fabric containing 200 miniature solar cells was demonstrated which can charge a 110 mF textile SC bank within 37s. Lv et al.^[^ ^331^ ^]^ demonstrated the first example of a stretchable and wearable textile‐based hybrid supercapacitor–biofuel cell (SC–BFC) system, screen‐printed on both sides of the fabric, designed to scavenge biochemical energy from the wearer's sweat and store it in the SC module for subsequent uses, **Figure** **17**(a–g). Yong et al.^[^ ^332^ ^]^ presented a textile‐based power module for the first time that combines a ferroelectric biomechanical energy harvester and solid‐state SC‐based energy storage device, fabricated in a single woven cotton textile layer, Figure 17(h,i). The textile power module was highly flexible, and the fluorinated ethylene propylene (FEP) based ferroelectret was able to generate electric energy with an instantaneous output voltage of ≈10 V and power density of ≈2.5 µW cm^−2^, with a solid‐state SC having a capacitance of 5.55 mF cm^‐2^.

## 6 Electrochemical Performance of Textile‐Based Supercapacitor Devices

Capacitance/capacity per unit length (F cm^−1^), area (F cm^−2^), or volume (F cm^−3^) are usually reported to evaluate the performance of a textile‐based SC. However, the gravimetric capacitance of electrode materials does not necessarily represent the full device capacitive performance. In addition to capacitance, the other two key parameters for evaluating SC performance are energy density and power density. Maintaining higher energy and power density is still a major challenge for such energy storage devices. SCs, in comparison with lithium‐ion batteries, usually exhibit relatively lower energy density but higher power density. Whereas for LIBs, it is desirable to improve the power density while keeping the high energy density. The major challenge in achieving high‐performance SC textiles is to enhance the energy density while maintaining the high‐power density.^[^ ^33^ ^]^

The capacitance retention of textile‐based energy storage devices is another important property for wearable applications, since the replacement of such devices would be difficult during the product lifetime. In most cases, 10 000 cycles of charge‐discharge are employed for the capacitance retention assessment. For 1D‐shaped energy storage devices, several configurations were reported with full capacitance retention up to 10 000 cycles.^[^ ^333^, ^334^ ^]^ Additionally, Fu et al.^[^ ^335^ ^]^ reported a fiber‐shaped SC, developed by pen ink, which retains full capacitance even after 15 000 of charge‐discharge cycles. For 2D‐shaped energy storage devices, full capacitance retentions were reported even after longer (20 000–25 000) charge‐discharge cycles.^[^ ^336^, ^337^ ^]^ Wang et al.^[^ ^338^ ^]^ developed a fabric type asymmetric SC using electrochemically activated carbon cloth as anode, TiN@MnO_2_ on carbon cloth as cathode, and LiCl solution as electrolyte, which significantly boosted the energy storage capability. The device showed no capacitive decay even at 70 000 cycles of charge‐discharge. In another study, Hu et al.^[^ ^223^ ^]^ reported a fabric‐based SC device made of Cotton/SWNTs, exhibiting extremely good cycling stability of 98% capacitance retention over a remarkably large cycle number of 130 000 charge‐discharge cycles. As we previously classified the active materials into four categories, in this section we will discuss active materials of textile‐based SCs. Each subsection focuses on both 1D and 2D shaped textile SCs. Combining any of the two or more types are also summarized under hybrid materials section. In addition, we throw light on SCs based on fiber types, particularly focusing on most widely used natural cotton, manmade polyester and carbon fibers.

### 6.1 Carbon‐Based Textiles Supercapacitors

Among all the active materials, carbonaceous compounds, that is, CNTs, graphene and its derivatives have mostly been studied for the fabrication of textile‐based SC devices. **Table** **8** summarizes the electrochemical performances of carbon‐based 1D fiber/yarn‐shaped and 2D fabric‐shaped SCs. Several research groups reported graphene fiber,^[^ ^339^ ^]^ GO fiber,^[^ ^340^ ^]^ rGO fiber,^[^ ^341^ ^]^ and modified rGO^[^ ^342^ ^]^ fiber as SC electrodes. Kou et al.^[^ ^264^ ^]^ twisted two coaxial fibers composed of polyelectrolyte‐wrapped carbon nanomaterial core‐sheath fiber, rGO@CMC, CNT@CMC, rGO+CNT@CMC, together to form a two‐ply SC electrode, and then coated with PVA electrolyte. In presence of 1 m H_2_SO_4_ liquid electrolyte, the device exhibited length, areal, and volumetric capacitance of 8.0 mF cm^−1^, 269 mF cm^−2^, 239 F cm^−3^, respectively. Zhai et al.^[^ ^343^ ^]^ drop‐casted activated carbon on carbon fiber yarn to prepare SC electrode. For the fabrication of yarn‐shaped SC, two coated strands were twisted together and finally dipped in PVA‐H_3_PO_4_ electrolyte. The resultant device exhibited a specific length capacitance of 45.2 mF cm^−1^ at a scan rate of 2 mV s^−1^.

**Table 8:** Summary of Carbon‐based supercapacitors or supercapacitor electrodes

1D shaped							
Substrate (Reporting year)	Device Configuration	Device capacitance	Energy density	Power density	Capacitance retention	Flexibility	Refs.
Carbon fiber and Gold coated plastic fiber (2012)	Commercial graphite pen ink used as active material and deposited on fiber surface. Spacer wire evenly twisted onto the surface of one fiber electrode, Two fiber electrodes placed parallelly in a flexible plastic tube filled with PVA‐H_2_SO_4_ electrolyte	11.9–19.5 mF cm^−2^	1.76×10^−6^ –2.70×10^−6^ Wh cm^−2^	Up to 9.07 mW cm^−2^	Remains similar after 15 000 cycles	Only slight drop at 180°, 360° bending	[335]
Carbon microfiber bundle (2013)	A carbon microfiber bundle coated with MWCNTs as a core electrode in the center of the coaxial SC and a carbon nanofiber (CNF) film prepared by electrospinning as an outer electrode	6.3 mF cm^−1^ (86.8 mF cm^−2^)	0.7 µWh cm^−1^ (9.8 µWh cm^−2^)	583 µW cm^−1^	94% after 1000 cycles	Negligible change at 180° bending	[349]
Carbon fiber (2013)	Electrochemically reduced GO(ERGO) coated carbon fiber followed by acid treatment (ERGO@CF–H) with PVA–H_3_PO_4_ gel electrolyte	13.5 mF cm^−1^ (307 mF cm^−2^) at 0.05 mA cm^−1^	1.9 mW h cm^−1^ (21.4 mW h cm^−2^)	0.74 mW cm^−1^ (8.5 mW cm^−2^)	85% after 5000 cycles	No decay at bending 0°–180°	[350]
rGO fiber yarns (2014)	rGO fiber yarns deposited on a titanium current collector and separated by a PVDF membrane	409 Fg^−1^	14 Wh kg^−1^	25 kW g^−1^	No decay after 5000 cycles		[341]
Carbon nanotube and rGO composite yarn (2014)	Two SWNTs/rGO electrode mounted on a polyester (PET) substrate using PVA–H_3_PO_4_ electrolyte	116.3 mFcm^−2^, 45 Fcm^−3^ at 26.7 mA cm^−3^	6.3 mWh cm^−3^	1085 mW cm^−3^	93% after 10 000 cycles	>97% after 1000 bending cycles at 90°	[351]
GO, carbon nanotube (CNT) and their mixture wet spun filament (2014)	Polyelectrolyte‐wrapped graphene/carbon nanotube core‐sheath fibers, Polyelectrolyte‐wrapped carbon nanomaterial (graphene, CNTs, and their mixture) core‐sheath fiber, RGO@CMC, CNT@CMC, RGO+CNT@CMC, Two coaxial fibers twisted together to form two‐ply SC electrodes, then coated with PVA electrolyte	269 mF cm ^−2^, 239 F cm^−3^, 8.0 mF cm^−1^ (liquid electrolyte 1 m H_2_SO_4_), 177 mF cm ^−2^, 158 F cm^−3^, 5.3 mF cm^−1^ @ current density of 0.1 mA cm^−2^ (solid electrolyte H_3_PO_4_/PVA)	5.91 mWh cm ^−2^ (liquid) 3.84 mWh cm ^−2,^ 3.5 mWh cm^−3^ (solid)		No decay within 2000 times	Dropped 2% at 200 times of bending and rose persistently up to 111% at 1000 times of bending	[264]
GO fiber (2014)	Region‐specific reduction of GO fiber by laser irradiation, to prepare rGO/GO/rGO single fiber SC with ionic liquid electrolyte of 1‐butyl‐3‐mthylimidazolium tetrafluoroborate.	1.2mF cm^−2^ at 80 µA cm^−2^, 0.45 mF cm^−2^ at 200 µA cm^−2^	2–5.4×10^−4^ Wh cm^−2^	3.6–9×10^−2^ W cm^−2^	No obvious degradation after 1000 cycles	No decrease after 160 bending cycle	[340]
Carbon fiber yarn (2015)	Two hybrid activated carbon drop casted carbon fiber yarn electrodes, twisted together, dipped in PVA/H_3_PO_4_ gel electrolyte, dried	Specific length capacitance 45.2 mF cm^−1^ at 2 mV s^−1^	Specific length energy 6.5 µWh cm^−1^	27 µWcm^−1^	86.6% after 10 000 cycle	98% after 1000 bending cycles	[343]
CNT fiber (2015)	GO sheets coated on CNT fiber, reduced to RGO, forming a core–sheath‐structured CNT/RGO composite fiber. Two composite fibers drawn into a gel electrolyte consisting of poly(vinyl alcohol) (PVA), phosphoric acid, and water, followed by twisting into fiber‐shaped SC	68.4 F cm^−3^ (126.7 F g^−1^) at 31 mA cm^−3^	2.4 mW h cm^−3^, 3.8 µW h cm^−2^	0.016 W cm^−3^, 0.025 mW cm^−2^	No decay after 10 000	No decrease at bending 180°	[334]
Graphene fiber (2015)	Spun‐rGO//rGO Coaxial all graphene fiber SC with a continuous liquid crystal wet‐spun core fiber electrode followed by reduction, PVA gel coating as separator, a dip‐coated cylinder sheath fiber as the other electrode followed by reduction, coating of H_2_SO_4_/PVA gel electrolyte.	205 mF cm^−2^ (182 F g^−1^)	17.5 µWh cm^−2^ (15.5 Wh kg^−1^), Increased to 104 µWh cm^−2^ with organic ionic electrolyte		No decay at 10 000th cycle	92% after 100 times of bending	[339]
Graphene fiber (2015)	Two MWCNTs‐rGO fiber electrodes with PVA‐H_3_PO_4_ electrolyte	0.35 mF cm^−1^, 38.8 F cm^−3^ at 50 mA cm^−3^	3.4 mWh cm^−3^	700 mW cm^−3^	93% after 10 000 cycle	No decrease after knotting	[352]
Porous graphene ribbon (PGRs) (2015)	The PGRs freeze‐dried for 24 h to obtain the dried porous graphene ribbons (DGRs) followed by immersion in H_3_PO_4_/PVA electrolyte solution. Two pairs of electrodes pressed together under slight pressure to fabricate the PGR and DGR SC.	208.7 F g^−1^ (78.3 mF cm^−2^ or 3.12 mF cm^−1^)			99% after 5000 cycles	No decrease at bending 45°, 90°, 135°, 180°, 95% after 100 cycle bending when woven to glove	[353]
GO fiber (2015)	Region‐specific reduction of wet spun GO fiber by laser irradiation to prepare alternate rGO‐GO electrolyte‐free fiber SCs	14.3 mF cm^−2^ at 50 mA cm^−3^			93% after 1000 cycles	No decrease after 1000 cycles	[354]
PVA/RGO hybrid fibers (2016)	Incorporating hydrophilic PVA into a non‐liquid‐crystalline GO dispersion before wet spinning and chemical reduction, two bundles of PVA/RGO fibers with PVA/ H_2_SO_4_/ H_2_O gel electrolyte	Fiber 241 F cm^−3^	5.97 mW h cm^−3^ (5.32 mW h g ^−1^)	At 26.9 mW cm^−3^ (23.9 mW g^−1^)	85% after 1000 cycles	97% retention after cyclic bending between 0 and 180 for 1000 times	[342]
Graphene and few‐walled carbon nanotubes composite yarn (2018)	Hybrid fiber based on graphene and few‐walled carbon nanotubes (G_10_ /CNTs) electrode with 6 M KOH aqueous electrolyte	312.6 F g^−1^ at 200 mA g^−1^	Varied from 23.46 to 9.66 Whkg^−1^ at 84.68 to 1134.56 Wkg^−1^		89.6% after 10 000 cycles		[355]

2D shaped							
Substrate (Reporting year)	Device Configuration	Device capacitance	Energy density	Power density	Capacitance retention	Flexibility	Refs.
Cotton fabric (2010)	Dipping and drying of cotton with single‐walled carbon nanotube (SWNT) ink electrodes with LiPF_6_ electrolyte to form fabric SC	140 Fg^−1^ at 20 µA cm^−2^, 0.48 F cm^−2^	20 Wh kg^−1^ at 10 kW kg^−1^		98% after 130 000 cycles	No decrease @100th 120% strain	[223]
Cotton Stretchable fabric (2010)	Dipping and drying of cotton with single‐walled carbon nanotube (SWNT) ink electrodes with LiPF_6_ electrolyte to form fabric SC	62 Fg^−1^ at 1 mA cm^−2^			<6% decrease after 8000 cycles	Almost similar after stretching 120% strain 100 times.	[223]
Cotton fabric (2010)	Conformal coating of single‐walled carbon nanotubes (SWNTs) ink with 2 molL^−1^ Li_2_SO_4_ electrolyte	70–80 Fg^−1^ at 0.1 mA cm^−2^			Coulombic efficiency >99% after 35 000 cycles		[356]
Cotton cloth (2012)	Brush‐coating of cotton cloth with GO suspension ink as electrode, nickel foam current collector, pure cotton cloth as separator, and 6 m KOH electrolyte	81.7 Fg^−1^	7.13 Wh kg^−1^	1.5 kW kg^−1^	93.8% after 1500 cycles		[357]
Cotton woven (2017)	Screen printing of GO, followed by electrochemical reduction to produce rGO‐cotton electrode, with PVA‐H_2_SO_4_ gel electrolyte	2.5 mF cm^−2^, 257 F g^−1^			97% after 10 000 cycles	95.6% after folding 180° for 2000 cycles	[239]
Cotton fabric (2019)	Dry‐coated and subsequently two‐step reduced GO coated cotton fabric as electrode, raw cotton fabric separator, H_3_PO_4_ /PVA gel electrolyte sandwich to form flexible SC	464 F g^−1^ at 0.25 A g^−1^	27.05 W h kg^−1^		91.6% after 1000 cycles	No apparent performance degradation after 0° and 180° bending	[344]
Cotton fabric (2022)	Graphene ink screen printed on cotton textiles with PVA/H2SO4 gel electrolyte	3.2 mF cm^−2^	0.28 mWh cm^−2^ at 3 mW cm^−2^		95% after 10 000 cycles		[358]
Polyester fabric (2014)	CNT dip‐coated onto PET fabrics electrode	1.4×10^−4^ F cm^−2^					[359]
Polyester fabric (2019)	By sandwiching 2 dip‐dried graphene nanoparticle/ polyester electrodes and 1 h‐boron nitride/polyester dielectric layer in between to form flexible textile‐based capacitor (FTC)	26 pF cm^−2^				Sustains 100 cycles of repeated bending	[360]
Poly‐cotton (65/35) 2/1 twill fabric (2020)	Graphene‐coated textile electrodes with PVA‐H_2_SO_4_ gel electrolyte	2.7 mF cm^−2^ (electrode)			≈98% after 15 000 cycles	98% after bending 150 cycles at 180°	[225]
Cotton and polyester fabric (2011)	Cotton and polyester screen printed with activated carbon as SC electrode, polyester separator, and Li_2_SO_4_ and Na_2_SO_4_ electrolyte	Electrode 0.43 F cm^−2^ at 5 mA cm^−2^ (Na_2_SO_4_), 85–95 Fg^−1^ at 1–10 mVs^−1^			92% after 10 000 cycles		[345]
Polypropylene fabric (2019)	Reactive inkjet printing of rGO layers on PP fabric as electrode with PVA/H_3_PO_4_ gel electrolyte to form flexible solid‐state SC	13.3 mF cm^−2^ (79.9 F g^−1^) at 0.1 mA cm^−2^	1.18 mWh cm^−2^	4.6 mW cm^−2^	Almost 100% after 5000 cycles		[361]
Carbon fabric (2012)	Entangled carbon nanofibers (CNFs) were synthesized on a flexible carbon fabric (CF) via water‐assisted chemical vapor deposition to form CNF/CF electrode with 0.5 m Na_2_SO_4_ aqueous neutral solution	140 F g^−1^ at 5 mV s^−1^			≈95% after 2000 cycle		[362]
Carbon fiber knit and woven fabric (2013)	Carbon fabric screen printed with activated carbon with solid polymer electrolyte	88 Fg^−1^, 0.51 F cm^−2^ (Knitted), 66 Fg^−1^, 0.19 F cm^−2^ (Woven)				80% after bending at 90°, 135°, and 180°	[346]
Carbon cloth (2014)	Electrochemically activated carbon cloth electrode with PVA‐H_2_SO_4_ electrolyte	Electrode 88 mF cm^−2^ (8.8 mFg^−1^) at 10 mVs^−1^, SC 15.3 mF cm^−2^ (0.765 mFg^−1^)			95% after 20 000 cycles		[336]
Activated Carbon fiber felt (2015)	Carbon nanotube (CNTs) and graphene (GN) modified composite ACFF textile electrodes, non‐woven fabric separator, with KOH aqueous electrolyte	3350 mF cm^−2^, device 2700 mFcm^−2^	112 µW h cm^−2^	490 µW cm^−2^	No decay at 1000 cycles		[347]
Stainless steel fabric (2016)	Two chemically converted graphene (CCG) on Stainless steel fabrics (SSF) electrode with 1 m H2SO4 to form flexible solid‐state symmetrical SC	730.8 mF cm^−2^ at 2 mA cm^−2^, 180.4 mF cm^−2^ at 1 mA cm^−2^	19.2 W h cm^−2^ at 386.2 W cm^−2^		96.8% after 7500 cycles	96.4% after 800 stretching‐bending cycles	[348]
Silver fiber fabric (2017)	Electrophoretic deposition of graphene on silver fiber fabric (rGO/SFF) electrode with KOH (3 m) as electrolyte	172 mF cm^−2^ at 4 mA cm^−2^			97% after 5000 cycles		[363]

For 2D fabric‐shaped SC conventional cotton, polyester, poly‐cotton, PP, and carbon fiber textiles (CFTs) were investigated. Several research groups^[^ ^223^, ^344^ ^]^ coated cotton fabric with single or MWCNT, however, better performances were obtained while using graphene or its derivatives. For example, Hu et al.^[^ ^223^ ^]^ coated cotton fabric with SWNT ink by simple dipping and drying method to obtain SC electrode. In the presence of LiPF_6_ electrolyte, the device exhibited specific capacitance of 140 Fg^−1^ at 20 µA cm^−2^ and an areal capacitance of 0.48 F cm^−2^. The energy density was as high as 20 Wh kg^−1^ at 10 kW kg^−1^ with an outstanding cyclic stability of 98% after 130 000 charge‐discharge cycles. In another study by Li et al.,^[^ ^344^ ^]^ the gravimetric capacitance was enhanced by coating cotton fabric with reduced GO and using coated fabrics as SC electrode. The sandwiched‐shaped SC device combined with raw cotton fabric separator and H_3_PO_4_/PVA gel electrolyte exhibited a gravimetric capacitance of 464 F g^−1^ at 0.25 A g^−1^ and a higher energy density of 27.05 W h kg^−1^.

The highest areal capacitance for conventional textiles SC prepared by carbonaceous compounds was reported by Jost et al.^[^ ^345^ ^]^ They screen printed activated carbon on both cotton and polyester fabric. The fabric SC achieved a high areal capacitance of 430 mFcm^−2^ at 5 mAcm^−2^ and a gravimetric capacitance of 85–95 Fg^−1^ at 10 mV s^−1^. The device also exhibited very good cyclic stability of 92% after 10 000 charge‐discharge cycles. Additionally, they screen printed activated carbon on carbon fiber fabric and achieved even higher areal capacitance.^[^ ^346^ ^]^ They achieved gravimetric capacitance of 88 Fg^−1^ and areal capacitance of 510 mF cm^−2^ for knitted carbon fiber fabric, and that of 66 Fg^−1^ and 190 mFcm^−2^ for the woven carbon fiber fabric. The highest areal capacitance for both electrode (3350 mFcm^−2^) and SC device (2700 mF cm^−2^) was obtained through composite electroactive materials, as reported by Dong et al.^[^ ^347^ ^]^ CNTs and graphene (GN) were coated on activated carbon fiber felt (ACFF) to prepare electrodes. The asymmetric SC was fabricated by as‐prepared CNT/ACFF and GN/ACFF composite textile electrodes with a non‐woven fabric separator, and KOH aqueous electrolyte. The energy and power densities were reported as 112 µW h cm^−2^ and 490 µW cm^−2^, respectively. Furthermore, several research groups have investigated stainless steel fabric (SSF) and silver fiber fabric for fabric‐based SCs. Yu et al.^[^ ^348^ ^]^ prepared two chemically converted graphene (CCG) on SSF electrode with 1 m H_2_SO_4_ to form flexible solid‐state symmetrical SC. The areal capacitance was reported as high as 730.8 mF cm^−2^ at 2 mA cm^−2^ and 180.4 mF cm^−2^ at 1 mA cm^−2^. The energy density was reported as 19.2 W h cm^−2^ at 386.2 W cm^−2^. The device was able to retain the capacitance up to 96.8% after 7500 charge‐discharge cycles and 96.4% after 800 stretching‐bending cycles.

### 6.2 Conductive Polymer‐Based Textiles Supercapacitors

CPs are pseudo‐capacitive materials, the bulk of the material undergoes a fast redox reaction to provide the capacitive response exhibiting superior specific energies to the carbon‐based double‐layer SCs.^[^ ^364^ ^]^ **Table** **9** summarizes the conductive polymer‐based 1D fiber or yarn‐shaped and 2D fabric‐shaped SCs. Only a few works have been reported for fiber or yarn‐shaped textile SCs. For example, Wei et al.^[^ ^365^ ^]^ directly coated cotton yarns by PPy nanotubes via in situ polymerization of pyrrole in presence of methyl orange. The electrode thus obtained was used to fabricate an all‐solid‐state yarn SC, which provided a high areal‐specific capacitance of 74.0 mF cm^−2^ and an energy density of 7.5 µWh cm^−2^.

**Table 9:** Summary of CP‐based supercapacitors or supercapacitor electrodes

1D shaped							
Substrate (Reporting year)	Device Configuration	Device capacitance	Energy density	Power density	Capacitance retention	Flexibility	Refs.
Cotton yarn (2017)	Cotton yarns coated with PPy nanotubes	74.0 mF cm^−2^	7.5 µWhcm^−2^			97% after 200 cycles	[365]
Carbon fiber thread (2015)	carbon fiber thread (CFT) @polyaniline (PANI) as positive and functionalized carbon fiber thread (FCFT) as negative electrode, coated with PVA‐H3PO4 gel electrolyte and twisted together	High operating voltage (1.6 V).	2 mWh cm^−3^	11 W cm^−3^		Almost unchanged at a strain of 100%	[372]

2D shaped							
Substrate (Reporting year)	Device Configuration	Device capacitance	Energy density	Power density	Capacitance retention	Flexibility	Refs.
Cotton fabric (2013)	Poly(pyrrole)‐coated cotton fabrics electrode, prepared in mixed surfactants: cetyltrimethylammoni‐um bromide (CTAB) and sodium dodecyl benzene sulfonate (SDBS), with NaCl solution	51.7 mAh g^−1^			Negligible decay after 100 cycles		[373]
Cotton fabric (2013)	In situ oxidation polymerization of pyrrole in the presence of lignosulfonate as both template and dopant to prepare PPy/ lignosulfonate (PPy/LGS) coated cotton fabric electrode	304 F g^−1^ at 0.1 A g^−1^					[374]
Cotton fabric (2015)	PPy nanorods deposited on cotton fabrics via in situ polymerization	325 F g^−1^	24.7 Wh kg^−1^ at 0.6 mA cm−2		200 F g^−1^ after 500 cycles		[375]
Cotton fabric (2016)	Polypyrrole, PPy coated cotton as working, Pt sheet as counter, Ag/AgCl as reference electrode with 1 m H_2_SO_4_ as electrolyte	Knitted 4117, woven 2191, and nonwoven fabrics 2905 mF cm^−2^	5.94 Wh kg^−1^	259.55 W kg^−1^	Stable within 5000 cycles		[376]
Cotton woven and knit (2019)	In situ polymerization coating of PPy on fabric	Woven 1748, Knitted 4848 mF cm^−2^ at 1 mA cm^−2^			88% after 5000 cycles		[367]
Cotton knit fabric (2019)	In situ chemical polymerization of Polypyrrole on fabric with PVA‐H_2_SO_4_ gel electrolyte	Electrode 481 and 1433 mFcm^−2^ at 5 mV s^−1^ and 1 mAcm^−2^ respectively, device 101 and 450 mFcm^−2^ at 5 mV s^−1^ and 1 mAcm^−2^ respectively	0.4 Whm^−2^ (2.3 W h kg^−1^ based on total mass of 2 electrodes)	10 W m^−2^ (57.5 W kg^−1^)	30% after 500 cycles (gel electrolyte), above 53% at 5000th cycle (aqueous electrolyte)	Electrode ≈78%–91% after stretched 1000 times, device capacitance enhanced to 160% at 5 mA cm^−2^	[366]
Cotton knit fabric (2019)	PPy‐coated fabric electrodes via chemical polymerization technique with 1 m sulfuric acid aqueous electrolyte	Electrode 5073 mF cm^−2^ at 1 mA cm^−2^, device 1167.9 mF cm^−2^ at 1 mA cm^−2^ and 904.2 mF cm^−2^ at 20 mA cm^−2^	102.4 µWh cm^−2^ at 0.39 mW cm^−2^		90% capacitance after 2000 cycles		[368]
Nylon/lycra (80/20) knitted fabric (2012)	Synthesis of PPy by chemical polymerization on the fabric with 1.0 m NaCl electrolyte	123.3 F g^−1^ at a scan rate of 10 mV s^−1^	6.7 Wh kg^−1^	753.4 W kg^−1^		90% after being stretched to 100% for 1000 times	[377]
Polyester fabric (2018)	Repeated spray‐coating of PEDOT:PSS solutions containing 5 wt.% dimethyl sulfoxide (DMSO)	75.30 F g^−1^ at the scan rate of 20 mVs^−1^					[378]
Polyester knitted fabric (2019)	In situ polymerization coating of PPy on fabric	1213 mF cm^−2^ at 1 mA cm^−2^					[367]
Polyester fabric (2020)	Electrospun poly(3,4‐ethylene dioxythiophene) polystyrene sulfonate (PEDOT: PSS) nanofibers were deposited onto flexible polyethylene terephthalate (PET) substrates to obtain electrodes with PVA/H_3_PO_4_ gel polyelectrolyte	1.8 m F cm^−2^ and 3.6 F g^−1^ at 5 µA cm^−2^	0.32 Whkg^−1^ at 5 µA cm^−2^	11.8 Wkg^−1^	92% after 1000 cycles		[369]
Polyester fabric (2021)	Conductive polyester (PET) fabric electrode is prepared by in situ polymerizations of aniline and pyrrole.	Electrode 1046 mF cm^−2^ at 2 mA cm^−2^ (aniline to pyrrole is 0.75:0.25), Device 537 mFcm^−2^, 1.13 F cm^−3^at 2 mA cm^−2^	0.043 mWh cm^−3^	0.005 Wcm^−3^	54.2% after 1000 cycles.		[370]
Cotton/polyester (55/45) fabric (2020)	Dimethyl sulfoxide (DMSO)‐doped PEDOT:PSS‐coated cloth as an active electrode and sweat as an electrolyte	Artificial sweat (7.64 F g^−1^ in terms of weight and 8.45 mF cm^−2^ in terms of area at the low current density, 0.07 A g^−1^) and real human sweat (3.88 F g^−1^)	Artificial sweat 1.36 Wh kg^−1^ (1.63 µWh cm^−2^). With real human sweat 0.25 Wh kg^−1^	Artificial sweat 329.70 W kg^−1^ (0.40 mW cm^−2^), With real human sweat 30.62 W kg^−1^	75% after 4000 cycles, 45% after 5000 cycles		[149]
Polypropylene (PP) non‐woven textile (2021)	Reactive inkjet printing to fabricate PPy layers on textile substrates with direct freezing of inks	72.3 F g^−1^ at 0.6 A g^−1^ at −12 °C	6.12 Wh kg^−1^	139 W kg^−1^	55.4% after 2000 cycles		[254]
Silk woven fabric (2019)	In situ polymerization coating of PPy on fabric	1349 mF cm^−2^ at 1 mA cm^−2^					[367]
Wool gauze fabric (2019)	In situ polymerization coating of PPy on fabric	1007 mF cm^−2^ at 1 mA cm^−2^					[367]
Fiberglass cloth (2020)	Conductive fiberglass cloth (CFC) derived from gas‐phase polymerization of pyrrole, followed by electrochemical polymerization of a layer of PPy attached to the surface of the conductive fiberglass cloth. SC formed by sandwiching two PPy/CFC composites and a layer of PVA‐H_2_SO_4_ gel electrolyte	549.6 mF cm^−2^	48.85 µWh cm^−2^		92.4% after 10, 000 cycles	96.08% after 1000 bending cycles	[379]
Carbon fabric (2010)	PEDOT nanofiber electrode, carbon cloths as the current collectors, and electrospun PAN nanofibrous membranes as the separator with ionic liquid electrolyte	20 Fg^−1^			90% after 10 000 cycles		[380]
CNT fiber woven textiles (2014)	Two PANI deposited CNT fiber textile stacked with PVA‐H_3_PO_4_ gel electrolyte	272.7 Fg^−1^ at 1 Ag^−1^			No decay after 2000 cycles	96.4% after 200 cycles bending at 150°	[381]

However, several works^[^ ^366^, ^367^, ^368^ ^]^ have been reported on 2D fabric‐shaped SCs. For instance, Wang et al.^[^ ^366^ ^]^ prepared flexible and stretchable electrodes via in situ polymerization of conducting PPy polymers on knitted cotton fabrics. The areal capacitances of symmetric all‐solid‐state SC based on those electrodes were found to be 101 and 450 mF cm^−2^ at 5 mV s^−1^ and 1 mA cm^−2^, respectively. The capacitance retention of such devices was reported at 53% after 5000 charge‐discharge cycles. In another study by Lv et al.,^[^ ^367^ ^]^ the electrochemical performances of several woven and knitted fabrics‐based electrodes of cotton, wool, silk, and polyester fibers were enhanced via an improved in situ polymerization method. The conjugate length of the PPy molecule and doping levels were improved to provide a thin and dense conductive polymer coating on the fabric surface with a sheet resistance <10 Ω sq^−1^. The highest specific capacitance of 4848 mF cm^−2^ at 1 mA cm^−2^ was reported for PPy‐coated knitted electrodes of cotton fibers, with a capacitance retention of 88% after 5000 cycles, which is 35% higher than the previously reported work. The electrical conductivity of electrodes was also found to be almost unchanged even after washing in dichloromethane up to 20 laundering cycles.^[^ ^367^ ^]^ The same research group further improved the capacitive performance of knitted electrodes of cotton fibers using an improved chemical polymerization technique.^[^ ^368^ ^]^ The PPy‐coated fabric electrode showed a superior specific areal capacitance of 5073 mF cm^−2^, and the fabric‐based symmetric all‐solid‐state SC exhibited an enhanced specific areal capacitance of 1167.9 mF cm^−2^ at 1 mA cm^−2^ which are highest among conductive polymer‐based textile SCs of cotton fibers. Additionally, the same device provided a very high energy density of 102.4 µWh cm^−2^ at a power density of 0.39 mW cm^−2^ which maintained ≈90% capacitance after 2000 cycles.

Few researchers also investigated polyester fabric‐based electrodes for fabricating textiles SCs. Cárdenas‐Martínez et al.^[^ ^369^ ^]^ deposited electro‐spun PEDOT: PSS nanofibers on flexible polyester textiles. The all solid‐state SC exhibited an areal capacitance of 1.8 mF cm^−2^ and gravimetric capacitances of 3.6 Fg^−1^ at a discharge current of 5 µA cm^−2^. However, like cotton textiles, in situ polymerization of PPy resulted in better capacitive performances for polyester fabrics. Such PPy coating on polyester fabrics exhibited an areal capacitance of 1213 mF cm^−2^ at 1 mA cm^−2^.^[^ ^367^ ^]^ Additionally, an in situ polymerization of aniline and pyrrole on polyester (PET) was reported by Xie et al.^[^ ^370^ ^]^ to produce SC electrodes. The electrode exhibited an areal capacitance of 1046 mF cm^−2^ at 2 mA cm^−2^ when the monomer ratio of aniline to pyrrole was 0.75:0.25. The SC device showed areal capacitance of 537 mF cm^−2^, volumetric capacitance of 1.13 F cm^−3^ at 2 mA cm^−2^ with energy and power densities of 0.043 mWhcm^−3^ and 0.005 Wcm^−3^, respectively. In addition to cotton and polyester, Lv et al.^[^ ^367^ ^]^ also used protein fibers as SC substrate. The in situ polymerization of PPy exhibited an areal capacitance of 1349 mFcm^−2^ in case of silk and 1007 mFcm^−2^ in case of wool gauze fabric at 1 mA cm^−2^. Few other research groups also investigated carbon fabric as SC substrates. The highest gravimetric capacitance on carbon cloth was reported by Wang et al.^[^ ^371^ ^]^ They drop casted polyaniline on functionalized carbon cloth, the SC showed a gravimetric capacitance of 319.5 Fg^−1^ at 0.2 A g^−1^ with cyclic stability up to 82% after 1000 charge‐discharge cycle.

### 6.3 Metal‐Based Textiles Supercapacitors

Distinguished by particular physical and chemical properties, metal and metal oxide materials have been a focus of research and exploitation for applications in SCs,^[^ ^382^ ^]^ **Table** **10**. CNTs were studied mostly as substrate for the fabrication of metal‐based 1D fiber or yarn‐shaped SCs. Su et al.^[^ ^383^ ^]^ produced high‐performance asymmetric two‐ply yarn SC from spun CNT yarn (as negative electrode) and CNT@MnO_2_ composite yarn (as positive electrode) in aqueous electrolyte. This asymmetric architecture resulted in areal capacitance of 12.5 Fg^−1^ with higher energy and power densities compared to the reference symmetric two‐ply yarn SCs, 42.0 Whkg^−1^ at a lower power density of 483.7 Wkg^−1^, and 28.02 Whkg^−1^ at a higher power density of 19,250 W kg^−1^. Co_3_O_4_ was also studied by several research groups for enhancing capacitive performance. Abouali et al.^[^ ^384^ ^]^ employed a facile electrospinning method with subsequent heat treatments to prepare carbon nanofibers (CNFs) electrodes for SCs. The electrodes possessed a remarkable capacitance of 586 F g^−1^ at a current density of 1 Ag^−1^ with excellent cyclic stability of 74% upto 2000 cycles at 2 A g^−1^. Su et al.^[^ ^385^ ^]^ in another study, further compared the electrochemical performance of CNT yarn electrodeposited with NiO along with Co_3_O_4_. The two‐ply SCs formed from CNT@Co_3_O_4_ composite yarns displayed excellent electrochemical properties with high capacitance of 52.6 mF cm^−2^ and energy density of 1.10 µWh cm^−2^.

**Table 10:** Summary of Metal‐based supercapacitors or supercapacitor electrodes

1D shaped							
Substrate (Reporting year)	Device Configuration	Device capacitance	Energy density	Power density	Capacitance retention	Flexibility	Refs.
Kevlar fiber (2011)	Kevlar fibers and flexible plastic wire substrates for ZnO NW arrays, Plastic wire/ZnO, Kevlar/ZnO/Au electrodes with KNO_3_ and PVA‐H_3_PO_4_ electrolyte	0.21 mF cm^−2^ at 100 mV s^−1^ (aqueous) and 2.4 mF cm^−2^ and 0.2 mF cm^−1^ (gel electrolyte)	2.7×10^−8^ Wh cm^−2^ (gel)	1.4×10^−5^ Wcm^−2^ (gel)			[389]
Carbon nanotube (CNT) yarn (2014)	CNT||CNT, CNT@MnO_2_||CNT, CNT@MnO_2_|| CNT@MnO_2_ with aqueous electrolyte	Asymmetric SC CNT@MnO_2_ (positive) and CNT (negative) possess a capacitance of 12.5 F g^−1^ at a current density of 0.14 A g^−1^	1 to 2.12 Wh kg^−1^ (CNT||CNT), For CNT@MnO_2_ || CNT, up to 42.0 Wh kg^−1^ (low power density), and 28.02 Wh kg^−1^ (at high power density)	241.8 to 10 000 W kg^−1^ (CNT||CNT), for CNT@MnO_2_ || CNT, low power density of 483.7 W kg^−1^, high power density of 19 250 W kg^−1^	For CNT@MnO_2_ || CNT 98% of its original capacitance after 500 cycles, in comparison with 99% for CNT||CNT	For CNT@MnO_2_|| CNT, specific capacitance suffered only 0.5% reduction after 200 cycles of folding and unfolding actions	[383]
Carbon nanotube (CNT) yarn (2015)	NiO and Co_3_O_4_ deposited on spun CNT yarn, Two PVA‐H_2_SO_4_ coated CNT, CNT@NiO, and CNT@Co_3_O_4_ yarns placed together and coated with electrolyte again	CNT@Co_3_O_4_ yarn based SC 52.6 mF cm^−2^ at 0.053 mA cm^−2^, 87.6 Ag^−1^, CNT based SC 7.4 mF cm^−2^, 13.4 Ag^−1^, CNT@NiO based SC 15.2 mF cm^−2^, 25.9 Ag^−1^	CNT@Co_3_O_4_ yarn based SC 1.1 µWh cm^−2^	CNT@Co_3_O_4_ yarn based SC 0.01 mW cm^−2^	Pure CNT, CNT@NiO, and CNT@Co_3_O_4_ maintain 96%, 94%, and 91%, respectively, of original capacitance after 1000 cycles	No decrease after 100 cycles bending at 90° and 180°	[385]
Carbon nanofiber (CNF) (2015)	CNFs containing Co_3_O_4_ nanoparticles electrodes with 6 m KOH	586 Fg^−1^ at 1 Ag^−1^			74% after 2000 cycles		[384]

2D shaped							
Substrate (Reporting year)	Device Configuration	Device capacitance	Energy density	Power density	Capacitance retention	Flexibility	Refs.
Polyester (2020)	Ni nanoparticle in situ synthesized, two Ni‐plated polyester electrodes with PVA/KOH gel electrolyte	450 mF cm^−2^ at 7.5 mA cm^−2^					[386]
Silk fabric (2021)	Graphite coated Berlin (silver coated silk) as the negative electrode and Nora Dell (Ni/Cu/Ag coated) fabric as the positive electrode with biocompatible PVA‐KCl gel electrolyte	32 mF cm^−2^ at 25 mV s^−1^, 41 mF cm^−2^ at 0.75 mA cm^−2^	2.8 µ Wh cm^−2^ at 25 mV s^−1^, 3.6 µ Wh cm^−2^ at 0.75 mA cm^−2^		45% retention after 1000 cycles		[390]
Carbon cloth (2013)	Hydrogenated single‐crystal ZnO @ amorphous ZnO‐doped MnO_2_ core‐shell nanocables (HZM) on carbon cloth as SC electrodes using PVA/LiCl electrolyte	26 mFcm^−2^ (325 mF cm^−3^) at a current density of 0.5 mA cm^−2^	0.04 mWh cm^−3^	2.44 mW cm^−3^	87.5% after 10 000 charge/discharge cycles		[391]
Carbon cloth (2015)	Electrochemically activated carbon cloth (EACC) as anode and TiN@MnO_2_ on CC as cathode with LiCl solution as electrolyte.	2.69 F cm^−3^ at 6 mA cm^−2^	1.5 mWh cm^−3^	1.71 W cm^−3^	No decay at 70 000 cycles		[338]
Carbon fabric (2016)	uniform large‐area manganese oxide (MnO_2_) nanosheets on carbon fabric which oxidized using O_2_ plasma treatment (MnO2/O2‐carbon fabric) via electrodeposition process with 1 M sodium sulfate (Na_2_SO_4_) electrolyte	275 Fg^−1^ at a scan rate of 5 mVs^−1^			80% after 10 000 cycles.		[392]
Carbon cloth (2016)	In situ electrodeposition of MnO2 on carbon cloth with 1 m Na_2_SO_4_ aqueous electrolyte	275 F g^−1^ at 0.2 A g^−1^					[393]
Carbon textile (2016)	Zinc sulfide (ZnS) nanospheres hydrothermally grown on flexible carbon textile (CT)	540 F g^−1^ (56.25 F cm^−2^) at 5 mV s^−1^	51 W h kg^−1^ at a power density of 205 W kg^−1^		94.6% after 5000 cycles		[387]
Carbon fiber fabric (2017)	KOH activation of commercial CF threads followed by coated with PVA/ H_3_PO_4_ electrolyte and woven to SC fabric	18.6 F g^−1^ at 2 mV s^−1^, 300 mF cm^−2^, 26 F cm^−3^	2.58 mWh g^−1^ or 3.6 mWh cm^−3^, 42 µWh cm^−2^, 3.6 mWh cm^−3^		Over 80% after 10 000 at 20 mV s^−1^		[394]
Carbon fabric (2017)	Fabrication of Co_3_O_4_ nanowires on a flexible carbon fabric electrode (CoNW/CF) with PVA‐KOH gel electrolyte	Electrode 3290 F g^−1^ at a scan rate of 5 mV s^−1^	6.7 Wh kg^−1^	5 kW kg^−1^	95.3% after 5000 cycles		[388]
Carbon cloth (2018)	Anodic deposition of MnOx on Pd coated carbon cloth electrode with 0.5 m Na_2_SO_4_	186 F g^−1^ at 1 mA cm^−2^					[395]
Carbon fiber fabric (2020)	Viscose fiber woven fabrics carbonized and activated (ACVF), CeO_2_, ZnO hydrothermally deposited, CeO_2_‐ACVF & ZnO‐ACVF electrode, polyester separator, carbon cloth current collector with PVA/H_3_PO_4_ electrolyte	13.24 mF cm^−2^ at 0.2 mVs^−1^	4.6×10^−7^ Whcm^−2^ at 3.31×10^−6^ Wcm^−2^		87.6% at 5000 cycles	Increases by 13.4% after 200 bending cycles	[396]
Carbon fiber textile (2021)	Crystalline nano‐flowers structured zinc oxide (ZnO) was directly grown on carbon fiber textile (CFT) substrate via hydrothermal process and fabricated with a binder‐free electrode (denoted as ZnO@CFT) for SC	201 Fg^−1^ at 1 Ag^−1^			90.32% after 3000 cycles at 10 Ag^−1^		[397]

Among conventional textiles, polyester and silk were studied for 2D fabric‐shaped SC. Shahidi et al.^[^ ^386^ ^]^ deposited Ni nanoparticles on polyester to prepare flexible electrodes for SC application. The device achieved a high areal capacitance of 450 mF cm^−2^ at 7.5 mA cm^−2^. Similar to 1D type, carbon fabrics were investigated mostly for the 2D fabric‐shaped textile SC. Javed et al.^[^ ^387^ ^]^ hydrothermally grew zinc sulfide (ZnS) nanospheres on a flexible carbon textile. The flexible and lightweight electrode exhibited a higher capacitance of 747 F g^−1^ at a scan rate of 5 mV s^−1^ in the LiCl aqueous electrolyte. The SC device demonstrated specific capacitance of 540 F g^−1^ and areal capacitance of 56.25 F cm^−2^ with excellent cycling stability of 94.6% after 5000 cycles. The energy density was reported as high as 51 W h kg^−1^ at a power density of 205 W kg^−1^. However, the highest capacitance for the metal‐based textile SC was achieved utilizing Co_3_O_4_. Howli et al.^[^ ^388^ ^]^ hydrothermally fabricated Co_3_O_4_ nanowires on carbon fabric substrate to form SC electrode. With PVA‐KOH electrolyte the electrode exhibited gravimetric capacitance of 3290 F g^−1^ at a scan rate of 5 mV s^−1^ with high energy and power densities of 6.7 Wh kg^−1^ and 5000 W kg^−1^, respectively, and capacitance retention of 95.3% after 5000 cycles.

### 6.4 Other 2D Material‐Based Textile Supercapacitor

Very recently several 2D materials are explored for the fabrication of SC electrodes. It is evident from the literature that carbon, and cotton textiles were mainly studied for 2D fabric‐shaped SCs, **Table** **11**. Uzun et al.^[^ ^398^ ^]^ coated cellulose yarns with Ti_3_C_2_T_x_ to produce conductive yarns. The yarn electrode exhibited linear, areal, and volumetric capacitance of ≈759.5 mF cm^−1^, ≈3965.0 mF cm^−2^, and ≈260.0 mF cm^−3^ respectively at 2 mV s^−1^. With PVA–H_2_SO_4_ gel electrolyte, the SC device showed linear, areal, and volumetric capacitance of ≈306.9 mF cm^−1^, ≈1865.3 mF cm^−2^, and ≈142.4 mF cm^−3^ respectively. Levitt et al.^[^ ^307^ ^]^ coated cotton yarn with Ti_3_C_2_T_x_ (referred to as MXene), knitted the yarn 3D, and used 1 m H_3_PO_4_‐PVA gel electrolyte for analyzing the structure performance as SC. The areal capacitance was reported at 519 mF cm^−2^ at 2 mV s^−1^. They further replaced the electrolyte with 1 m H_3_PO_4_ electrolyte and the capacitance increased up to 707 mF cm^−2^ at 2 mV s^−1^. The device showed >100% capacitance over 10 000 charge‐discharge cycles and coulombic efficiency of ≈100%.

**Table 11:** Summary of other 2d material‐based supercapacitors or supercapacitor electrodes

1D shaped							
Substrate (Reporting year)	Device Configuration	Device capacitance	Energy density	Power density	Capacitance retention	Flexibility	Refs.
Cotton yarn (2019)	Coating cellulose yarns with Ti_3_C_2_T_x_ MXene with 1 m H2SO4 electrolyte	Electrode ≈759.5 mF cm^−1^, ≈3965.0 mF cm^−2^ and ≈260.0 mF cm^−3^ at 2 mV s^−1^. Device ≈306.9 mF cm^−1^, ≈1865.3 mF cm^−2^, ≈142.4 mF cm^−3^ at 2 mV s^−1^)			≈100% after 10 000 cycles		[398]

2D shaped							
Substrate (Reporting year)	Device Configuration	Device capacitance	Energy density	Power density	Capacitance retention	Flexibility	Refs.
Cotton fabric (2018)	Dipping and drying of cotton into MXene (Ti_3_C_2_T_x_) nanosheets	182.70 F g^−1^					[399]
Cotton fabric (2020)	3D knitted cotton yarn coated with Ti_3_C_2_T_x_ (referred as MXene) and 1 m H_3_PO_4_/PVA gel electrolyte	519 mF cm^−2^ at 2 mV s^−1^, (707 mF cm^−2^ at 2 mV s^−1^ with 1 m H_3_PO_4_ electrolyte)	25.4 µWh cm^−2^	0.47 mWcm^−2^	>100% over 10 000 cycles		[307]
Carbon cloth (2018)	Hydrothermal growth of molybdenum disulfide (MoS_2_) nanosheets on carbon fabrics, with Lithium metal counter electrode, and a 1.0 m LiPF6 solution with mixture of ethylene carbonate and dimethyl carbonate (EC/DMC, 1:1 in volume) electrolyte	159.38 mF cm^−2^ at 0.5 mA cm^−2^			80.6% after 15 000 cycles		[400]
Carbon fabric (2018)	Stacking the RuO_2_ coated carbon fabric (CF) as positive and Ti_3_C_2_T_x_ coated CF as negative electrode with 1 m H_2_SO_4_ electrolyte	Electrode 416 mF cm^−2^, 200 F g^−1^	Device 37 µWh cm^−2^ at a power density of 40 mW cm^−2^		86% after 20 000 cycles		[401]
Carbon cloth (2021)	Hierarchical flower‐like Mn_3_O_4_@N, P‐doped carbon (NPC) composite cathode with an electrochemically reduced porous carbon (PC) anode and a PVA‐Na_2_SO_4_ hydrogel electrolyte	81.97 Fg^−1^ at 1 A g^−1^	76.96 Wh kg^−1^	26.02 kW kg^−1^ at 32.65 Wh kg^−1^	92.71% after 10 000 cycles		[402]
Carbon cloth (2021)	MoS_2_ drop cast on functionalized carbon cloth; MoS_2_/FCC electrodes soaked with 1 m H_2_SO_4_ electrolyte	56.525 F g^−1^ at 0.2 A g^−1^			29% after 1000 cycles		[371]

### 6.5 Hybrid Materials

The hybridization of the active materials from one or more subgroups (e.g., carbonaceous compounds, conductive polymers, metal‐based, and other 2D materials) is one of the attractive routes to fabricate high‐performance energy storage textiles, **Table** **12**. Several research groups have focused on hybridizing carbonaceous compounds with conductive polymers or metal oxides to prepare 1D fiber or yarn‐shaped SC. For example, several articles reported cotton fiber or yarn‐based SC devices as hybrid active compounds. Liu et al.^[^ ^403^ ^]^ reported a fully cable‐type SC composed of two PPy‐MnO_2_‐CNT‐cotton thread (CT) electrodes, separated by cotton textiles wrapped with 0.5 m Na_2_SO_4_ electrolyte in a transparent silicone pipeline package shell. The CTs were coated with SWCNT, followed by electrochemical deposition of MnO_2_ nanostructures and PPy film. The resulting electrodes achieved a high areal capacitance of 1490 mF cm^−2^ at a scan rate of 1 mVs^−1^, which is one of the highest among cotton fiber‐based SC electrodes. The device prepared from such electrode achieved an energy density of 33 µWh cm^−2^ at a power density of 0.67 mWcm^−2^. In another study,^[^ ^404^ ^]^ almost similar capacitive performances were obtained by Wang et al. with considerably higher energy and power densities. They modified cotton yarns with 2D metallic Ni conductive network and pseudocapacitive Co—Ni layered double hydroxide nanosheet array. The flexible yarn electrodes achieved a areal capacitance of 1260 mF cm^−2^ (121 571.1 C cm^−2^) at a scan rate of 5 mV s^−1^. The SC device was prepared by twisting two as‐made yarn together, and then painted with PVA/KOH gel electrolyte, which provided an areal capacitance of 221 mFcm^−2^ (21 323.2 C cm^−2^) at 0.04 mA cm^−2^. The energy and power densities were reported as 9.3 mWh cm^−2^ and 43.99 mW cm^−2^, respectively.

**Table 12:** Summary of Hybrid material‐based supercapacitors or supercapacitor electrodes

1D shaped							
Substrate (Reporting year)	Device Configuration	Device capacitance	Energy density	Power density	Capacitance retention	Flexibility	Refs.
Cotton thread (2013)	Two PPy‐MnO_2_ ‐CNT‐cotton thread electrodes, separated with cotton textile wrapping with 0.5 m Na_2_SO_4_ electrolyte, transparent silicone pipeline as a package shell, and a fully cable‐type SC	1.49 Fcm^−2^ at scan rate 1 mVs^−1^ (electrode)	33 µ Wh cm^−2^ at power density 0.67 mWcm^−2^	13 mW cm^−2^ at an energy density of 14.7 µWhcm^−2^	87% after 2000 cycles		[403]
Cellulose yarns (cotton, linen, bamboo, viscose) (2015)	Yarns welded with activated carbon and twisting with stainless steel yarn	120 F g^−1^, 37 mF cm^−1^ at 2 mV s^−1^			77% after 3000 cycles	Some decay at 180° bent, curled, and crumpled	[428]
Cotton yarn (2015)	Electroless deposition of Ni and electrochemical deposition of graphene on commercial cotton yarns (RGO/Ni cotton composite electrodes) with PVA/LiCl gel, as electrolyte and separator	0.11 F cm^−1^	6.1 mWh cm^−3^	1400 mW cm^−3^	82% after 10 000 cycles	No decrease at 45°, 90°, and 180° bending, 95% after 4000 cycles at 180°	[429]
Cotton thread (2016)	Twisting 2 strands of PVA‐H_3_PO_4_ gel electrolyte coated carbon nanoparticles /rGO‐cotton thread (CNPs/rGO‐CT) together	3.79 mF cm^−3^ at 50 mVs^−1^	0.084 µWhcm^−3^ (with 35.3 µWcm^−3^), Varies from 0.084 µWhcm^−3^ to 0.047 µWhcm^−3^ (in the power density range of 35.3 µWcm^−3^ to 56.4 µWcm^−3^)		95.23% after 10 000 cycles	92.30% after 2000 bending cycles	[430]
Cotton thread (2016)	Twisting 2 strands of PVA‐H_3_PO_4_ gel electrolyte coated graphene hydrogels/multi‐walled carbon nanotubes‐cotton thread (GHs/MWCNTs‐CT)	97.73 µFcm^−1^ at a scan rate of 2 mV s^−1^	4.79 × 10^−3^ mWh cm^−1^ (with power density 0.75 mWcm^−1^)	1.25 mWcm^−1^ (with energy density 3.06 × 10^−3^ mWh cm^−1^)	95.51% after 8000 cycles	90.75% after 500 continuous bending cycles	[430]
Cotton thread (2018)	Two cotton/Ni/Co—Ni layered double hydroxide (CT/Ni/Co—Ni LDH) hybrid yarn electrodes twisted together and painted with PVA/KOH gel electrolyte	Electrode material 1.26 Fcm^−2^ (121 571.1 C cm^−2^) at a scan rate of 5 mV s^−1^, SC 221 mFcm^−2^ (21 323.2 C cm^−2^) at 0.04 mA cm^−2^	9.3 mWh cm^−2^	43.99 mW cm^−2^	79% after 2000 cycles		[404]
Cotton yarn (2018)	Cotton electrode by dip‐dried in MWCNT followed by interfacial polymerization of PPy, two parallel electrodes embedded in a thin layer of PVA/H_3_PO_4_ layer	30 F g^−1^	2.63 mW h g^−1^	11.33 mW g^−1^			[431]
Cotton fibers (2018)	Short‐staple length stainless steel fibers (SSFs) blended with cotton fibers to spin SSF/cotton blended yarn, PPy deposited on PEDOT:PSS coated composite yarn, followed by coating with PVA/H_3_PO_4_ electrolyte, placed in parallel and twisted together, followed by coating again to produce a solid‐state two‐ply nanocomposite yarn SC.	1.36 F cm^−2^	0.16 mWh cm^−2^		80% over 5000 cycles		[405]
Cotton (2019)	GO nanosheets (NSs) are modified with ultrathin and large area MoS2 NSs followed by reduction with PVA‐H_3_PO_4_ electrolyte to produce all‐solid‐state hybrid fiber shaped SC (ASFS). 3 symmetrically assembled tandem and parallel ASFS groups in cotton textile (ASFSs‐T) fabricated	134.38 F g^−1^, 332.85 mF cm^−2^, and 221.9 F cm^−3^ at a current of 50 mA				100% when bent by 30 and 60 degrees	[407]
Cotton fiber (2021)	In situ growth of PPy and MXene composite, on cotton fiber to prepare fiber electrode	506.6 F g^−1^, at 1 A g^−1^ and 455.9 mF cm^−2^ at 0.9 mA cm^−2^			83.3% after 2000 cycles		[406]
Polyester fiber (2018)	PPy electrochemically deposited on rGO painted SnCl_2_ modified polyester yarn electrode with PVA/H_2_SO_4_ gel electrolyte for SC	Electrode 175.7 mF cm^−1^, 699.6 mF cm^−2^, 239.6 F g^−1^, 35.0 Fcm^−3^ at 0.13 mA cm^−1^, Device 85.3 mFcm^−1^, 339.7 mF cm^−2^, 116.4 Fg^−1^, 17.0 F cm^−3^	0.0472 mWhcm^−2^	26.5 mWcm^−2^		Almost original electrochemical performance after 1000 bending cycles	[408]
Polyaniline fiber (2013)	Two polyaniline composite fibers incorporated with aligned multi‐walled carbon nanotubes (MWCNTs/PANI) twisted	274 Fg^−1^, 263 mF cm^−1^ at 2 A g^−1^			99% after 1000 cycles	>97% after 50 bending cycles	[409]
Nylon fiber (2015)	Carbon multiwalled nanotube (MWNT) helically wrapped around nylon fibers, followed by electrochemical deposition of MnO_2_. Two coiled MnO_2_/CNT/nylon fiber electrodes placed parallel and coated with PVA‐LiCl gel electrolyte	5.4 mF cm^−1^, 40.9 mF cm^−2^, 3.8 Fcm^−3^ at 10 mVs^−1^	2.6 µWh cm^−2^	66.9 µW cm^−2^		90.8% at 12% strain, 50% during large strain	[232]
Elastic fiber (2014)	Elastic fiber/CNTs/PANI	255.5 Fg^−1^, 0.19 mF cm^−1^ at 1 A g^−1^	12.75 Wh kg^−1^	1494 W kg^−1^	69% after 10 000 cycles	93.8% after 1000 cycles bending at 180°	[229]
Shape‐memory polyurethane (SMP) substrate (2015)	By wrapping aligned carbon nanotube (CNT) sheets onto a shape‐memory polyurethane (SMP) substrate as electrode, coated with PVA gel electrolyte followed by winding another layer of aligned CNTs as the outer electrode	24 Fg^−1^, 0.269 mF cm^−1^ and 42.3 mFcm^−3^			No loss after 12 000 cycles	No obvious decrease after 500 cycles of deformation	[230]
Urethane stretchable yarn (2016)	CNTs dipping and PPy electrodeposition on urethane elastic fiber core spun yarns (UY)	69 mF cm^− 2^				Nearly unchanged at a strain of 80%.	[410]
Stretchable substrate (2017)	Electrochemical activation of pristine carbon nanotube fibers (CNTF), coating of PEDOT:PSS followed by electrochemical deposition of MnO_2_ to form MnO_2_@PEDOT:PSS@OCNTF positive electrode and hydrothermal synthesis of MoS_2_ to form MoS_2_ @CNTF negative electrode with LiCl‐PVA gel electrolyte placed on a stretchable substrate	278.6 mF cm^−2^	125.37 µWh cm^−2^ at 540 µWcm^−2^			92% after stretching at a strain of 100% for 3000 cycles.	[411]
Carbon nanotube (CNT) yarn (2013)	PANI nanowire arrays in situ deposited on CNT yarn, PVA gel coated on CNT yarn, or CNT/PANI yarn. Two CNT yarns or composite yarns twisted together	At 0.01 mA cm^−2^ CNT/PANI yarn based SC 38 mFcm^−2^, pure CNT yarn‐based SC 2.3 mFcm^−2^. CNT/PANI yarn 12 mFcm^−2^ at 1 mAcm^−2^			91% after 800 cycles	No decrease at 45°, 90°, 135°, 180° bending	[432]
Carbon nanotube (CNT) yarn (2014)	Electrochemical deposition of MnO_2_ onto CNT yarn, Two PVA‐KOH coated CNT/MnO_2_ composite yarns placed on top of each other, and finally coated by PVA‐KOH again.	25.4 F cm^−3^ at 10 mV s^−1^	3.52 mWh cm^−3^	127 mW cm^−3^		No decrease after 1000 bending at 90°	[433]
Carbon nanotube CNT fiber (2017)	MnO nanosheets in situ grown on CNT fiber (positive), polyimide deposited on CNT fiber (negative electrode)		36.4 µWh cm^−2^ (At 0.78 mW cm^−2^)	15.6 mW cm^−2^ at 30.2 µWh cm^−2^	Well upto 2000 cycles	Specific capacitances remained almost unchanged at bending to various degrees (0–180)	[434]
Carbon nanotube (CNT) yarn (2017)	Twisting a number of CNT yarns (n) with Pt filament as current collector, PANI nanowires further deposited in situ to form the final Pt/n‐CNT @ PANI with alkaline electrolyte	Pt/5‐CNT@PANI FSSC 217.7 Fg^−1^, 48.27 mFcm^−3^ at current density 0.2 Ag^−1^	30.22 Wh kg^−1^ at 91.88 W kg^−1^, 19.31 Wh kg^−1^ at 9072.88 W kg^−1^		Negligible changes after 5000 cycles	Capacitance retention was 98.17% after 3000 cycles and 95.91% after 5000 flexing cycles	[435]
Carbon nanotube (CNT) fiber (2021)	Coating electroactive molybdenum disulfide (MoS_2_) nanoflakes on a CNT fiber backbone with rGO as the adhesion layer.	190.4 F g^−1^, 93.2 mFcm^−2^	26.4 Wh Kg^−1^	4000 Wkg^−1^	85% after 5000 cycles	No performance decay observed after 1000 cyclic bending	[436]
Carbon nanotube (CNT) and Polyaniline Nanowire (PANINW) composite yarn (2014)	Spun yarn composed of SWCNTs and PAniNWs, coated with PVA/H_2_SO_4_ gel electrolyte and twisted together	2.67 mF cm^−2^ at 0.6 A g^−1^	0.8 µWh cm^−2^	150 µW cm^−2^	86% after 800 cycles	No decrease at 45°, 90°, 135°, 180° bending	[437]
Carbon nanotube (CNT) /MXene yarn (2018)	Yarn electrodes by biscrolling MXene with CNTs, freestanding asymmetric yarn SC prototypes by pairing with biscrolled RuO_2_ yarns with 3.0 m H_2_SO_4_ electrolyte	3188 mF cm^−2^, 1083 F cm^−3^, 523 F g^−1^ at a current density of 2 mA cm^−2^	61.6 mWh cm^−3^ (168 µWh cm^−2^ and 8.4 µWh cm^−1^)	5428 mW cm^−3^ (14.8 mW cm^−2^ and 741 µW cm^−1^)	≈90% @10 000th cycle		[412]
Graphene fiber (2013)	2 intertwined hierarchical hybrid core–sheath fiber electrode [a core of graphene fiber (GF) covered with a sheath of 3D porous network‐like graphene framework, denoted as GF@3D‐G], solidified in the H_2_SO_4_‐PVA gel electrolyte.	1.7 mF cm^−2^, 25–40 F g^−1^, 20 µF cm^−1^ at 17 µA cm^−2^	0.4–1.7 × 10^−7^ Wh cm^−2^	6–100 × 10^−6^ W cm^−2^	Almost similar after 500 cycles	No decrease after bending 500 cycles	[438]
Graphene/PPy composite fiber (2014)	Intertwining two G/PPy electrodes pre‐coated with H_2_SO_4_–PVA gel polyelectrolyte	107 mF cm^−2^ at 0.24 mA cm^−2^	9.7 µWh cm^−2^			No decrease after 1000 bending cycles	[439]
GO/MXene (≈88 wt.%) fiber (2017)	Wet spinning of GO liquid crystal‐assisted MXene fiber with 1 m H_2_SO_4_ electrolyte	233 mF cm^−2^, 257 F g^−1^, 341 F cm^−3^					[440]
rGO/MXene hybrid fiber (2020)	Electrolyte Mediated hybrid fiber made of rGO and MXene, assembled into fibers via wet spinning with PVA/H2SO4 electrolyte	550.96 mF cm^−2^ and 110.89 F g^−1^ at 20 mV s^−1^,	12 µWh cm^−2^ and 9.85 mWh cm^−3^ at 8.8 mW cm^−2^ and 7.1 W cm^−3^		85% @ 10 000 cycle		[413]
rGO/10 wt.% MoS_2_ composite fiber (2021)	Wet spun rGO/10 wt.% MoS_2_ composite fiber with PVA‐ H_2_SO_4_ electrolyte	185.3 mF cm^−2^	3.38 mW h cm^−3^ and 0.0936 W cm^−3^				[441]
rGO/20 wt.% MoS_2_ composite fiber (2021)	Wet spun rGO/20 wt.% MoS_2_ composite fiber with PVA‐ H_2_SO_4_ electrolyte	282.6 mF cm^−2^	4.92 mW h cm^−3^ and 0.051 W cm^−3^	87% after 1000 cycles			[441]
MXene (70%) /PEDOT:PSS hybrid fiber (2019)	Wet spun fibers using hybrid formulations of Ti_3_C_2_T_x_ MXene nanosheets and PEDOT:PSS with PVA/H_2_SO_4_ electrolyte	Electrode 676 mF cm^−2^, 258 F g^−1^, 615 F cm^−3^	≈7.13 Wh cm^−3^	≈8249 mW cm^−3^	≈95% after 10 000 cycle	96% when cyclically stretched to 100% strain	[414]
Carbon fiber tow (CFT) (2015)	Activation of pristine CFTs by oxidative exfoliation by KMnO_4_/H_2_SO_4_, annealing in air, and reduction by a mixture of hydrogen iodide (HI) and acetic acid (AcOH), Twisting two activated electrodes together after coating with PVA/H_3_PO_4_ gel electrolyte	2.55 F cm^−3^ at 10 mV s^−1^	0.35 mWh cm^−3^	3000 mW cm^−3^	91% after 10 000 cycles	No decrease after 1000 bending at 90°, 135° knotting	[442]
Carbon fiber (2015)	A manganese oxide nanosheet grown on carbon nanoparticle coated carbon fiber (CF@CNPs) and functionalized CF@CNPs are employed as the positive and negative electrodes respectively with LiCl‐PVA solid‐state electrolyte	5 Fcm^−3^ at 2 mA cm^−3^	2.1 mWh cm^−3^	10.22 W cm^−3^	81.2% after 10 000 cycles	No decrease after bending at 180°, 360°.	[443]
Carbon fiber (2013)	PPy deposition on MnO_2_ nanoflakes coated carbon fiber (CF/MnO_2_/PPy) hybrid structure. Two PPy‐MnO_2_‐CFs were fixed on a preservative film substrate and assembled into a SC by sandwiching PVA/H_3_PO_4_ membrane as separator and electrolyte between electrodes	69.3 F cm^−3^ at 0.1 A cm^−3^	6.16 mWh cm^−3^	0.04 W cm^−3^	86.7% after 1000 cycles	99.8% after rolling up	[415]
Carbon fiber (2016)	Carbon fiber bundle @ CNT‐NiCo(OH)x (CF@CNC) as positive and carbon fiber bundle @ activated carbon (CF@AC) as negative electrode, both immersed in PVA‐KOH gel electrolyte & dried, twisted together	111 mF cm^−2^ (64.0 mF cm^−1^)	33.0 µWh cm^−2^ 0.84 mWh cm^−3^	0.75 mW cm^−2^ 19.1 mW cm^−3^	103% after 8000 cycles	20% decay after 1000 bending times and 107% retention after 1000 twisting times	[444]
Carbon fiber (2017)	Braided carbon fiber electrodes coated with MWNT/V_2_O_5_ nanowires (NWs), a cellulose‐based separator, and an ionic‐liquid based electrolyte of [EMIM][TFSI]/LiCl/Al_2_O_3_ nanoparticles	10.6 mF cm^−2^ at 0.5 mA cm^−2^			Almost 100% after > 10 000 cycles	98.7% after 1000 bending cycles	[445]
Carbon fiber yarn (CFY) (2018)	Sheath‐core polyaniline nanowire array grown on aligned carbon nanofibers/ carbon fiber yarn electrode (CFY@CNFs@PANI NWA). Two fiber‐shaped electrodes parallelly placed on PET substrate and immersed in PVA/H_2_SO_4_ gel electrolyte	234 mF cm^−2^ at a current density of 0.1 mA cm^−2^	21.4 µWh cm^−2^	at 0.52 mW cm^−2^	90% after 8000 cycles		[446]
Carbon fiber (2019)	Dip‐coating of mixture of ionic liquid 1‐ethyl‐3‐methylimidazolium bis(trifluoromethylsulfonyl) imide ([EMIM][TFSI]), carbon nanotubes, and electro‐polymerization of PPy onto Au coated carbon fiber with propylene carbonate‐poly(methyl methacrylate)‐[EMIM][TFSI] gel electrolyte to form wire‐type SC	38.49 mF cm^−2^ at 0.6 mAcm^−2^	24.7 µWh cm^−2^	3.52 mW cm^−2^		99% after 100 bending cycles, 95.6% capacitance retention at 60 min in water	[447]
Carbon fiber (2020)	PEDOT:PSS‐ rGO drop coating and MnO2 electrodeposition on carbon fiber, MnO2/PEDOT:PSS‐rGO, and PEDOT:PSS‐rGO as positive and negative electrodes with Na_2_SO_4_‐CMC solid‐state electrolyte	Electrode 2.92 F cm^−2^ (194 F cm^−3^, 550 mF cm^−1^) at 5 mA cm^−2^	295 µWh cm^−2^ (19 mWh cm^−3^, 55 µWh cm^−1^)	2900 µWcm^−2^ (190 mW cm^−3^, 545 µWcm^−1^)	96% after 5000 cycles		[416]
Platinum wire (2013)	PEDOT/MWNT biscrolled yarn with Pt wire with liquid electrolyte (H_2_SO_4_) or, with 1 m H_2_SO_4_/PVA gel electrolyte.	At 0.01 Vs^−1^, ≈167 F cm^−3^ (liquid electrolyte), ≈180 F cm^−3^ (solid). At 1 V s^−1^ ≈147 F cm^−3^ (liquid) and ≈145 F cm^−3^ (solid). At 10 V s^−1^, 13 F cm^−3^ (liquid) and 10 F cm^−3^ (solid)	1.4 mWh cm^−3^ (Solid)	40 W cm^−3^ (Solid)	98% after 2000 cycles	98% after 2000 bending, 92% after 10 000 winding, 99% after 10 000 cycles woven into gloves	[448]
Platinum yarn (2014)	PANI nanowire solution coated on CNT wrapped Pt yarns (Pt wire/CNTs/PANI) with PVA‐H_3_PO_4_ electrolyte	86.2 Fg^−1^, 0.24 mF cm^−1^, 52.5 mF cm^−2^ at 5 mVs^−1^	35.27 Wh kg^−1^	10.69 kW kg^−1^		No decrease after folding/unfolding 1000 cycles	[80]
Au wire (2018)	Two wire electrodes prepared by layer‐by‐layer (LbL) assembly of multiwalled carbon nanotubes (MWCNTs), vanadium oxide (VO_x_), wetted with organic electrolyte of propylene carbonate (PC)−acetonitrile (ACN)−lithium perchlorate (LiClO_4_)−poly(methyl methacrylate) (PMMA) and twisted together	5.23 mF cm^−2^ at 0.2 mA cm^−2^	1.86 µ Whcm^−2^	8.5 mW cm^−2^	94% after 10 000 cycles		[417]
Stainless steel yarn (2015)	Two PPy‐wrapped Fe_3_O_4_ deposited SS magnetic electrodes coated with PVA‐H_3_PO_4_ gel electrolyte, dried, twisted, and coated with PU	61.4 mF cm^−2^ at 10 mV s^−1^			77% after 1000 cycles	Self‐healing 71.8% at 4 breakings & reconnecting	[299]
Stainless steel fiber yarn (2015)	PPy@MnO_2_@rGO‐deposited conductive yarns as both active materials and current collectors, two parallel yarn electrodes were coated with PVA/H_3_PO_4_ electrolyte	36.6 mF cm^−1^ & 486 mF cm^−2^ in aqueous Na_2_SO_4_ electrolyte (3‐electrode cell) or 31 mF cm^−1^ and 411 mF cm^−2^ in PVA / H_3_PO_4_ (2‐electrode cell)	SS SC 0.0092 mWh cm^−2^ & 1.1 mWh cm^−3^	1.33 mW cm^−2^, 160 mW cm^−3^	SS SC 92% over 4950 cycles	80% after 1000 cycles at 90 bending, 91% after 1000 cycles knotting, 103% after 1000 cycles twisting	[418]
Stainless steel spring (SSS) (2018)	In situ synthesis of hierarchical carbon tubular nanostructures (hCTNs) and conducting polyaniline (PANI) composites onto SSS substrate to form stretchable SC electrode	277.8 F g^−1^ at 1 A g^−1^, and 402.8 mF cm^−1^ at 1 mA cm^−1^			75% over 3000 cycles	100% stretchable	[449]
Stainless steel filament (2020)	Core‐sheath single yarn was produced by PVA‐H_2_SO_4_ electrolyte‐mediated rGO & MXene fibers twisted around the SS filament core. Two‐ply yarns made from plying of two single core‐sheath yarns. Two yarn electrodes plied to obtain the ultimate YSCs.	Dual‐core YSC 253.01 mF cm^−2^ (43.6 mF cm^−1^ at 20 mV s^−1^)	27.1 µWh cm^−2^ at a power density of 2502.6 µW cm^−2^	2502.6 µWcm^−2^ at 27.1 µWh cm^−2^, 510.9 µWcm^−1^ 5.5 µWh cm^−1^	18% deterioration after 10 000 cycles	90% after 1000 bending cycles	[450]
Stainless steel filament (2020)	Hydrothermal deposition of nickel‐cobalt oxide on stainless steel cables, stainless steel cables @nickel‐cobalt oxide electrode	113×10^−3^ mAh cm^−1^ at 0.3 mA cm^−1^, 449×10^−3^ mAh cm^−2^ at 1.2 mA cm^−2^, 69 mAh cm^−3^ at 1.0 A cm^−3^ and linear density capacitance with 433×10^−3^ mAh N^−1^ and 0.22×10^−3^ mAh Tex^−1^ at 0.6 mA.					[451]

2D shaped							
Substrate (Reporting year)	Device Configuration	Device capacitance	Energy density	Power density	Capacitance retention	Flexibility	Refs.
Cotton fabric (2010)	Deposition of MnO_2_ after dipping and drying of cotton with single‐walled carbon nanotube (SWNT) ink electrodes with 2 m aqueous Li_2_SO_4_ electrolyte to form fabric SC	0.41 Fcm^−2^			Negligible change over 35 000 cycles		[223]
Non‐woven cloth (2011)	Dip coating of nonwoven wiper cloth in the SWCNT ink followed by deposition of PANI nanowire arrays to obtain the PANI/SWCNT/ cloth composite electrode,	390 F g^−1^ at 1 A g^−1^	26.6 Wh kg^−1^	7000 WKg^−1^	90% after 3000 cycles	No decrease at 90° bending	[452]
Cotton t‐shirt (2012)	MnO_2_/ ACT (activated carbon textiles from cotton t‐shirt) as positive, ACT as negative electrode, copper foil as current collectors, and Whatman filter paper soaked with 1 m Na_2_SO_4_ aqueous solution as the separator	120 Fg^−1^ at 1 mA cm^−2^	66.7 Wh kg^−1^	4.97 kW kg^−1^	97.5% after 1000 cycles		[453]
Cotton stretchable knitted fabric (90% cotton/10% Lycra) (2013)	Electrochemical deposition of polypyrrole on pre‐strained Au coated cotton fabric with 1.0 m NaCl	255 F g−1 at 10 mV s−1				Sustain up to 140% strain without electric failure	[454]
Cotton (2015)	Electrodes prepared by dip coating cotton on GO dispersion, thermal reduction, and polypyrrole deposition. SC device with 2.0 m NaCl aqueous solution electrolyte.	336 F g^−1^	21.1 Wh kg^−1^ at 0.6 mA cm^−2^				[455]
Cotton (2015)	PPy coated cotton fabric electrode through in situ chemical polymerization by using CuO nanoparticles as template, with 2.0 m NaCl aqueous solution	225 F g^−1^ at 0.6 mA cm^−2^			92% after 200 cycles		[456]
Cotton fabric (2015)	PPy conductive polymer coated on top of MnO_2_ nanoparticles deposited‐ CNT textile with PEO/Na_2_SO_4_‐based gel‐type electrolyte	461 F g^−1^	31.1 Wh kg^−1^	22.1 kW kg^−1^	93.8% over 10 000 cycles	98.5% upon 21% tensile strain, no capacity change upon 13% bending strain. 96.3% after imposing cyclic bending of 750 000 cycles	[457]
Cotton fabric (2015)	Binder‐free ternary composites of manganese oxide (MnO_2_) nanoparticles, SWNT, and conductive polymer PANI and (PEDOT:PSS) deposited layer‐by‐layer by dip coating to prepare cotton electrode with gel electrolyte composed of Acetonitrile (ACN): propylenecarbonate (PC): polymethyl methacrylate (PMMA): tetra‐butylammonium hexafluorophosphate (TBAPF6) in a ratio of 70:20:7:3 by weight	MnO_2_/SWNT/PANI: 294 Fg^−1^, MnO2/SWNT/PEDOT:PSS ternary composite devices: 246 Fg^−1^	MnO_2_/SWNT/PANI: 66.4 Whkg^−1^, MnO2/SWNT/PEDOT:PSS ternary composite devices: 60.2 Whkg^−1^	MnO_2_/SWNT/PANI: 746.5 Wkg^−1^, MnO2/SWNT/PEDOT:PSS ternary composite devices: 640.5 Wkg^−1^		<3%, down to a bending angle of 180°	[458]
Cotton fabric (2016)	Direct electrospinning of MWCNTs on nickel‐coated cotton fabrics (Ni–cotton)	973.5 mF cm^−2^ at 2.5 mA cm^−2^			No decay after 3000 cycles		[419]
Cotton fabric (2017)	CNT/rGO‐coated fabric as the negative electrode and PPy‐coated fabric as the positive electrode	570 mF cm^−2^ at 1 mA cm^−2^	0.26 mW hcm^−2^		91% after 1000 cycles	Excellent stability under bending 100 times with an angle of 180°.	[459]
Cotton fabric (2018)	PPy electrochemically deposited on the surface of dip‐dried cotton into MXene (Ti_3_C_2_T_x_) nanosheets with H_2_SO_4_/PVA solid electrolyte	Electrode (with 1 m H_2_SO_4_ solution) 343.20 F g^−1^	Device 1.30 mW h g^−1^	Device 41.1 mW g^−1^			[399]
Cotton fabric (2018)	PPy/TiO_2_‐coated cotton fabric electrode prepared via sol‐gel and in situ chemical oxidation method with 2.0 M NaCl aqueous was used as the electrolyte.	Electrode 733 F g^−1^ at 0.6 A cm^−2^	44.4 Wh kg^−1^ at a power density of 555 W kg^−1^				[460]
Cotton fabric (2020)	GO deposited by “dip and dry” method, chemically reduced into rGO/cotton fabric. MnO_2_ nanoparticles accumulated on rGO/cotton fabric by in situ chemical deposition, PANi layer coated on rGO/MnO_2_/cotton fabric by in situ oxidative polymerization technique with 1 m H_2_SO_4_ electrolyte solution	888 and 252 F g^−1^ at 1 and 25 A g^−1^, 444 Fcm^−2^			70% after 3000 cycles at 15 A g^−1^		[420]
Cotton fabric (2021)	PPy/rGO nanocomposite cotton fabric (NCF) by chemical polymerization,	PPy0.5/rGO NCF electrode 9300 mF cm^−2^ at 1 mA cm^−2^	Device 167 µWh cm^−2^	Device 1.20 mW cm^−2^	Electrode retention 94.47% after 10 000 cycles.		[461]
Cotton fabric (2022)	GO nanosheets fixed on the cotton fabric (CF) by vacuum filtration, pyrrole monomers and silver ions (Ag+) adsorbed on the surface of GO/CF by *π*‐*π* and electrostatic interactions, respectively to form flexible PPy/Ag/GO/CF electrodes with PVA/H_2_SO_4_ gel electrolyte	Electrode 1664.0 mF cm^−2^, 27.0 F cm^−3^, SC 286.6 mF cm^−2^ (4.7 F cm^−3^)	SC 25.5 µWh cm^−2^	SC 1149.5 µW cm^−2^		89.7% after 10 000 bending cycles	[462]
Bamboo fabric (2020)	MnO_2_–NiCo_2_O_4_ printed bamboo fabric as positive electrode, and rGO printed bamboo fabric as negative electrode with LiCl/PVA gel as solid‐state electrolyte	2.12 Fcm^−2^ (1766 F g^−1^) at 2 mA cm^−2^	37.8 mW cm^−3^	2678.4 mWcm^−3^	92% of after 5000 cycles		[255]
Polyester fabric (2011)	Graphene/MnO_2_‐textile as positive and SWNTs‐textile as negative electrode in aqueous Na_2_SO_4_ electrolyte	Hybrid graphene /MnO_2_‐based textiles, up to 315 F g^−1^	12.5 Wh kg^−1^	110 kW kg^−1^	95% after 5000 cycles		[224]
Polyester fabric (2011)	MnO_2_ nanoflowers electrodeposited onto carbon nanotube (CNT)‐enabled conductive textile fibers, MnO_2_‐CNT‐textile as positive electrode, reduced MnO_2_‐CNT‐textile as negative electrode, Whatman filter paper as separator, and 0.5 m Na_2_SO_4_ in water as electrolyte	410 Fg^−1^ at 5 mVs^−1^ and 2.8 F cm^−2^ at 0.05 mV s^−1^	≈5–20 Wh kg^−1^	≈13 000 W kg^−1^	80% after first 200 cycles, 60% after 10 000 and 50% after 50 000 cycles		[463]
Polyester film (2013)	Directly growing CNTs along graphene fibers with Fe_3_O_4_ nanoparticles for CVD of the nanotubes, PET films coated with Au layer as supporting substrates and current collectors, separator is a filter paper soaked with 1 m Na_2_SO_4_ aqueous electrolyte.	200.4 F g^−1^, 0.98 mF cm^−2^ at 20 mA cm^−2^				No decrease after 200 bending cycles	[464]
Polyester fabric (2014)	CNT dip‐coated onto Cu metalized PET fabrics electrode	8.5×10^−3^ F cm^−2^					[359]
Polyester fabric (2014)	PANI film deposited on the CNT/Au/PET; (CNT dip‐coated onto Au metalized PET fabrics) to fabricate a solid‐state flexible CNT SC with PVA/H_3_PO_4_ electrolyte	7.1m F cm^−2^ (electrode) Specific capacitance (CV) 0.51 F cm^−2^, GCD 0.103 F cm^−2^ at current density of 1m A cm^−2^	2.412×10^−4^ W h cm^−3^ with 0.011 W cm^−3^		89% after 2500 cycles	excellent performance under bending at 45°, 90°, 135°	[359]
Polyester fabric (2015)	Strip‐shaped electrode prepared by PANI deposited on aligned CNTs, with PVA/H_3_PO_4_ gel electrolyte	421.7 F cm^−3^ (343.6 F g^−1^ and 47.8 F cm^−2^ at 0.5 A cm^−3^ (strip‐shaped electrode) Device 3.9 F g^−1^	9.56 mWh cm^−3^	2.91 W cm^−3^	Stable within 10 000 cycles	81% after immersion in water for 48 h	[421]
Polyester fabric (2016)	Two pieces of MnO_2_ deposited conductive graphene/polyester composite fabric with PVA‐NaNO_3_ gel electrolyte	265.8 F g^−1^ at 2 mV s^−1^				87% after 100 times of bending	[465]
Polyester fabric (2018)	PPy electrochemically deposited on rGO painted SnCl_2_ modified polyester textiles with PVA/H_2_SO_4_ gel electrolyte	Electrode 1117 mF cm^−2^ at 1 mA cm^−2^, 329.5 F g^−1^ at 1 mA cm^−1^, Device 474 mF cm^−2^	0.0658 mWh cm^−2^ at 1 mA cm^−2^	25 mW cm^−2^	100% after 10 000 cycles	98.3% after 1000 bending cycles	[408]
Polyester fabric (2019)	Dipping and drying of PET with GO, followed by chemical reduction of GO and subsequent chemical growth of PPy on PET. Composite electrodes of PET/rGO/PPy sandwiched using PVA‐H_2_SO_4_ gel electrolyte.	230 mF cm^−2^ at 1 mV s^−1^, 5.5 F cm^−3^ at 1.6 mA cm^−3^	11 µWh cm^−2^	0.03 mW cm^−2^	76% after 6000 cycles		[466]
Polyester fabric (2020)	Pre‐treated polyester dip coated in GO, followed by reduction of GO and in situ polymerization of PPy particles on fabric surface with aqueous 1 m H_2_SO_4_ solution	Electrode 8300 mF g^−1^ and 640 mF cm^−2^					[467]
Polyester fabric (2020)	Silver paste printed on PET, MnHCF‐MnOx/GO ink overprinted and reduced to form the electrode, PVA/LiCl electrolyte, and paper separator	16.8 mF cm^−2^	0.5 mWh cm^−2^	0.0023 mW cm^−2^		Stable capacitance even while bending to angles 60°, 90°, and 180° for 100 cycles	[468]
Silk fabric (2016)	Current collector and the active materials layers were screen printed [MnO‐coated hollow carbon microspheres, acetylene black, and binder) mixed in a weight ratio of 7:2:1 as printing ink] on a commercial silk fabrics substrate and a PDMS film, respectively. After being pasted with gel electrolyte, the PDMS‐based electrode was transferred exactly on top of the silk fabric‐based one.	19.23 mF cm^−2^ at 1 mA cm^−2^			84% after 2000 cycles	98.5% after 100 times bending and 96.8% after 100 times twisting	[422]
Nylon/PU (67/33) (2021)	Supersonic spraying rGO/SnO2 on fabric with 2 m KOH electrolyte	1008 mF cm^−2^ at 1.5 mA cm^−2^			93% after 10 000 cycles		[423]
Stretchable textiles (2018)	Fully printed Ag@PPy@MnO_2_ on Ag cathode electrode and activated carbon on Ag anode electrode with PVA/Na_2_SO_4_ electrolyte	426.3 mF cm^−2^ (cathode)	0.0337 mWh cm^−2^ at 0.38 mWcm^−2^		90.8% after 5000 cycles	86.2% after 40% stretching strain	[424]
Spandex fabric (2019)	Assembling vertical Polypyrrole nanotube (VPPyNT) grown on the carbon nano onions (CNO) @ Polypyrrole granula (PPyG)‐textile electrodes with a PVA/H_3_PO_4_ gel electrolyte into a sandwiched structure	64 Fg^−1^	5.7 Wh Kg^−1^		85% after 1000 cycles	99% retention after stretching for 500 cycles at a strain of 50%, 88% at a strain of 100%	[469]
Carbon nanofiber web (2008)	PPy was coated on multi‐walled carbon nanotube (CNT)‐embedded activated carbon nanofibers (PPy/ACNF/CNT) using Ni foil as the current collector with 6 m KOH aqueous solution electrolyte for fabric SC electrode	333 Fg^−1^ at 1 mA cm^−2^, 275 Fg^−1^ at 10 mA cm^−2^			Negligible change after 500 cycles		[470]
Graphene fiber fabric (2017)	Hierarchical graphene fiber fabrics (GFFs) with significantly enlarged specific surface area using a hydrothermal activation strategy with (PVA)/H2SO4 gel electrolyte	Electrode 244 Fg^−1^, 1060 mF cm^−2^ at thickness of 150 µm, magnified up to 7398 mF cm^−2^ by overlaying 5 layers	23.5 µWh cm^−2^	26.3 mW cm^−2^	Good rate capability up to 50 000 cycles	Bendable to 180° without breaking, CV barely changed while bending and releasing 300 cycles	[300]
Carbon fabric (2012)	Carbon fabric‐aligned carbon nanotube/ MnO_2_/ conductive polymers (CF‐ACNT‐MnO_2_‐PEDOT) with 1 m Na_2_SO_4_ Composite SC electrodes	1.3 F cm^−2^ at 0.1 mV s^−1^			5% loss after 1000 cycles		[425]
Carbon cloth (2013)	TiO_2_ NWs grown on carbon cloth, Hydrogen‐treated TiO_2_ NWs as core, electrochemically active MnO_2_ (H‐TiO_2_@MnO_2_ as positive) and carbon shells (H‐TiO_2_@C as negative) electrodes with separator, PVA/LiCl gel and LiCl aqueous electrolytes	0.68 F cm^−3^ (PVA/LiCl gel), GCD 0.70 F cm^−3^ at 0.5 mA cm^−2^ (139.6 Fg^−1^ at 1.1 A g^−1^ (LiCl electrolyte)	0.3 mWh cm^−3^ (59 Wh kg ^−1^)	0.23 W cm^− 3^ (45 kW kg^−1^)	91.2% after 5000 cycles	No decay in bending & twisting	[471]
Carbon cloth (2015)	MnO_2_ as cathode, Ti‐Fe_2_O_3_@PEDOT as anode with NKK separator, and PVA/LiCl gel electrolyte	Anode 1.15 Fcm^−2^ (311.6 F g^−1^ and 28.8 Fcm^−3^ at 1 mAcm^−2^) SC 365.8 mF cm^−2^ at 10 mV s^−1^, 444.2 mF cm^−2^ at 1 mA cm^−2^. 2.40 F cm^−3^ at 1 mA cm^−2^	0.89 mWh cm^−3^ at 1 mA cm^−2^	0.44 W cm^−3^	85.4% after 6000 cycles		[472]
Carbon cloth (2015)	SnO_2_@MO_x_ (SnO_2_@NiO, SnO_2_@Co_3_O_4_, SnO_2_@MnO_2_) heterostructures grown on carbon cloth (CC) with 1 m Na_2_SO_4_ aqueous electrolyte (for SnO_2_ and SnO_2_@MnO_2_) and 1 m KOH aqueous electrolyte (for SnO_2_@NiO and SnO_2_@Co_3_O_4_)	SnO_2_@MnO_2_ heterostructure electrode showed the highest discharge areal capacitance (980 mF cm^−2^ at 1 mA cm^−2^)			After 6000 cycles, SnO_2_@Co_3_O_4_, SnO_2_@NiO, and SnO_2_@MnO_2_ electrodes can maintain 58.3%, 63.8%, and 78.1% of its initial SC, respectively at 1 mA cm^−2^		[473]
Carbon cloth (2015)	Two symmetric freestanding polyaniline‐cobalt‐based metal‐organic framework (MOF) crystals (PANI‐ZIF‐67) electrodes with H_2_SO_4_/PVA as gel electrolyte	Areal capacitances 2146 mF cm^−2^ for PANI‐ZIF‐67‐CC electrode at scan rates of 10 mV s^−1^, For the device 35 mF cm^−2^ at the scan rate of 0.05 mA cm^−2^	0.0161 mWh cm^−3^ (0.0044 mWh cm^−2^)	0.833 Wcm^−3^ (0.245 Wcm^−2^)	80% after 2000 cycles		[474]
Carbon cloth (2015)	Assembly of MnO_2_@Carbonized Polypyrrole (CPPy) as positive and carbon coated Co_3_O_4_ (Co_3_O_4_@C) microsheet as negative electrode with PVA/KOH electrolyte	59.5 F cm^−3^ at a current density of 20 mA cm^−2^	27.0 mWh cm^−3^	1.31 W cm^−3^	96% after 5000 cycles	97% after 500 bending cycles	[303]
Carbon cloth (2015)	Hierarchical structure of ALD Co_3_O_4_ nanolayer successfully deposited on CVD derived flexible carbon cloth (CNT/CC), (CNTs@Co_3_O_4_/CC) with 2 m KOH is used as electrolyte	Highest 416.7 mF cm^−2^, 347.2 mFcm^−2^ at 1.25 mA cm^−2^			No capacity fading is observed even after 50 000 cycles		[82]
Carbon cloth (2016)	In situ electrodeposition of MnO_2_ and PPy composite on carbon cloth with 1 m Na_2_SO_4_ aqueous electrolyte	Electrode 325 F g^−1^ at a current density of 0.2 A g^−1^, 228 mF cm^−2^ at 0.14 mA cm^−2^			≈96% after over 1000 cycles		[393]
Carbon cloth (2017)	SnO_2_ nanoparticles, CNTs, ethyl cellulose, and terpineol composite ink screen‐printed onto carbon cloth. Furnace‐calcined SnO_2_/CNT electrodes sandwiched with PVA/H_2_SO_4_ gel electrolyte	5.61 mF cm^−2^ (flat) and 5.68 mF cm^−2^ (bent)					[475]
Carbon cloth (2018)	Solvothermal MoO_2_ coating of carbon fibers followed by covered and interconnect by rGO film to form an electrode with 1 mol L^−1^ H_2_SO_4_ aqueous electrolyte	Electrode 8132 mF cm^−2^ at 2 mVs^−1^	143 µWhcm^−2^ at 10 mA cm^−2^ (corresponding to a power density of 2176µWcm^−2^)	15 022 µWcm^−2^ at 100 mA cm^−2^ (energy density 56.5 µWhcm^−2^)	95% after 30 000 cycles at 120 mA cm^−2^	77% after 6000 times folding tests	[426]
Carbon cloth (2018)	The MnO_2_/cotton derived carbon cloth (CDCC) as positive, CDCC as negative electrode attached by nickel foams, cotton woven separator sandwiched with 1 m Na_2_SO_4_ aqueous electrolyte	202 mF cm^−2^ at 0.1 mA cm^−2^	30.1 mWhcm^−2^ at 0.15 mWcm^−2^		87.7% after 5000 cycles		[476]
Carbon cloth (2019)	Hydrothermal incorporation of Pt into MoS_2_ nanosheets grown on carbon cloth as electrode in 1 m Na_2_SO_4_ solution, Pt‐doped MoS_2_, and activated carbon electrodes with PVA‐ H_3_PO_4_ electrolyte	250 F g^−1^ at 0.5 A g^−1^ (electrode), 42 F g^−1^ at 0.4 A g^−1^ (device)			87.96% after 3000 cycles		[477]
Carbon fiber textile (CFT) (2019)	Activated porous CFT (APCFT) electrode as anode and TiN@MnO_2_ on CFT as cathode with PVA‐LiCl electrolyte	Anode 1.2 F cm^−2^ at 4 mA cm^−2^	4.70 mWh cm^−3^	2.29 W cm^−3^	No capacitance decay after 25 000 cycles		[337]
Carbon fabric (2019)	Two, rGO enfolded cobalt (II, III) oxide nanowires on flexible carbon fabric substrate (CONW‐RGO) electrodes with PVA/KOH gel electrolyte and filter paper as a separator	Electrode 1110 Fg^−1^ at 1 Ag^−1^, SC 111.35 Fg^−1^, 33.4 mF cm^−2^ and 685 mF cm^−3^	34.78 Wh kg^−1^ or 0.0104 mWh cm^−2^ or 0.214 mWh cm^−3^ at power density 750 W kg^−1^ or 0.225 mW cm^−2^ or 4.6 mW cm^−3^	3.6 kW kg^−1^ or 1.2 mW cm^−2^ or 23 mWcm^−3^ at 24.68 Wh kg^−1^ or 0.007 mWh cm^−2^ or 0.16 mWh cm^−3^	Electrode 94.2% after 2000 cycles		[478]
Carbon cloth (2020)	One‐step hydrothermal method to prepare NiMnO3 nanosheets on a carbon cloth (CC) with 6 m KOH was the electrolyte	2330 F g^−1^ at 1 A g^−1^			67.8% after 1000 cycles at 10 A g^−1^		[427]
Carbon cloth (2021)	MoS_2_/PANI composite material drop cast on functionalized carbon cloth; MoS_2_/PANI/FCC electrodes soaked with 1 m H_2_SO_4_ electrolyte	452.25 F g^−1^ at 0.2 A g^−1^ (electrode), 72.8 F g^−1^ at 0.2 A g^−1^ (device)			87% after 1000 cycles at 10 A g^−1^ (electrode)		[371]
Carbon fabric (2022)	Cobalt doped MoS_2_ (Co‐MoS_2_) nanoflower electrode with 1 m KOH electrolyte	86 F g^−1^	4.30 Wh kg^−1^	0.6 KW kg^−1^	98.5% after 10 000 cycles		[479]
Multidimensional hierarchical fabric (2019)	Graphene nanosheets (GNS) and PEDOT: PSS deposited on the hierarchical fabric via spraying method to fabricate flexible SCs with PVA/H_2_SO_4_ gel electrolyte	245.5 mFcm^−2^ at 1 mVcm^−2^	21.82 µWhcm^−2^ at 0.4 mWcm^−2^		83.9% after 10 000 cycles	mere capacitance loss under different bending states	[480]

The pseudocapacitive properties of PEDOT:PSS in combination with PPy were utilized to obtain a cotton fiber‐based 1D SC device. Ma et al.^[^ ^405^ ^]^ blended short‐staple length SSFs with cotton fibers to spin SSF/cotton blended yarn. PPy was deposited on PEDOT:PSS coated composite yarn, followed by a coating with PVA/H_3_PO_4_ electrolyte. Two pieces of yarns were placed in parallel and twisted together to produce a solid‐state two‐ply nanocomposite yarn SC. The cotton‐based SC device exhibited maximum areal capacitance of 1360 mF cm^−2^ at 0.16 mWh cm^−2^ energy density. In another study, Yang et al.^[^ ^406^ ^]^ reported one of the highest gravimetric capacitances (≈506.6 F g^−1^ at 1 A g^−1^) on cotton‐based 1D SC electrode, where PPy and MXene composite was grown on cotton fiber to prepare fiber‐based electrodes. Li et al.^[^ ^407^ ^]^ obtained the highest volumetric capacitance of 221.9 F cm^−3^ at a current of 50 mA, by modifying GO nanosheets (NSs) with ultrathin and large area MoS_2_ NSs followed by reduction and using PVA‐H_3_PO_4_ electrolyte to produce all‐solid‐state hybrid fiber shaped SC. Such composite devices also retained 100% capacitance even after bending at 30 and 60 degrees.

Unlike cotton fiber or yarn‐based SCs, only a very few reports are available on hybridizing carbonaceous compounds with conductive polymers or metal oxides for producing 1D fiber or yarn‐shaped SCs based on synthetic textiles including polyester,^[^ ^408^ ^]^ polyaniline,^[^ ^409^ ^]^ nylon,^[^ ^232^ ^]^ and some elastic^[^ ^229^, ^230^, ^410^ ^]^ fibers. For example, Zhang et al.^[^ ^411^ ^]^ reported a high specific capacitance of 278.6 mF cm^−2^ for stretchable textiles (CNTF). The process involved electrochemical activation of pristine CNT fibers (OCNTF) and coating of PEDOT:PSS, followed by electrochemical deposition of MnO_2_ to form MnO_2_@PEDOT:PSS@OCNTF positive electrode and hydrothermal synthesis of MoS_2_ on CNTF to form MoS_2_ @CNTF negative electrode, with LiCl‐PVA gel electrolyte placed on a stretchable substrate.

Among carbon‐based materials, CNT and graphene alone and/or in combination with other active materials were studied for 1D fiber or yarn‐shaped SCs. Wang et al.^[^ ^412^ ^]^ reported biscrolled MXene with CNTs, which was used to prepare a freestanding asymmetric yarn SC prototype via pairing biscrolled RuO_2_ yarns with 3 m H_2_SO_4_ electrolyte. The areal, volumetric, and gravimetric capacitance of the yarn electrode were reported as high as 3188 mF cm^−2^, 1083 F cm^−3^ and 523 F g^−1^, respectively, at a current density of 2 mA cm^−2^. The SC device also exhibited high energy and power densities of 61.6 mWh cm^−3^ (168 µWh cm^−2^ and 8.4 µWh cm^−1^) and 5428 mW cm^−3^ (14.8 mW cm^−2^ and 741 µW cm^−1^), respectively, with outstanding cycle stability of ≈90% up to 10 000 charge‐discharge cycle. Similarly, He et al.^[^ ^413^ ^]^ reported electrolyte mediation of hybrid fiber made of rGO and MXene, which were assembled into fibers via wet spinning with PVA‐H_2_SO_4_ electrolyte. Such hybrid fiber provided an areal and gravimetric capacitance of 550.96 mF cm^−2^ and 110.89 F g^−1^, respectively at 20 mV s^−1^. The device also exhibited energy and power densities of 12 µWh cm^−2^ and 9.85 mWh cm^−3^ at power densities of 8.8 mW cm^−2^ and 7.1 W cm^−3^, respectively. Additionally, Zhang et al.^[^ ^414^ ^]^ reported Ti_3_C_2_T_x_ in combination with PEDOT:PSS to form wet‐spun hybrid fiber electrodes for SC applications. Such electrodes provided areal, gravimetric, and volumetric capacitance of 676 mF cm^−2^, 258 F g^−1^, and 615 F cm^−3^, respectively. The device fabricated with PVA/H_2_SO_4_ electrolyte achieved an energy density of ≈7.13 Wh cm^−3^ and ≈8249 mW cm^−3^ with excellent stability of ≈95% after 10 000 charge‐discharge cycles. The device showed outstanding flexibility of 96% when stretched to 100% strain. Furthermore, Tao et al.^[^ ^415^ ^]^ reported carbon fibers or yarns‐based 1D SC with a volumetric capacitance of 69.3 F cm^−3^ at 0.1 A cm^−3^. A hybrid structure was prepared by depositing PPy on MnO_2_ nanoflakes coated carbon fiber (CF/MnO_2_/PPy). Two PPy‐MnO_2_‐CFs were fixed on a preservative film substrate and assembled into a SC by sandwiching with PVA/H_3_PO_4_ membrane as a separator and electrolyte between electrodes. Naderi et al.^[^ ^416^ ^]^ drop coated PEDOT:PSS‐ rGO and deposited MnO_2_ on carbon fiber to form yarn‐shaped SC. MnO_2_/PEDOT:PSS‐rGO and PEDOT:PSS‐rGO were used as positive and negative electrodes, respectively, with Na_2_SO_4_‐CMC as a solid‐state electrolyte. The electrodes exhibited capacitance of 2.92 F cm^−2^ (194 F cm^−3^, 550 mF cm^−1^) at 5 mA cm^−2^, which is the highest among such SCs. The energy and power densities were reported as 295 µWh cm^−2^ (19 mWh cm^−3^, 55 µWh cm^−1^) and 2900 µWcm^−2^ (190 mW cm^−3^, 545 µWcm^−1^), respectively. The device was also able to retain 96% of its initial capacitance after 5000 cycles.

Platinum,^[^ ^80^ ^]^ gold,^[^ ^417^ ^]^ and stainless‐steel fiber yarns^[^ ^299^ ^]^ were also studied as wearable SC substrates. For example, Huang et al.^[^ ^418^ ^]^ deposited rGO hydrothermally, and MnO_2_ and PPy electrically on spun stainless steel yarn. The PPy@MnO_2_@rGO‐conductive yarns worked as both active materials and current collectors. The length and areal capacitances made from this yarn were reported as 36.6 mF cm^−1^ and 486 mF cm^−2^ in aqueous Na_2_SO_4_ electrolyte or 31 mF cm^−1^ and 411 mF cm^−2^ in all solid‐state PVA/H_3_PO_4_ electrolyte. The capacitance retained up to 92% over 4950 charge‐discharge cycles, and even 80% after 1000 cycles at 90 bending, 91% after 1000 cycles knotting, and 103% after 1000 cycles twisting.

For 2D fabric‐shaped SCs, conventional textiles such as cotton, polyester, polycotton, and carbon textiles have widely been studied. In such SC devices, carbonaceous compounds are used in combination with conductive polymers and/or metal oxides for SC fabrication via various fabrication techniques. Huang et al.^[^ ^419^ ^]^ electro‐spun carbon nanoweb on nickel‐coated cotton fabrics (Ni–cotton). The as‐prepared fabric SC device achieved an areal capacitance of 973.5 mF cm^−2^ at 2.5 mA cm^−2^. A simple dip‐drying method was reported by Etana et al.^[^ ^420^ ^]^ for fabricating cotton textiles‐ based SC, where GO was deposited by a “dip and dry” method and chemically reduced to form rGO/cotton fabric. MnO_2_ nanoparticles were accumulated on rGO/cotton fabric by in situ chemical deposition, and then PANI layer was coated. With 1 m H_2_SO_4_ electrolyte solution, the SC provided high gravimetric capacitance of 888 Fg^−1^ and high areal capacitance of 444 F cm^−2^, the highest reported for such SC configuration. In another study by Li et al.,^[^ ^408^ ^]^ PPy was electrochemically deposited on rGO painted and SnCl_2_‐modified polyester textiles, which provided areal capacitance of 1117 mF cm^−2^ at a current density of 1 mA cm^−2^ with 100% retention after 10 000 cycles. The SC device fabricated from such fabric electrodes and PVA/H_2_SO_4_ gel electrolyte exhibited an aerial capacitance of 474 mF cm^−2^. Cheng et al.^[^ ^421^ ^]^ developed strip‐shaped composite electrodes, which were prepared via depositing PANI on aligned CNTs, exhibiting a high volumetric capacitance of 421.7 F cm^−3^.

In addition to polyester fabrics, silk,^[^ ^422^ ^]^ nylon,^[^ ^423^ ^]^ and stretchable^[^ ^424^ ^]^ textiles have also been studied for the fabrication of 2D fabric‐shaped SCs. However, carbon fiber fabric has widely been used as SC substrate by several research groups.^[^ ^425^, ^426^, ^427^ ^]^ For instance, Lv et al.^[^ ^425^ ^]^ reported a composite SC electrode prepared by depositing aligned CNT/MnO_2_/conductive polymers on carbon fabric (CF‐ACNT‐MnO_2_‐PEDOT). With 1 m Na_2_SO_4_ electrolyte, the composite electrodes achieved a high areal capacitance of 1300 mF cm^−2^ at 0.1 mV s^−1^. Though several attempts were reported for the enhancement of the capacitive performance, the highest areal capacitance for carbon fabric‐based SC was reported by Zhu et al.^[^ ^426^ ^]^ They coated carbon fibers MoO_2_ by solvothermal method; covered and interconnected with rGO film to form SC electrode. The areal capacitance of the electrode was reported as high as 8132 mF cm^−2^ at 2 mVs^−1^. The electrode also retained 95% capacitance after 30 000 charge‐discharge cycles at 120 mA cm^−2^ and 77% after 6000 times folding tests. However, the highest gravimetric capacitance was reported by Mu et al.,^[^ ^427^ ^]^ who hydrothermally prepared NiMnO_3_ nanosheets on a carbon cloth. With 6 m KOH electrolyte, the electrodes reached 2330 Fg^−1^ at the current density of 1 Ag^−1^.

## 7 Other Key Properties of SCs for Wearable Applications

### 7.1 Flexibility

To be considered as wearable, a SC device must be flexible and durable under the physical movement of the body. The bending of SC devices for hundred to several hundred cycles is performed at various angles to evaluate the flexibility of a SC device. Additionally, twisting, winding, and other deformations are also assessed. Yu et al.,^[^ ^351^ ^]^ reported more than 97% capacitance retention after 1000 bending cycles at a 90° angle of hierarchically structured CNT‐graphene fiber‐based micro SCs. Chen et al.^[^ ^229^ ^]^ reported an electrochromic fiber‐shaped SC composed of elastic fiber/CNTs/PANI retaining capacitance of 93.8% after 1000 bending cycles at 180°. Choi et al.^[^ ^433^ ^]^ reported a flexible SC made of CNT yarn with MnO_2_ exhibiting no decrease of capacitance even after 1000 bending at 90°. Ding et al.^[^ ^439^ ^]^ fabricated a graphene/PPy composite fibers for all‐solid‐state, flexible fiber form SC that exhibited similar performance.

**Figure 18:**
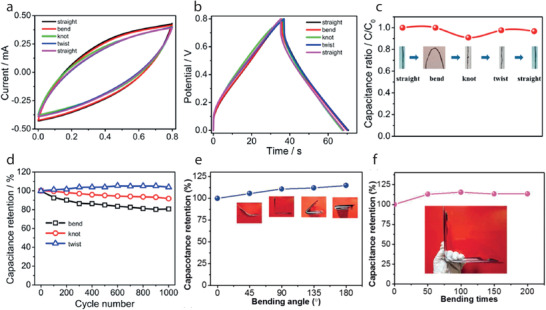
Flexibility tests of PPy@MnO_2_@rGO‐deposited conductive yarns measured in the two‐electrode cell. a) CV curves of the all‐solid‐state yarn supercapacitor undergoing consecutive deformations at a scan rate of 100 mV s^−1^.b) GCD curves of the all‐solid‐state yarn supercapacitor undergoing consecutive deformations at a current density of 80 mA cm^−3^. c) Capacitance ratio under various deformations. d) Capacitance retention of the all‐solid‐state yarn supercapacitor after each deformation. Reproduced with permission.^[^ ^418^ ^]^ Copyright 2015, American Chemical Society. Influence of bending deformation on CeO_2_‐ACVF capacitive performance; specific capacitance e) under various bending angles; and f) after different bending cycles. ACVF: activated viscose fabric. Reproduced with permission.^[^ ^396^ ^]^ Copyright 2020, SAGE Publications.

In addition to bending, the flexibility of SCs for other mechanical deformations was reported by some research groups. For example, a yarn‐based SC was developed by Lee et al.,^[^ ^448^ ^]^ composed of Pt/MWCNTs/PEDOT retained 98% of its initial capacitance after 2000 bending, 92% after 10 000 winding, and 99% after 10 000 cycles when woven into a glove. Huang et al.^[^ ^418^ ^]^ reported yarn‐based SC of rGO/MnO_2_/PPy that retained 80% capacitance after 1000 cycles at 90° bending, 91% after 1000 cycles of knotting, and 103% after 1000 cycles of twisting, revealing the enhancement of the capacitive performance, **Figure** **18**(a–d). Similarly, the capacitance was increased (107% retention) after 1000 cycles of twisting for an asymmetric fiber‐shaped solid‐state SC based on carbon fiber bundle.^[^ ^444^ ^]^ Additionally, several other articles have reported higher bending cycles. For example, Ye et al.^[^ ^430^ ^]^ reported a fiber‐shaped SC by introducing rGO and carbon nanoparticles (CNPs) on commercial CTs using dip‐coating technique combined with low‐temperature vapor reduction, which resulted in 92.30% capacitance retention after 2000 bending cycles. Liu et al.^[^ ^429^ ^]^ examined their 1D‐shaped flexible yarn SC composed of rGO/Ni cotton composite electrodes with PVA/LiCl gel as electrolyte and separator for 4000 cycles, and found 95% of retention after at 180° bending angle. Furthermore, Wu et al.^[^ ^435^ ^]^ reported a flexible fiber‐shaped SCs (FSSCs) by twisting a number of CNT yarns (n) with a Pt filament as current collector and PANI nanowires. They obtained a capacitance retention of 98.17% after 3000 cycles and 95.91% after 5000 flexing cycles.

For 2D‐shaped SC devices, shorter bending cycles were used for testing capacitance retention of textile SCs. Zhang et al.^[^ ^422^ ^]^ reported a silk fabric‐based SC which retained 98.5% after 100 bending cycles and 96.8% after 100 twisting cycles. Lee et al.^[^ ^303^ ^]^ developed an asymmetric SC by assembling MnO_2_@CPPy and carbon coated Co_3_O_4_ microsheet (Co_3_O_4_@C)‐decorated carbon cloths with a solid‐state PVA/KOH electrolyte, which retained 97% capacitance after 500 bending cycles. Luo et al.^[^ ^396^ ^]^ reported an increase of 13.4% capacitance after 200 bending cycles for an all‐fabric solid‐state flexible SC, made of activated carbon fiber fabric, Figure 18(e,f). The capacitance of a solid‐state stretchable SC, prepared by assembling VPPyNTs/CNOs@PPyG‐textile electrodes with a PVA/H_3_PO_4_ gel electrolyte into a sandwiched structure, was nearly unchanged after stretching for 500 cycles at a strain of 50%. However, the capacitance retention ratio decreased slightly to 88% as the strain% was increased to 100%.^[^ ^469^ ^]^

### 7.2 Safety Issue

To be wearable, the flexible SC device components must be non‐toxic to avoid any health concerns as well as for the environment when disposed of. The concerns with wearable SCs are raised due to the toxic nanoparticles or metal particles of all sizes entering or generated during manufacturing stages as well as during usage. In addition, the effect of electromagnetic fields, accidental electric shock, and the inability to activate the emergency shut‐off in case of malfunctioning are also matters of concern.^[^ ^481^ ^]^ In addition, wearable SCs and other wearable electronic devices have limited lifetimes. Therefore, it is also critical to ensure that the waste generated by the SCs does not create new hazards for health and the environment.

A report was published revealing that more than 8 billion batteries enter the US and European markets annually. Also, 3 billion alkaline units get discarded each year in North America alone.^[^ ^333^ ^]^ Another report projected a generation of more than 130 g of battery waste per person each year.^[^ ^482^ ^]^ Besides battery, increasing usage of mobile, computing, and other autonomous electrical devices increases the production and disposal of SC devices exponentially.^[^ ^333^ ^]^ An estimated 20 000 tonnes of old household batteries end up in landfill every year.^[^ ^483^ ^]^ The challenges of such disposal to the environment are due to the presence of a large number of toxic metals (e.g., Cd, Ni, Pb), F‐containing electrolytes and device components, corrosive fluids (e.g., H_2_SO_4_ and H_3_PO_4_), and fire hazards from organic electrolytes, which may have negative environmental impacts and may induce numerous health problems such as acute or long‐term exposure. The principal issue of the release of metals into landfills is the potential to contaminate the groundwater. Incineration of them may also pose two major potential environmental concerns; the release of metals (mostly mercury, cadmium, and lead) into the ambient air and the concentration of metals in the ashes which must be landfilled. The F‐containing electrolyte salts (tetraethylammonium tetrafluoroborate, Net_4_BF_4_), carbon particle binder (such as PTFE or PVDF), and electrode separator (often PTFE) are likely to generate volatile fluorocarbons during traditional incineration, which are highly toxic to organisms and are likely to damage incinerators and nearby structures. Acetonitrile solvents, commonly implemented in high‐performance devices, are flammable, carcinogenic, and may decompose into highly toxic cyanides upon heating. Some ions commonly used in promising ILs, such as bis(trifluoromethanesulfonyl)imide (TFSI), have been shown to inhibit cellular respiration. Although some aqueous electrolytes that implement Li_2_SO_4_ or Na_2_SO_4_ are expected to be benign to the environment, they still emit SO_2_, contributing to acid rain when released during incineration. Although SCs, unlike fuel cells and batteries, contain no noble or heavy metals that are particularly difficult to dispose of, conventional collectors and packaging materials, such as steel and Al, are incombustible and cannot be fully burned without leaving ash residue. Thus, the disposal of SCs not only generates harmful substances but also incombustible waste materials that need to be stored in a landfill.^[^ ^482^ ^]^

### 7.3 Washability

Washability is a product's ability to withstand a predetermined number of cycles of a specified washing process, able to adequately clean the product without loss of functionality and/or serviceability, and without resulting security risks for the user.^[^ ^484^ ^]^ Most e‐textiles still suffer from poor wash ability, reducing the reliability of e‐textiles to be ready for the market. Many experimental wearable e‐textiles are not suitable for real‐life applications because of this problem. The hydrophobic textile substrate, due to the capillary effect, can still absorb water in the textile bulk making the device fail. Also, the mechanical stresses incorporated by the washing cycles may destroy the electrical contacts between the conductive thread and the electronic wearable device. Thus, the electric impedance becomes uncontrollable after several washing cycles, making the wearable device unstable and often stops functioning.^[^ ^485^ ^]^ Therefore, there remains a need for technology that can provide better wash stability for conductive textiles.

**Figure 19:**
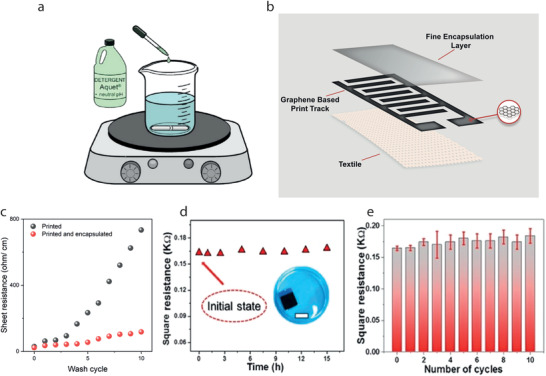
a) Schematic diagram of washing test. Reproduced with permission.^[^ ^486^ ^]^ Copyright 2019, The Royal Society of Chemistry. b) Illustration of graphene‐based ink pattern and encapsulation layer on textile substrate. c) The change in electrical resistance with number of washing cycles of graphene‐based ink printed (without encapsulation) and graphene‐based ink‐printed (with encapsulation) cotton fabric. Reproduced with permission.^[^ ^358^ ^]^ Copyright 2022, Elsevier. d) Resistances of electrode after being immersed in water for different times. The inset is the photograph of electrode immersed in water for 1 week (scale bar:, 20 mm). e) Resistances of same electrode on nylon substrate after being immersed in water for different times and 2 h for each time. Reproduced with permission.^[^ ^487^ ^]^ Copyright 2018, American Chemical Society.

Wash ability is usually reported by the retention of performance after several washing cycles. We encapsulated screen‐printed graphene‐based conductive patterns on textiles to protect them from being washed away, **Figure** **19**b.^[^ ^358^ ^]^ The sheet resistance before and after encapsulation of the printed pattern was evaluated. It was found that the bare pattern had an increase of 10 times resistance, whereas the encapsulated pattern exhibited only a 3.5 times increase in the sheet resistance after 10 wash cycle, Figure 19c. Cao et al.^[^ ^487^ ^]^ also reported a screen‐printed washable e‐textile electrodes, which were tested after being immersed in water for different times, showed very negligible variation, after soaking in water repeatedly and for a longer duration, Figure 19(d,e). A KAIST research team, fabricated a self‐powered washable textile‐based wearable display module on real textiles that integrate polymer solar cells (PSCs) with organic light emitting diodes (OLEDs), exhibiting little change in characteristics after 10 min‐long 20 washings cycles.^[^ ^486^ ^]^ Qiang et al.^[^ ^360^ ^]^ demonstrated a super‐hydrophobic conducting fabric with graphene and hexagonal boron nitride inks. The different fabrics were then integrated to engineer an all‐textile‐based capacitive heterostructure that sustained 20 cycles of repeated washing. Barazekhi et al.^[^ ^467^ ^]^ also reported a negligible decrease in conductivity after 20 laundry cycles for rGO – PPy based polyester textile SC.

## 8 Future Research Directions and Conclusion

### 8.1 Towards High‐Performance Energy Storage Textiles

**Figure 20:**
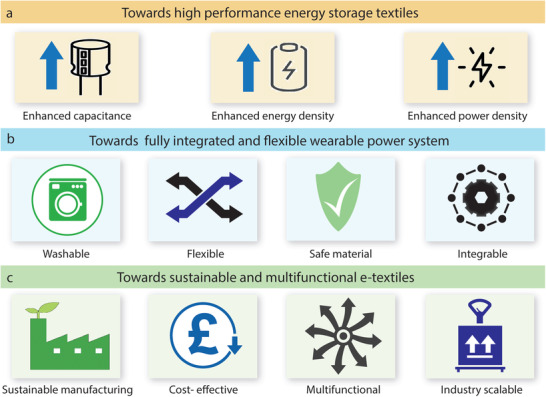
Future research direction of flexible supercapacitors.

The continuous development of various wearable and portable electronic devices integrated with textiles has increased the demand for commercially available textile‐based flexible energy storage devices. According to research by Fact.MR,^[^ ^488^ ^]^ the e‐textiles demand is expected to grow due to the growing demand for equipment that can track body movements. From 2021–2031, the market is expected a rapid growth owing to its expanding applications in health monitoring, sports training, hazardous materials monitoring, military monitoring, etc. The IDTechEx Research forecasts that the market for wearables will reach $138 bn by 2025.^[^ ^489^ ^]^ The Acumen Research and Consulting says e‐textiles and smart clothing market surpass $15 018.9 Mn by 2028 with a CAGR of 32.3%.^[^ ^490^ ^]^ In this review, we have discussed a brief overview of the textile‐based energy storage SCs, from materials to device fabrication. Though various laboratory‐scale devices have already been reported, there remain many challenges for the wide‐scale commercial adaptation of such energy storage textiles. Since the principal function of energy storage textiles is to power up various wearable electronics, the most important challenge is the improvement of energy storage capacity comparable to the existing rigid conventional batteries. Energy densities of SCs are not very high, and there still remains a clear gap between SCs (<20 Wh kg^−1^) and batteries (30–200 Wh kg^−1^) in terms of energy densities.^[^ ^491^ ^]^ The SC with lower energy density will result in bulkier devices that are not compact. An effective way to improve the storage capacity of SCs could be the improvement of manufacturing processes and technology. But in the long run, it is essential and difficult to find new electrolyte and electrode active materials with higher electrochemical performance. Considering the energy formula, [0.5CV^2^], the three main approaches usually taken into consideration to increase the energy density are: improving the capacitance of the system by fine‐tuning the electrode materials’ surface properties, improving the voltage of the device by selecting electrodes, and electrolytes providing wide electrochemically stable potential windows and increasing the capacitance and the voltage at the same time by assembling a faradaic electrode with a non‐Faradaic one, such as a hybrid system.^[^ ^492^ ^]^ The rated voltage of a SC is very low (less than 2.7 V), which requires a lot of series connections for practical applications. Because of the need for high current charging/discharging in applications, and damage of capacitors due to overcharging, it is very important whether the voltages on individual capacitors (in series) are consistent or not.^[^ ^491^ ^]^ However, the increased loading of active material to improve the energy storage capacity always makes the textiles stiff and inappropriate for wearable applications. Therefore, energy storage nanomaterials with a high specific area, high conductivity, and good mechanical properties are attractive for further research and improvement. An energy storage system based on a battery electrode and a SC electrode, called battery‐SC hybrid (BSH), offers a promising way to construct a device with merits of both secondary batteries and SCs.^[^ ^493^ ^]^ Such hybrid battery‐SCs inherit the high power (≈0.1–30 kW kg^−1^) of SCs and the high energy density (≈5–200 Wh kg^−1^) of secondary batteries, with the advantages of stable long cycle performance and low cost. By addressing all these issues appropriately, energy densities of SCs can become comparable to batteries (**Figure** **20**a).^[^ ^491^ ^]^

### 8.2 Towards Fully‐Integrated and Flexible Wearable Power System

#### 8.2.1 Washability

E‐textiles, like other everyday textiles, often need to withstand washing procedures to ensure its general usability, Figure 20b.^[^ ^494^ ^]^ However, washability is seen as one of the main obstacles to reach a wider market of e‐textile products. To assess, improve, and evaluate the extent of an e‐textile's in terms of washing, repeated test cycles are executed. So far, there are no standardized methods for testing the wash fastness of e‐textiles and no protocols to comparably assess the washability of tested products.^[^ ^495^ ^]^ Washing e‐textiles is challenging. The effect of washing on the e‐textile performance relies not only on the type of conductive materials or fabrication process but also on the specific textile substrate used (i.e., materials and constructions) along with their interdependency. Therefore, no global conclusion can be drawn on how a washing program for smart e‐textiles should be configured. Considering textile substrates, the applicability and suitability of different textiles depend on the type of conductive track used while looking to achieve the best washability results. On the other hand, if the choice of textile for a smart textile application is fixed due to specific requirements (such as sufficient elasticity for sports clothing, etc.), the type of conductive track used needed to be adapted accordingly for best reliability results.^[^ ^484^ ^]^

#### 8.2.2 Flexibility

With the rapid advancement of portable electronic products and the concept of wearable electronics, flexible energy storage devices have become popular with researchers. The traditional SCs, however, are greatly restricted to the shape of the device due to the rigid nature of the electrode. Flexible and small SCs with high electrochemical performance compatible with portable electronics can be the direction for next‐generation flexible SCs, Figure 20b. The textile substrate is naturally flexible, however, the flexibility of the electrode material is the prime concern for SC flexibility. Enhancing the electrochemical performance often requires the deposition of additional electroactive material which in return makes the structure rigid. Therefore, there remains still a need for further exploration of suitable electrode materials which could possess ultra‐flexibility while keeping ultra‐high electrochemical performances.

#### 8.2.3 Safety

One of the important considerations of fabricating textile‐based SCs is the utilization of safe electroactive materials. Since such textile‐based devices are aimed at powering other e‐textiles for human health management, the final device must be safe for human body as well for the environment when disposed, Figure 20b. Till now, most used materials for SCs electrodes are generally toxic, environmentally unfriendly, and not biocompatible, and thus need a safer and environmentally friendly substitute.

#### 8.2.4 Integration

Integration of sandwiched battery or SC device in textiles is a challenge due to the accumulation of device components (current collector, electrodes, separator, etc.) onto thin textiles substrate, Figure 20b. This is faced by most single‐layer fabrics, however multi‐layer fabrics such as jackets and winter coats may suffer less from this issue. Only a few reports were published demonstrating the successful weaving or knitting of SC yarns to produce energy storage fabrics. Meeting the strict quality requirements of industrially‐used weaving and knitting machines is one of the main challenges here. Therefore, coating or printing of conductive materials on existing textiles or conductive fibers/yarns to be used directly into full fabrics could be possible solutions to such problems. For fabric‐shaped devices, the traditional cut‐and‐sew method is the simplest method to encapsulate and integrate fabric‐based energy storage devices into final textile products together with other electronic components. Adhesive bonding, ultrasonic welding, and laser welding are other joining methods able to eliminate bulky stitched seams and bring less damage to the electronic components within the devices.^[^ ^33^ ^]^ However, fabrication complexity and cost gradually decrease from a single fiber/yarn to a whole piece of textile. In the meantime, the output performance could be improved by introducing materials with larger electron affinity differences, enlarging the contact area by expanding the dimension, and increasing the distance gap.^[^ ^65^ ^]^

### 8.3 Towards Sustainable Materials and Electronics

#### 8.3.1 Sustainable Manufacturing

The rapid development of the electronic industry has accelerated the demand for high‐performance portable power supply units. In addition, to meet social needs and promote industrial development, the application aspects of SCs have also gained importance. People all over the world, nowadays, have been paying more and more attention to energy consumption and environmental protection, towards the use of more and more clean energy. As a result, the consumer culture around the world has also raised the need for all products to be more sustainable and recyclable to reduce the environmental impact.^[^ ^496^ ^]^ This the SC industries too. Therefore, the need to explore safe and sustainable manufacturing of energy storage devices is an imperative concern of the world today. New eco‐friendly, as well as cost‐efficient energy storage systems, should be developed, in view of the requirements of emerging ecological concerns and modern society, Figure 20c.^[^ ^497^ ^]^

#### 8.3.2 Improving the Cost Performance

The most important consideration for any industry to endure is the improvement of product performance along with the reduction of production costs. Similarly, in SC technology, in addition to improving the manufacturing process and technology, finding stable and effective electrode and electrolyte materials along with reducing the cost is the future research focus. Several approaches could be considered for the cost reduction of any technology. Replacing an existing material with a new low‐cost raw material, such as natural mineral resources is an attractive option. The combination of a low‐price raw materials with high price raw materials without compromising the performance could be another approach to reduce the overall cost. In brief, the development of SCs is inseparable from the progress of science and technology and the demand for application. We believe the development of SCs will also be more rapid and far‐reaching with the popularity of new and smart wearable devices, Figure 20c.^[^ ^491^ ^]^

#### 8.3.3 Multifunctionality

Textile‐based wearable electronics have attracted immense attention during the past few years due to their softness, breathability, and biocompatibility, making them durable and wearable for long‐term application.^[^ ^498^, ^499^ ^]^ The rapid progress of intelligent electronic devices has put people in need of intelligent and controllable multifunctional devices which allow manufacturers and users to program themselves to ease performing different functions to be utilized in fulfillment of different requirements in real life. Several research groups have already reported multifunctionalities in textiles,^[^ ^500^, ^501^, ^502^ ^]^ however, a textile that can monitor several health parameters is of special interest for personalized health care applications.^[^ ^503^, ^504^ ^]^ Tian et al.^[^ ^505^ ^]^ prepared multifunctional e‐textiles with high conductivity by a simple screen‐printing method, which not only exhibit excellent sensing performance, but also have outstanding joule heating performance. As a proof of concept, we reported a flexible and washable screen printed graphene‐based e‐textiles platform that can work as activity sensor, can monitor the brain activity, and can store energy in the form of SC.^[^ ^358^ ^]^ Future development of modern electronics inspires the use of flexible devices for both energy conversion and storage.^[^ ^506^, ^507^ ^]^ An effective solution to the ever‐increasing energy crisis could be the use of solar energy. Excitonic solar cells such as polymer and dye‐sensitized solar cells could convert the solar energy into electric energy which is transferred to electrochemical devices, such as lithium‐ion batteries and supercapacitors, for storage.^[^ ^508^ ^]^ E‐textiles are a promising technology that could soon become part of our everyday lives. However, e‐textiles with a single functionality cannot meet the requirements of electronics. Increasing attention is thus being paid to realizing the functional integration among the generation, storage, and utilization of electricity and the introduction of other functionalities into e‐textiles.^[^ ^509^ ^]^ In the future, the integration of SCs with sensors, actuators, electrochromic, shape memory, and even self‐repair functions will be very attractive for multifunctional and self‐powering personal health management textiles, Figure 20c.

#### 8.3.4 Industrial Scalability

Large‐scale manufacturing ability is an important criterion to be considered for the development of e‐textiles, Figure 20c. The electrode materials to be integrated into the full garment must be scalable for industrial manufacture. Many experimental designs lack this criterion hindering the scope of the device to be used commercially. The major challenge for constructing conductive fiber/yarn is their length‐related resistance. Since the resistance is directly proportional to its length and inversely proportional to its cross‐sectional area, It shows that shorter lengths and larger cross‐sectional areas of such fiber/yarn give lower resistance.^[^ ^510^ ^]^ Therefore, many devices showing low ESR per length may become too resistive beyond a certain working length to act as an energy storage device. Another challenge may arise with the fabrication techniques to form the electrode materials. Many sophisticated fabrication techniques such as ink‐jet printing of conductive materials, and coating by physical or chemical vapor deposition methods may appear feasible for lab‐based experiments but are too expensive for large‐scale production.

## Conflict of Interest

The authors declare no conflict of interest.
